# A Genotypic Test for HIV-1 Tropism Combining Sanger Sequencing with Ultradeep Sequencing Predicts Virologic Response in Treatment-Experienced Patients

**DOI:** 10.1371/journal.pone.0046334

**Published:** 2012-09-27

**Authors:** Ron M. Kagan, Erik P. Johnson, Martin Siaw, Pinaki Biswas, Douglass S. Chapman, Zhaohui Su, Jamie L. Platt, Rick L. Pesano

**Affiliations:** 1 Department of Infectious Diseases, Quest Diagnostics Nichols Institute, San Juan Capistrano, California, United States of America; 2 Pfizer, Collegeville, Pennsylvania, United States of America; 3 Pfizer, New York, New York, United States of America; 4 Outcome Sciences, Cambridge, Massachusetts, United States of America; National Institute of Allergy and Infectious Diseases, United States of America

## Abstract

A tropism test is required prior to initiation of CCR5 antagonist therapy in HIV-1 infected individuals, as these agents are not effective in patients harboring CXCR4 (X4) coreceptor-using viral variants. We developed a clinical laboratory-based genotypic tropism test for detection of CCR5-using (R5) or X4 variants that utilizes triplicate population sequencing (TPS) followed by ultradeep sequencing (UDS) for samples classified as R5. Tropism was inferred using the bioinformatic algorithms geno2pheno_[coreceptor]_ and PSSM_x4r5_. Virologic response as a function of tropism readout was retrospectively assessed using blinded samples from treatment-experienced subjects who received maraviroc (N = 327) in the MOTIVATE and A4001029 clinical trials. MOTIVATE patients were classified as R5 and A4001029 patients were classified as non-R5 by the original Trofile test. Virologic response was compared between the R5 and non-R5 groups determined by TPS, UDS alone, the reflex strategy and the Trofile Enhanced Sensitivity (TF-ES) test. UDS had greater sensitivity than TPS to detect minority non-R5 variants. The median log_10_ viral load change at week 8 was −2.4 for R5 subjects, regardless of the method used for classification; for subjects with non-R5 virus, median changes were −1.2 for TF-ES or the Reflex Test and −1.0 for UDS. The differences between R5 and non-R5 groups were highly significant in all 3 cases (p<0.0001). At week 8, the positive predictive value was 66% for TF-ES and 65% for both the Reflex test and UDS. Negative predictive values were 59% for TF-ES, 58% for the Reflex Test and 61% for UDS. In conclusion, genotypic tropism testing using UDS alone or a reflex strategy separated maraviroc responders and non-responders as well as a sensitive phenotypic test, and both assays showed improved performance compared to TPS alone. Genotypic tropism tests may provide an alternative to phenotypic testing with similar discriminating ability.

## Introduction

In order for the human immunodeficiency virus type 1 (HIV-1) to infect cells, its gp120 envelope glycoprotein must interact with the cellular CD4 receptor and one of two chemokine coreceptors: CCR5 or CXCR4 [1], [2], [3]. HIV-1 variants are classified as CCR5-using (R5), CXCR4-using (X4), or dual-mixed (D/M) based on their ability to utilize one or both coreceptors. ART-naïve patients classified as having D/M virus typically harbor mixtures of R5 and dual and/or X4 virus [4]. R5 virus is more commonly found in the early stages of infection and in treatment-naïve patients, whereas D/M and X4 variants are present in up to 50% of late-stage and treatment-experienced patients [5], [6], [7]. The presence of CXCR4-using virus (D/M or X4) in an infected patient is a predictor of lower CD4+ T-cell count, a higher HIV-1 viral load and a more rapid progression to AIDS [6], [8], [9].

Small-molecule CCR5 inhibitors block the interaction of the HIV-1 envelope gp120 glycoprotein with the CCR5 coreceptor [2]. The CCR5 entry inhibitor maraviroc has proven to be an effective antiretroviral agent in patients harboring exclusively R5-using variants [10], [11], [12] but does not benefit patients harboring CXCR4-using virus [13], [14], [15]. Thus, an HIV-1 tropism test is required prior to CCR5 antagonist administration to exclude from treatment patients harboring non-R5 virus. Tropism can be determined by phenotypic or genotypic testing. Phenotypic assays such as the original Trofile and the more recently offered Trofile Enhanced Sensitivity (TF-ES) from Monogram Biosciences measure the ability of pseudoviruses carrying the entire cloned envelope gene from a patient's virus to infect CD4(+)/CCR5(+) and CD4(+)/CXCR4(+) indicator cells [16], [17]. Although this approach has proven to be sensitive and correlates well to clinical outcomes [10], [14], phenotypic testing is expensive to perform and requires a relatively long turnaround time.

Genotypic approaches to determine tropism have also been developed that utilize population-based Sanger sequencing of the third variable region (V3) of the HIV-1 gp120 envelope glycoprotein, the primary determinant of viral tropism [18]. Bioinformatic algorithms are then used to infer viral tropism [19], [20]. Although these population-based sequencing approaches give reasonable agreement with phenotypic tests to predict viral tropism [21], [22], [23], [24], they are not sensitive enough to detect minor non-R5 variants; this situation is similar to standard genotypic resistance testing for HIV-1 reverse transcriptase and protease mutations. For patients with D/M virus, maraviroc therapy may result in selection of non-R5 virus and treatment failure [13], [15], [25].

Ultra deep sequencing (UDS) on the GS FLX and GS Junior instruments from Roche/454 (Branford, CT) utilizes clonal amplification and sequencing of thousands of individual variants for each sample [26]. This technology provides greater sensitivity than conventional population sequencing to detect minor populations of HIV-1 variants [27], [28]. In a large retrospective analysis of the Maraviroc versus Optimized Therapy in Viremic Antiretroviral Treatment-Experienced Patients (MOTIVATE) trials, UDS identified non-R5 virus in more than twice as many maraviroc recipients as the original Trofile assay [29]. In a retrospective re-analysis of the MERIT trial of treatment-naïve patients comparing maraviroc to efavirenz, UDS showed the same ability as the TF-ES assay to separate maraviroc responders from non-responders [30].

Here we report on the development and the performance of a clinical laboratory-developed tropism test that uses triplicate population sequencing (TPS) and a reflex strategy whereby only samples predicted to be R5 by population sequencing are further tested with the more sensitive UDS assay. We retrospectively evaluated the ability of this testing strategy to predict short term virologic response in a treatment-experienced clinical trial population as a function of tropism status relative to the TF-ES phenotypic assay.

## Results

### Baseline Characteristics of Study Subjects

A total of 363 screening samples from the MOTIVATE and A4001029 trials were included in this study. We successfully performed TPS and UDS for 348 samples and were able to obtain TF-ES results for 327 of these samples. The baseline characteristics for these 327 study subjects stratified by genotypic and phenotypic tropism assay status are shown in Table 1. Most patients were Caucasian, male, median age of 44, and infected with HIV-1 subtype B. The median phenotypic weighted susceptibility score for the number of active drugs in the optimized background regimen (wOBTss) was 1.0. The baseline median viral load was similar regardless of tropism results however the baseline CD4(+) T cell count was lower for subjects predicted to have non-R5 virus by both assays (Table 1, X4/X4 group) or by TF-ES alone (Table 1, R5/X4 group).

**Table 1:** Baseline characteristics of study subjects.

				Tropism Results (GTT/TF-ES)^1^		
Characteristic	Subcategory	R5/R5	R5/X4	X4/R5	X4/X4	Total
N		154	32	30	111	327
Median age (min, max)		43 (30,75)	44.5 (33,56)	44 (26,64)	44 (16,70)	44 (16,75)
Gender^4^	Male	143	30	28	97	298
	Female	11	2	2	14	29
Race/ethnicity^4^	Caucasian	128	26	22	80	256
	Black	20	4	8	29	61
	Other	6	2	0	2	10
HIV-1 subtype^2^^,^ 4	B	144	29	27	103	303
	non-B	10	3	3	8	24
Median (IQR) baseline CD4+ cells/uL		169.5 (62,289)	50.5 (20,140)	116 (36,169)	40 (8,100)	96 (25,222)
Median (IQR) log10 pVL		4.9 (4.3,5.3)	4.9 (4.6,5.5)	4.9 (4.5,5.4)	5.1 (4.8,5.5)	5.0 (4.5,5.4)
wOBTss (min, max)^3^		1 (0,3.5)	1 (0,3.0)	1 (0,3.0)	1 (0,4.0)	1 (0,4.0)

1 Subject characteristics were grouped according to R5 or non-R5 (X4) concordance between the genotypic tropism test (GTT) using triplicate population sequencing with reflex to ultradeep sequencing as described in Methods, and the Trofile Enhanced Sensitivity assay.

2 The HIV-1 subtype was determined by the geno2pheno software from the Envelope V3 loop sequence.

3 Phenotypic uncensored weighted optimized background therapy susceptibility score.

4 There were no significant differences between groups (1–4) in the proportions of subjects who were male vs female, Caucasian vs non-Caucasian and infected with subtype B vs non-B virus (P>0.05).

### Viral Load Changes as a Function of Tropism Status

**Figure 1:**
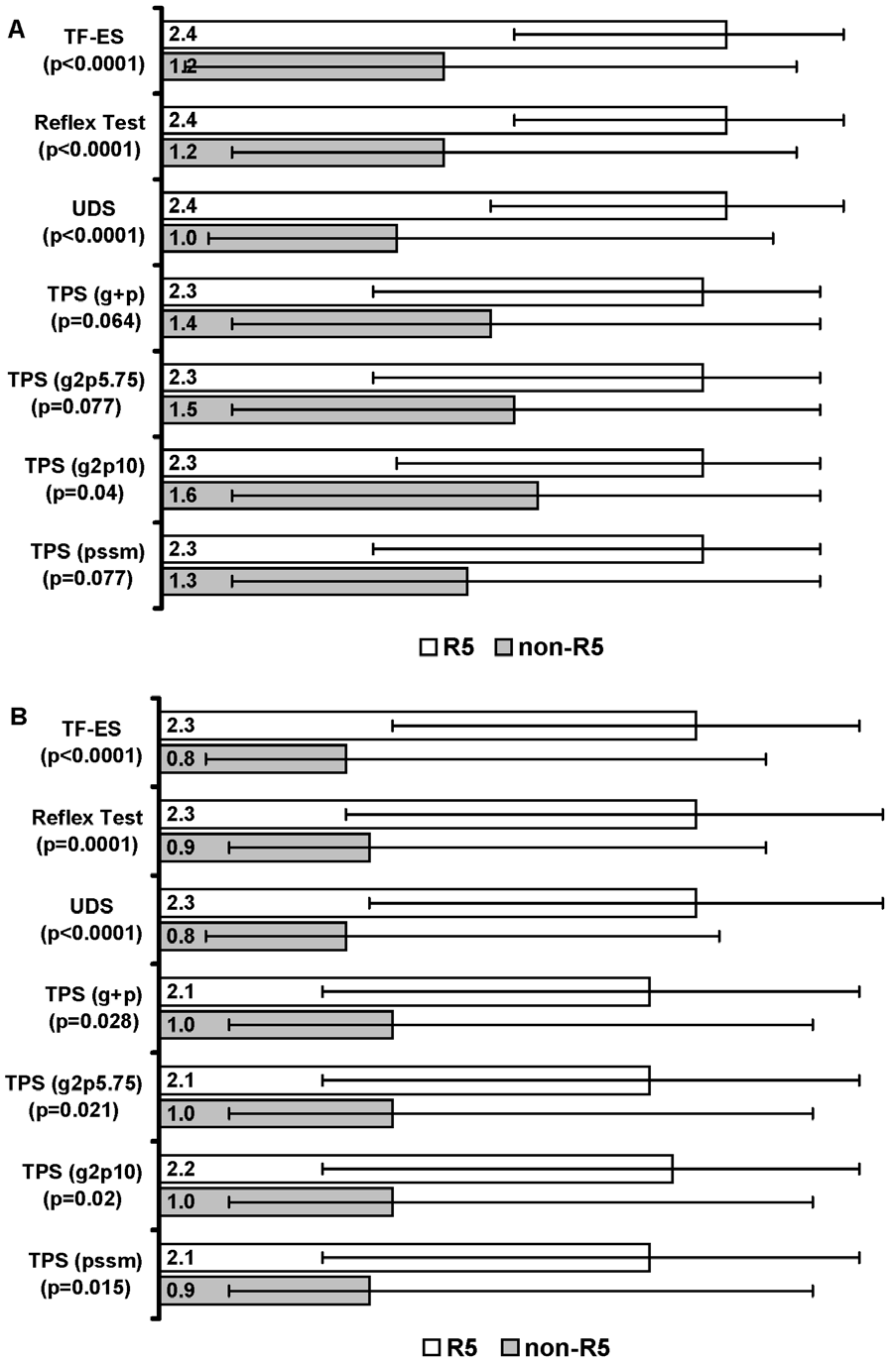
Median log_10_ plasma viral load (pVL) decline at weeks 8 and 24 as a function of tropism status and assay. **A**: Week 8 results. **B**: Week 24 results. The median pVL decline is shown for R5 and non-R5 virus for each assay. Error bars represent the interquartile range. P-values were determined using a two-sided Wilcoxon test. TF-ES: Trofile Enhanced Sensitivity (Monogram Biosciences); Reflex Test: triplicate population sequencing (TPS) with reflex of specimens with an R5 result to ultradeep sequencing (UDS). TPS g2p5.75 and g2p10: TPS with geno2pheno FPR cutoffs of 5.75% and 10% respectively. TPS (pssm): position specific scoring matrix for X4R5 with a cutoff of −4.75. TPS (g+p): predictions based on combined g2p5.75 and PSSM_x4r5_.

The median change in log_10_ plasma viral load (pVL) from baseline to study weeks 8 and 24 was determined as a function of tropism status as classified by TF-ES, UDS, or the Reflex Test. Median pVL declines for R5 and non-R5 respectively were virtually identical whether subjects were classified by TF-ES or the Reflex Test; at week 8, subjects classified as having non-R5 virus by UDS had slightly smaller changes in pVL than did those classified with TF-ES or the Reflex test (Figure 1A), but this difference did not persist at week 24 (Figure 1B). Regardless of the tropism test used, subjects classified as having R5 virus had significantly greater pVL changes (p<0.0001) than did those with non-R5 virus at week 8 (Figure 1 A) and week 24 (Figure 1 B).

We further assessed the performance of a TPS tropism assay that does not include additional testing by UDS. The performance of both the geno2pheno bioinformatic algorithm [20] and the PSSM algorithm [19] used alone or in combination, was evaluated at week 8. pVL changes between R5 and non-R5 by TPS screening alone were smaller than those recorded for the TF-ES assay or the UDS and Reflex Test. Moreover, the pVL differences between the R5 and non-R5 groups were not statistically significant except when the geno2pheno algorithm with a 10% cutoff (p = 0.04) (Figure 1A). Nevertheless, all TPS methods showed statistically significant differences in pVL between the R5 and non-R5 groups at week 24, however the differences were again smaller than those recorded for TF-ES, UDS or the Reflex Test (Figure 1B).

### Tropism Assay PPV and NPV

**Figure 2:**
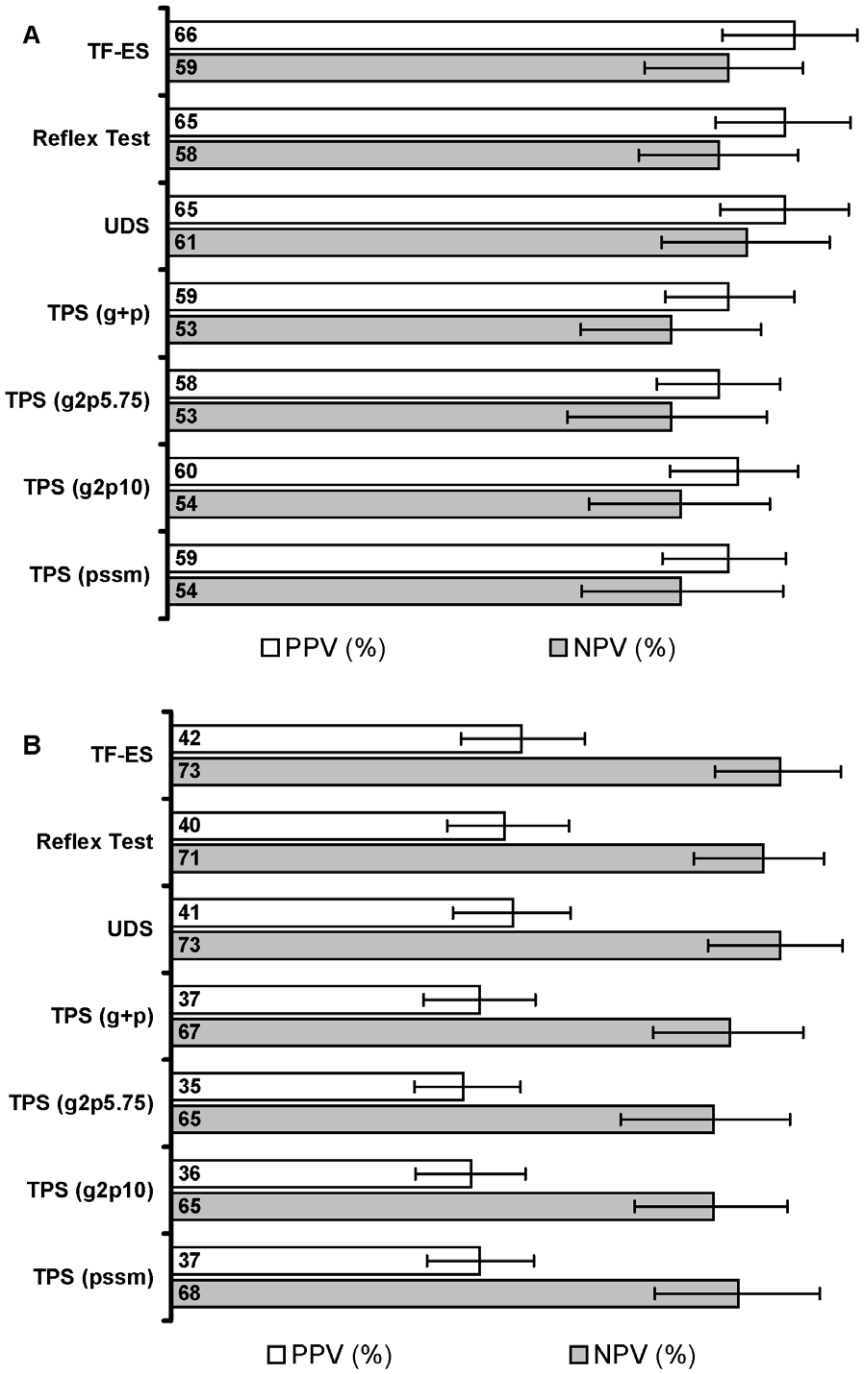
Positive and negative predictive values as a function of tropism assay. **A**: Week 8 results. **B**: Week 24 results. A virologic response was defined as a pVL measurement of <50 copies/mL (weeks 8 and 24) or a log_10_ pVL decline of >2 logs at week 8. PPV: percent of R5 subjects who achieved a virologic response. NPV: percent of non-R5 subjects who did not achieve a virologic response. Error bars represent the 95% CI. TF-ES: Trofile Enhanced Sensitivity (Monogram Biosciences); Reflex Test: triplicate population sequencing (TPS) with reflex of specimens with an R5 result to ultradeep sequencing (UDS). TPS g2p5.75 and g2p10: TPS with geno2pheno FPR cutoffs of 5.75% and 10% respectively. TPS (pssm): position specific scoring matrix for X4R5 with a cutoff of −4.75. TPS (g+p): predictions based on combined g2p5.75 and PSSM_x4r5_.

The positive predictive value (PPV) of each assay defined as the proportion of R5 subjects who achieved a virologic response. Negative predictive value (NPV) was defined as the probability of a non-response to maraviroc in non-R5 subjects. At week 8, PPV was similar for the TF-ES (66%; 95% CI: 58%, 73%) and the Reflex Test (65%; 95% CI: 58%, 72%) (Figure 2A). The NPVs of the two assays were nearly identical as well: 59% (95% CI: 50%, 67%) for TF-ES and 58% (95% CI: 50%, 66%) for the Reflex Test (Figure 2A). At week 24, when patients only achieving a viral load of <50 copies/mL were classified as responders, the PPVs for both assays were lower (42% and 40%) but the NPVs were very high (73%, 71%), indicating that patients with a non-R5 tropism result were unlikely to achieve a virologic response (Figure 2B). When only the TPS tropism predictions were considered, PPVs and NPVs were lower at both weeks 8 and 24 compared to either the Reflex Test, UDS and TF-ES (Figures 2A, 2B).

### Immunologic Response as a Function of Assay and Tropism Status

At week 24, the median CD4+ T-cell count was higher for subjects with R5 virus compared to those with non-R5 virus by all tropism assay methods (Table 2). The gain in CD4+ cells from baseline for the TF-ES R5 group (median = 88 cells/uL; IQR: 26, 162) vs non-R5 (median = 48.3 cells/uL; IQR: 3, 94) was statistically different (p<0.0001), and similarly, for the R5 (median = 88.5 cells/uL, IQR: 41, 163) vs non-R5 (median = 35.5 cells/uL; IQR: 3, 93) groups classified by the reflex test (p<0.0001) (Table 2). The CD4(+) cell changes from baseline for TPS were still significantly different between the R5 and non-R5 groups, however both the changes and the median cell count for the R5 group appeared to be lower and the PSSM method showed the smallest difference between R5 and non-R5 groups (Table 2) suggesting that the PSSM algorithm at the cutoff used, had lower discriminating ability.

**Table 2:** Immunologic response at week 24 as a function of tropism status (N = 325).

		Median CD4+ cells/uL (IQR)^1^		
Assay^2^	Measure	R5	non-R5	P-value^3^
TF-ES	Cells	263 (145,388)	105 (35,210)	
	Change	+88.0 (26,162)	+48.3 (3,94)	<0.0001
Reflex Test	Cells	249 (138,389)	104 (34,212)	
	Change	+88.5 (41,163)	+35.5 (3,93)	<0.0001
UDS	Cells	245 (138,386)	92.5 (32,192)	
	Change	+90.0 (41,162)	+29.3 (3,83)	<0.0001
TPS (g+p):	Cells	224 (118,380)	106 (34,221)	
	Change	+82.0 (27,152)	+35.5 (3,93)	<0.0001
TPS g2p_5.75_:	Cells	213 (106,362)	98 (33,215)	
	Change	+82.0 (24,151)	+31.0 (3,86)	<0.0001
TPS g2p_10_:	Cells	223 (118,367)	104 (35,229)	
	Change	+82.5 (26,152)	+35.0 (3,100)	<0.0001
TPS PSSM:	Cells	212 (95,349)	109 (37,227)	
	Change	+77.0 (23,140)	44.3 (3,103)	0.005

1 Median CD4+ T-cell count at week 24 and change from baseline and inter-quartile range are shown. For subjects who did not complete 24 weeks of the MOTIVATE or A4001029 trial, the last observation is carried forward.

2 TF-ES: Trofile Enhanced Sensitivity (Monogram Biosciences); Reflex Test: triplicate population sequencing (TPS) with reflex of specimens with an R5 result to ultradeep sequencing (UDS). TPS g2p_5.75_ and g2p_10_: TPS with geno2pheno FPR cutoffs of 5.75% and 10% respectively. TPS (pssm): position specific scoring matrix for X4R5 with a cutoff of −4.75. TPS (g+p): predictions based on combined g2p_5.75_ and PSSM_x4r5_.

3 P-values were determined using a two-sided Wilcoxon test.

### Concordance Between Tropism Assays

Concordance and agreement between TPS and UDS methods was high (N = 348; 86.8% concordance, kappa = 0.72). To increase the detection of non-R5 virus by TPS the geno2pheno bioinformatic algorithm [20] and the PSSM_x4r5_ algorithm [19] were used in combination, whereby an X4 prediction by either algorithm classified samples as non-R5. The concordance between geno2pheno and PSSM_x4r5_ was 85.3% (N = 348; kappa = 0.64), similar to the previously reported 88% concordance between these two algorithms [31]. Using UDS as a comparator, TPS had and overall sensitivity of 77.5% (95% CI: 70%, 84%) for non-R5 virus and a specificity of 92.9% (95% CI: 89%, 96%). UDS classified more samples as X4 (39.7%) than TPS (35.1%). Overall concordance and agreement with TF-ES (N = 327) was 81% (kappa = 0.61) for the Reflex Test, 83% (kappa = 0.64) for UDS and 76% (kappa = 0.49) for TPS. For the Reflex Test and TF-ES, 43.1% and 43.7% of the samples, respectively, were classified as non-R5. However, both assays agreed on a non-R5 classification for only 64% of the samples classified as non-R5 by either assay.

### TPS and UDS Discordance Analysis

As shown in Table 3, UDS detected only very low levels of non-R5 (0.13%; 95% CI: 0.09%, 0.17%) in samples with concordant TPS and UDS results (R5/R5 group). In contrast, in samples with concordant non-R5 results, non-R5 variants constituted 51% (95% CI: 44%–57%) of the viral population (X4/X4 group). For the 28 samples classified as non-R5 by UDS but not by TPS, the mean proportion of non-R5 variants by UDS was 10% (95% CI: 6.3%, 14%). These subjects had a poor virologic response at weeks 8 and 24 similar to non-R5 concordant subjects (Table 3). Most of these samples (23/28) were also classified as non-R5 by TF-ES. These results suggest that UDS would have properly excluded the majority of these subjects from maraviroc treatment had this method been used for screening. Fifteen subjects were classified as non-R5 by TPS but R5 by UDS. TF-ES classified 10 of these subjects as R5, and the virologic responses for the non-R5 concordant group were similar to those of R5-concordant subjects (Table 3, X4/R5 group) suggesting that some subjects in this group may have been misclassified as non-R5 by TPS. The PSSM algorithm accounted for 13/15 non-R5s in the X4/R5 group, whereas geno2pheno classified these subjects as R5. Exclusion of PSSM from the TPS analysis would have classifed these subjects as R5 in agreement with the virologic and immunologic responses. Although a further 11 subjects were classified as non-R5 by PSSM but not by geno2pheno in the TPS assay, exclusion of the PSSM algorithm would not have resulted in a different tropism assignment in the Reflex Test as these eleven samples were classified as non-R5 by UDS.

**Table 3:** Virologic and immunologic responses as a function of concordance between TPS and UDS.

	Tropism Result (TPS/UDS)^1^			
	R5/R5	R5/X4	X4/R5	X4/X4
N	186	28	15	98
UDS mean %X4 (95% CI)^2^	0.13 (0.09,0.17)	10 (6.3,14)	0.46 (0.18,0.74)	51 (44,57)
TF-ES %X4 (n/N)^3^	17% (32/186)	82% (23/28)	33% (5/15)	85% (83/98)
Week 8 median pVL (IQR)^4^	−2.4 (−2.9,−1.5) (N = 174)	−0.5 (−1.6,0.0) (N = 28)	−2.7 (−3.0,−1.1) (N = 15)	−1.3 (−2.7,−0.3) (N = 94)
Week 24 median pVL (IQR)^4^	−2.3 (−3.1,−0.8) (N = 184)	−0.7 (−1.6,−0.2) (N = 28)	−2.5 (−3.2,−1.3) (N = 15)	−0.9 (−2.7,−0.3) (N = 98)
Week 24 CD4+ cells (IQR)	249 (138,389) (N = 184)	96.5 (34,190) (N = 28)	240 (134,311) (N = 15)	92 (32,192) (N = 98)
Week 24 CD4+ change (IQR)	+88.5 (41,163) (N = 184)	+39.5 (4,80) (N = 28)	+112.5 (39,160) (N = 15)	+28.3 (2,83) (N = 98)

1 Tropism results were stratified according to concordance between triplicate population sequencing (TPS) and ultradeep sequencing (UDS) R5 and non-R5 (X4) tropism assignments.

2 The mean and 95% confidence interval for the percent of UDS reads classified as X4 in each group. Specimens with ≥2% X4 were classified as non-R5.

3 Percent of Trofile™ Enhanced Sensitivity results classified as non-R5 (D/M or X4).

4 Only viral load changes for subjects who completed 8 weeks of the MOTIVATE or A4001029 trial are shown for the week 8 time point. For subjects who did not complete 24 weeks of the MOTIVATE or A4001029 trial, the last observation is carried forward.

### Limits of Detection (LOD) for non-R5 virus by TPS and UDS

The technical sensitivity of the UDS platform from Roche/454 Life Sciences to detect minority X4 species in an R5 background is approximately 0.5% as determined by testing mixed PCR amplicons rather than mimicked clinical samples (data not shown), in agreement with previous reports [32]. The median number of accepted V3 loop UDS reads per sample on the GS Junior instrument were 1,174 (IQR: 712, 1,588) for the forward sequencing primer and 961 (IQR: 644, 1,418) for the reverse sequencing primer. This level of coverage should enable a detection threshold of 0.5% to 1% for minority variants [28].

We used mimicked clinical samples to assess the sensitivity of TPS and UDS to detect minority X4 (HIV-1 isolate BK132) variants in an R5 (HIV-1 isolate US1) background (Table 4). Because the sensitivity of amplification-based assays for minor viral species depends on the total viral load as well as the proportion of minor species [33], we performed LOD experiments at both 25,000 copies/mL (TPS, UDS) and 100,000 copies/mL (UDS only). At a total viral load of 25,000 copies/mL, the LOD_95_ (LOD at which 95% of samples tested had detectable X4 variants) for minority X4 variants was 20% for TPS and 12% for UDS. When the total viral load was increased to 100,000 copies/mL, the LOD_95_ for UDS was 5%. Overall, the increased sensitivity of the UDS assay relative to TPS for X4 variants in mixtures was consistent with our findings that the UDS provided better separation between maraviroc responders and non-responders (Figures 1 and 2 and Table 2).

**Table 4:** Sensitivity of TPS and UDS for minority X4 species in mimicked clinical samples.

Sample^1^		Assay^2^^,^ 3	
Total virus	X4 Level	TPS	UDS^4^
2.5×10^4^ cp/mL	20%	20/21 (95%)	ND
2.5×10^4^ cp/mL	15%	19/21 (90%)	ND
2.5×10^4^ cp/mL	10%	18/21 (86%)	19/21 (90%)
2.5×10^4^ cp/mL	5%	ND	15/21 (71%)
2.5×10^4^ cp/mL	2%	ND	11/21 (52%)
1×10^5^ cp/mL	5%	ND	20/21 (95%)
1×10^5^ cp/mL	2%	ND	17/21 (81%)

1 R5 (US1) and X4 (BK132) mixtures were diluted in Basematrix 53 to the indicated total viral load and percent of X4 virus.

2 The number and percent of replicates that were scored as X4 by triplicate population sequencing (TPS) and ultradeep sequencing were tabulated.

3 ND: not done.

4 The technical sensitivity of the Roche/454 platform was 0.5%.

### UDS Error Rate

Sequencing errors, particularly insertions and deletion errors in homopolymeric regions, have been reported to constitute a signficant problem for ultradeep pyrosequencing platforms [28], [34], [35]. The substitution, insertion and deletion error rates for UDS of a cloned V3 loop (pNL4-3) were determined with both the GS JR and the GS-FLX instruments (Supporting Information S1). The total error rate was 0.0058–0.0071 miscalls per base; substitutions: 0.0021 miscalls/base; insertions: 0.0025 to 0.0034 miscalls per base; deletions: 0.0012–0.0015 miscalls per base. These error rates are similar to previously published error rates obtained using the 454 GS-20 instrument (Supporting Information S1) [28].

## Discussion

In this work, we have presented an analysis of a genotypic reflex strategy for tropism testing. Although the study was retrospective in nature, the inclusion of A4001029 subjects and MOTIVATE subjects recruited before the closure of enrollment for the A4001029 study resulted in the selection of a population that received maraviroc without regard to their tropism status. This strategy may have reduced possible bias introduced by retrospectively selecting subjects based on their original Trofile tropism results.

Ultradeep sequencing of the HIV-1 envelope V3 loop increased sensitivity for the detection of minority non-R5 variants compared to TPS. UDS detected non-R5 virus in 39.7% of the study samples compared to 35.1% by TPS. The average proportion of non-R5 reads by UDS in UDS non-R5, TPS R5 samples was 10%, which is nominally below the LOD_95_ of the TPS assay for detecting minority non-R5 variants. The virologic and immunologic responses of this group (UDS non-R5/TPS R5) were inferior to those of the R5-only group and similar to those of subjects with non-R5 results by both assays, in agreement with the UDS classification. The value of a more sensitive tropism assay to detect minority non-R5 variants was in retrospective reanalyses of four clinical studies in which samples previously tested with the original Trofile assay were retested with the TF-ES assay or UDS [36]. Reanalysis of the MERIT trial with TF-ES [10], [36] or with UDS [30] showed that either method was able to reclassify as non-R5 a significant number of study subjects originally screened as R5 and the non-inferiority criteria for maraviroc vs. efavirenz defined for this study was then achieved. Our data showed that the virologic and immunologic responses for subjects harboring R5 virus were significantly better than those of non-R5 subjects. Both the Reflex Test and UDS alone demonstrated virtually equal ability to separate responders from non-responders compared to the TF-ES assay. The PPV and NPV of the genotypic Reflex Test were also virtually the same as those of the phenotypic TF-ES assay, at both 8 and 24 weeks, indicating that subjects with a non-R5 tropism result reported by either assay would be unlikely to respond to maraviroc and R5 subjects would be more likely to be responders.

A population sequencing tropism test has been compared to the less-sensitive original Trofile assay used for screening in the MOTIVATE and A4001029 studies, and demonstrated comparable ability to predict virologic response [21]. In the current study using the more sensitive TF-ES assay as a comparator, TPS exhibited poorer accuracy for predicting non-responders. Moreover, the differences in viral load decline between R5 and non-R5 subjects were greater with the TF-ES assay than with the TPS assay. Concordance between tropism predictions of the geno2pheno and the PSSM algorithm was very high, but the PSSM algorithm was more likely to have R5 results classified as non-R5 by UDS. Omitting the PSSM algorithm from the Reflex Test would not have resulted in the misclassification of non-R5 samples as R5 because non-R5 samples would have been correctly classified by UDS. However it is important to emphasize that tropism testing strategies that rely only on population sequencing would still benefit from the added sensitivity of combining geno2pheno and PSSM. Eleven samples that were classified as R5 by geno2pheno using the 5.75% cutoff were classified as non-R5 by PSSM, in agreement with the UDS results. Likewise, 22 non-R5 samples would have been misclassified as R5 by the PSSM algorithm without using geno2pheno. Alternatively, the geno2pheno algorithm with a cutoff of 10% (as recommended by the European tropism testing guidelines [37]) may be considered, as it was 92% concordant with the combined geno2pheno 5.75% cutoff and PSSM tropism predictions. Both methods classified 35% of the samples as non-R5 compared to 28%–29% for geno2pheno at the 5.75% cutoff or PSSM used separately.

The sensitivity of a tropism test, or any amplification-based test, to detect minority viral variants depends on the efficiency of the extraction and amplification methodology to sample the targeted minor species. The technical sensitivity for a given assay detection system may be 0.5% or lower and can be established by clonal analyses [16], [32]. However, the biological sensitivity of the assay system may not be the same, and depends on the viral load of the samples tested as shown here and elsewhere [33]. The eligibility criteria for the MOTIVATE and A4001029 studies required patients to have a viral load of at least 5,000 copies/mL; In the present study, subjects from these trials had a median baseline viral load of approximately 5 log_10_ copies/mL.. Experiments using mimicked clinical samples indicated that the LOD_95_ is approximately 5% at this median pVL. In routine clinical practice however, patient samples with significantly lower viral loads may be provided for tropism testing and the sensitivity for minority X4 man not be as great, for either genotypic or phenotypic tropism assays.

The clinical impact of potentially reduced sensitivity for minority non-R5 variants at lower viral loads requires further investigation. However, in one study it was found that a UDS threshold of 2% X4 and an absolute X4 pVL of 3.7 log_10_ copies/mL were equally predictive of maraviroc response (Predicting maraviroc responses according to number or percentage of X4-using virus among treatment-experienced patients. Heera J, Harrigan PR, Lewis M, Chapman D, Biswas P, Swenson L, Portsmouth S and Valdez H. 18th Conference on Retroviruses and Opportunistic Infections, Feb. 27–March 2 2011, Boston, MA. Abstract 593). Therefore, it is possible that the potential reduction in sensitivity for minority X4 virus in patients at low viral loads may not negatively impact clinical outcome.

This study had a number of limitations. First, there may be envelope gene determinants of tropism outside of the V3 loop that may not be detected by a genotypic test [38], [39]. However, the similarity in predictive values for this genotypic approach compared to TF-ES suggests that such non-V3 loop determinants were not common in this population.

Second, only treatment-experienced subjects were evaluated and the median viral load of the study subjects was relatively high. However, reanalysis of the MERIT study with UDS demonstrated that this technology also effectively discriminates between R5 and non-R5 variants in the treatment-naïve population [30]. A similar study of 312 subjects was conducted by the University of British Columbia Centre for Excellence in HIV/AIDS (Relative Performance of ESTA, Trofile, 454 Deep Sequencing, and “Reflex” Testing for HIV Tropism in the MOTIVATE Screening Population of Therapy-experienced Patients. Brumme C, Wilkin T, Su Z, Schapiro J, Kagan R, Chapman D, Heera J, Valdez H, and Harrigan R. 18th Conference on Retroviruses and Opportunistic Infections, Feb. 27–March 2 2011, Boston, MA. Abstract 666). This study also found that the Reflex Test and UDS had the same discriminating ability as the TF-ES assay for separating maraviroc responders and non-responders. The agreement between our data and this independent study demonstrates the reproducibility of the UDS platform for tropism analysis.

A third limitation is that ultradeep pyrosequencing methods are prone to a number of errors, including a higher insertion and deletion error rate in homopolymeric regions [28], [34], [35]; PCR-mediated recombination that can disrupt haplotypes; sequence resampling; and substitution errors [40]. Two features of our UDS pipeline served to reduce the likelihood of such errors affecting tropism predictions. First, we achieved a high level of redundant coverage which allowed us to discard reads that contained insertion and deletion errors as evidenced by reading frame shifts. Second, as described in Methods, we also imposed an alignment score cutoff to further filter out UDS reads that may have resulted from sequencing errors. The experimentally determined error rate for a control sequence was also found to be significantly below the 2% non-R5 cutoff used in this assay, and therefore was not likely to have affected tropism assignments. The effects of PCR-mediated recombination and resampling may potentially be studied through the use of a novel Primer ID method which utilizes a random tag incorporated into the reverse transcription primer [40]. This method has not yet been investigated for use in UDS-based tropism testing and further studies are needed to evaluate its potential impact on clinical accuracy in this setting.

A fourth limitation of this study is that bioinformatic algorithms for tropism prediction have been trained primarily on subtype B virus and at least one report has shown these to have a lower sensitivity for non-R5 virus in non-B subtypes in at least one study [41]. In the current study 93% of subjects harbored subtype B virus, potentially skewing our conclusions in favor of this subtype. Although the vast majority of HIV-1 infections in the United States are subtype B, subtype C accounts for nearly 50% of HIV infections worldwide [42]. A recent study of tropism prediction algorithms in subtype C infections found good correlation between genotypic methods and a phenotypic tropism assay, achieving a global concordance of 88.6% for the geno2pheno algorithm [43]. In the reanalysis of the MERIT study, in which 40% of the subjects tested harbored non-B virus, UDS and TF-ES had similar performance for predicting virologic outcome in non-subtype B-infected subjects treated with maraviroc [30]. Therefore, genotypic tropism testing is, in most cases, appropriate for patients harboring non-subtype B virus. Future improvements to prediction algorithms for some less common non-B subtypes (for example, subtype D and subtype CRF02_AG) may be warranted [44], [45] and additional data are needed for many rare subtypes.

CCR5 antagonists provide superior virologic and immunologic benefits in patients who harbor exclusively CCR5-using virus [14], [15], [46]. Any tropism screening assay, however, is likely to result in both false-positive and false-negative predictions. While false-positive predictions could exclude eligible patients from CCR5 antagonist therapy, false-negative predictions could lead to patients harboring non-R5 virus receiving CCR5 antagonists and delay the institution of a more effective antiretroviral regimen. However, CCR5 antagonists appear to cause no apparent adverse immunologic affects in patients harboring non-R5 virus. In fact, CD4(+) cell counts showed modest increases in these patients [13], [14], [15]. and. Thus, tropism screening assays may be safely used to select patients for CCR5 antagonist administration.

The relative merits of phenotypic versus genotypic tropism testing have been compared in a recent review [47]. As noted, commercial phenotypic tropism testing is expensive, has a relatively long turnaround times of several weeks, and is only available from one centralized lab using proprietary technology. Genotypic tropism tests may be offered at a significantly lower cost, can be performed more rapidly and offer greater accessibility through the use portable platforms already found in many laboratories that perform genotypic testing. Indeed, genotypic tropism testing is widely used by European laboratories and is recommended by the European tropism testing guidelines [37]. There are additional considerations for deploying UDS platforms for genotypic tropism testing. Although the cost of such platforms and reagents has declined over time, it remains substantial. A single run on a UDS instrument may cost upwards of $1,000. However, the use of molecular identifier tags (MIDs) allowing for the pooling of multiple samples per run can greatly reduce the cost per sample to well below $100. The added complexity of the informatics systems required to manage the large amounts of data generated by UDS, deconvolute pooled data and implement adequate data quality control systems must also be considered. Nevertheless, larger laboratories with experienced personnel, who are able to marshal these resources and regularly perform batched runs of multiple samples can offer a cost effective tropism assay with a turnaround time of approximately 10 days which is only marginally greater than standard genotypic testing.

In conclusion, we have shown that a genotypic tropism assay that utilizes TPS with further testing of R5 samples by UDS has the same ability as the phenotypic TF-ES tropism assay to separate maraviroc responders from non-responders in a treatment-experienced population. The use of genotypic technology affords the opportunity to provide a tropism result more rapidly and at a lower cost than a phenotypic assay. The use of a reflex approach in a clinical laboratory setting will offer a much more rapid turnaround time for obtaining a tropism result for a significant proportion of patients who harbor a non-R5 virus present at levels detectable by population sequencing without the need for reflex testing.

## Materials and Methods

### Ethics Statement

The MOTIVATE 1 and 2 and A4001029 protocols were multi-center, multi-investigator studies, approved by the institutional review board or independent ethics committee at each study center (Supporting Information S2, S3 and S4). Written informed consent was obtained from all participants.

### Study Population and Sample Selection

The MOTIVATE 1 and 2 studies were identically designed randomized placebo-controlled studies to evaluate the safety and efficacy of maraviroc added to optimized background therapy in treatment-experienced patients. Entry criteria included an R5 tropism result with the original Trofile™ assay [11]. The A4001029 study was a randomized placebo-controlled phase 2b study to assess the safety and efficacy of maraviroc in treatment-experienced patients with an X4, dual/mixed or non-reportable tropism result using the original Trofile assay [13]. MOTIVATE patients screened as dual/mixed, X4 or tropism not determined were offered enrollment in A4001029. A4001029 completed enrollment when the MOTIVATE studies were about 25% accrued.

Samples were selected only from patients enrolled in the A4001029 or the MOTIVATE study while the A4001029 study was open to accrual. To be eligible for the present study, patients must have received at least one dose of maraviroc during the A4001029 or the MOTIVATE trial and had tropism screening results of R5, D/M or X4 by the original Trofile assay. Patients whose tropism was undetermined or missing at A4001029 or MOTIVATE study screening were not eligible for this study.

There were approximately 125 A4001029 samples and 245 MOTIVATE 1 and 2 samples with sufficient remaining sample volume for tropism reanalysis from patients enrolled in the MOTIVATE study while the A4001029 study was still open. Taken together, this population represents a cohort of patients who received maraviroc as part of an optimized background regimen without regard for coreceptor tropism.

### Phenotypic Tropism Analysis

The Trofile-ES assay (Monogram Biosciences, San Francisco, CA) was performed retrospectively. R5, X4 or D/M tropism results were obtained for a total of 327 samples. X4 and D/M results were classified together as non-R5.

### Triplicate Amplification and Population Sequencing of the V3 Loop

Viral RNA was extracted from 0.5 mL of plasma on a MagNA Pure LC automated extraction system using the Large Volume Total Nucleic Acid Isolation Kit (Roche Diagnostics Corp. Indianapolis, IN). Reverse transcription and first-round PCR was performed with forward and reverse primers SQV3F1 (HXB2 genomic coordinates 6855–6878) and CO602 (HXB2 genomic coordinates 7786–7817) in three independent replicates of 4 uL of extracted viral RNA (total nucleic acid extract), essentially as described in detail elsewhere [48], [49]. A second-round PCR was then performed using primers customized to contain 3′ adapters necessary for UDS, as detailed in the supplemental materials for Swenson et al [49]. Molecular identifier tags (MIDs) were also incorporated into the primers to allow for amplicons from up to 16 patients to be pooled during UDS. We utilized MID1–13 and MID15–17 (Roche/454 Life Sciences Technical Bulletin 005-2009, *Using Multiplex Identifier (MID) Adaptors for the GS FLX Titanium Chemistry - Extended MID Set*). Bidirectional DNA sequencing was performed for all 3 replicates on an ABI 3730XL DNA analyzer (Applied Biosystems, Foster City, CA) using BigDye 3.1 dye terminators with the target-specific portions of the second round PCR2 primers (forward sequencing primer: HXB2 genomic coordinates 7062–7084; reverse sequencing primer: HXB2 genomic coordinates 7350–7373).

### Population Sequence Data Analysis and Tropism Interpretation

DNA sequence chromatograms were base called and assembled in ReCALL software as described previously [48], [49]. Tropism assignment was performed with the geno2pheno typing program (http://coreceptor.bioinf.mpi-inf.mpg.de/) [20] and the PSSM_x4r5_ program (http://fortinbras.us/cgi-bin/fssm/fssm.pl) [50]. A geno2pheno false-positive rate (FPR) of ≤5.75% was considered to indicate non-R5, and sequences with FPR >5.75% were assigned as R5 [29]. For PSSM_x4r5_ interpretation, sequences scoring ≥−4.75 were assigned as non-R5 and those scoring <−4.75 were assigned as R5.

### UDS of V3 loop PCR Amplicons

Up to 16 samples and controls with distinct 10-nt DNA barcodes incorporated into the PCR primers were pooled by combining aliquots of 10 uL of each PCR product. This library was then purified to remove small fragments using Agencourt AMPure XP beads (Agencourt/Beckman Coulter Genomics, Danvers, MA) according to the *Roche Amplicon Library Preparation Method Manual*. The library was quantitated with the Quant-iT PicoGreen dsDNA Kit (Invitrogen, Carlsbad, CA) and a SpectraMax Model M2 Spectrofluorometer (Molecular Devices, Sunnyvale, CA).

An appropriate dilution of the library was then used to prepare 5 million (GS Junior) or 6.8 million (GS FLX) emulsion PCR (emPCR) A and B microbeads at a ratio of 0.6 to 1.0 molecules of library DNA per microbead. emPCR and bead recovery were then carried out according to the *emPCR Amplification Method Manual - Lib-A GS Junior Titanium Series* (Roche/454 Life Sciences, Branford, CT) or the *emPCR Amplification Method Manual - Lib-A MV GS FLX Titanium Series* (Roche/454 Life Sciences, Branford, CT). Bead enrichment was performed according to the Roche/454 Life Sciences application note *Automated GS FLX Titanium emPCR Enrichment using the REM e System on a Hamilton MICROLAB® STARlet Liquid Handler* (http://www.my454.com/my454).

UDS was performed on the Roche/454 Life Sciences GS Junior by loading 500,000 beads onto the single-region pico-titer plate (PTP) according to the protocol in the *Sequencing Method Manual GS Junior Titanium Series* (Roche/454 Life Sciences, Branford, CT) or 500,000 beads per region of a four-region PTP for the GS FLX platform according to the protocol in the *Sequencing Method Manual GS FLX Titanium Series* (Roche/454 Life Sciences, Branford, CT).

### UDS Sequence Data Analysis and Tropism Assignment

The amplicon pipeline was used for both image processing and signal processing (GS Junior system software version 2.5p1 or GS FLX system software version 2.5.3) and then refiltered with optimized amplicon trim filter parameters (doValleyFilterTrimBack = true, vfBadFlowThreshold = 6, vfLastFlowToTest = 480, vfScanAllFlows = false). This process generated median read lengths of 326 nucleotides, including the V3 loop sequence spanning 105 nucleotides, with some minor length variations accounted for by insertions or deletions in the V3 loop.

Reads were sorted by MID, dereplicated using the program BARTAB [51] and then split into separate files for the forward and reverse sequencing primer directions. The read replicate count was stored in the fasta header for each sequence to allow for downstream calculation of the proportion of X4 reads. The reads were trimmed to the V3 loop open reading frame (ORF); we defined the ORF as a 90 to 120 nucleotide span within the amplicon starting and ending with a cysteine codon (TG[T/C]), where the length of the span was a multiple of 3 and stop codons (TAG/TAA/TGA) were absent. Reads that did not meet these criteria were excluded from further analysis.

The translated amino acid sequences, together with their associated MIDs and replicate counts, were stored in a relational database. The filtered V3 ORFs were processed with the Geno2pheno454 (G2p454) preprocessor and then typed with G2p454 (http://g2p-454.bioinf.mpi-inf.mpg.de/index.php) [52]. The G2p454 scores and alignment scores for each were uploaded to the database. Reads with alignment scores of <75 were excluded, as they likely reflected an improper alignment.

V3 ORFs that occurred less than three times were not tabulated and at minimum 400 valid V3 ORFs per sample and 200 reads per forward and reverse direction were required for a tropism assignment. At this coverage, it is theoretically possible to detect a minority variant at the 1% threshold [28]. Reads with an FPR ≤3.5% were classified as X4 and reads with an FPR >3.5% were classified as R5 [29]. Samples found to have ≥2% X4 reads were classified as non-R5, whereas samples with <2% X4 reads were classified as R5 [29].

The UDS standard flowgram format (SFF) data files for samples in this study have been submitted to the NCBI Short Read Archive (http://www.ncbi.nlm.nih.gov/sra/), submission number SRA056112.

### Reflex Testing Algorithm

All study samples were tested by both population sequencing and UDS. We evaluated a simulated “reflex” approach, whereby the net tropism result was considered to be non-R5 if any of the TPS replicates had a non-R5 result. UDS was used to assign tropism only for result only for samples that had an R5 result by TPS.

### LOD Experiments

Mimicked clinical samples consisted of mixtures of the R5 strain US1 (Genbank accession number AY173952) and the X4 strain BK132 (Genbank accession number AY736821). Plasma samples were diluted in Basematrix 53 (Seracare Life Sciences, Milford, MA) to 20%, 15%, 10%, 5% and 2% X4 at a constant viral load of 25,000 copies/mL, and additionally at 5% and 2% X4 at a constant viral load of 100,000 copies/mL. Seven extractions were performed at each level, and triplicate amplifications were carried out to provide 21 replicates per level.

### Outcome Measures

We assessed the ability of each tropism test to predict the median change in log_10_ plasma viral load (pVL) and virologic response as a function of tropism status at weeks 8 and 24. Virologic response was defined as a viral load of <50 copies/mL or >2 log decline in viral load at week 8, or a viral load of <50 copies/mL at week 24. Immunologic response as defined by changes in CD4(+) T-cell count at week 24 was also tabulated and categorized by tropism status.

Missing virologic outcome data were handled as follows: If a measurement at study entry was missing, the measurement at study screening was used. If a patient discontinued the study prior to week 8 (or week 24), or had a missing value for other reasons, the last available measurement prior to week 8 (or week 24) was carried forward. Patients who did not have any virologic or immunologic measurement after study entry were excluded from the analysis of virologic or immunologic outcome.

### Statistical Analysis

Statistical analysis was performed in SAS (version 9.2, Cary, NC, USA). P-values for median viral load and CD4(+) changes were estimated using a two-sided Wilcoxon test. The positive predictive value (PPV) of each assay was defined as the proportion of R5 subjects who achieved a virologic response. Negative predictive value (NPV) was defined as the proportion of non-R5 subjects who did not achieve a virologic response.

## Supporting Information

Supporting Information S1 **Pyrosequencing error rate for pNL4-3 V3 loop.**
(DOC)

Supporting Information S2 **List of investigators and corresponding ethics committees or Institutional review boards for study A4001029.**
(PDF)

Supporting Information S3 **List of investigators and corresponding ethics committees or Institutional review boards for study A4001027 (MOTIVATE-1).**
(PDF)

Supporting Information S4 **List of investigators and corresponding ethics committees or Institutional review boards for study A4001028 (MOTIVATE-2).**
(PDF)
