# Supplementary material for: A Genotypic Test for HIV-1 Tropism Combining Sanger Sequencing with Ultradeep Sequencing Predicts Virologic Response in Treatment-Experienced Patients
Source: PLoS One. 2012 Sep 27;7(9):e46334. doi: 10.1371/journal.pone.0046334 (PMC3459909; doi:10.1371/journal.pone.0046334)
Supplement: Supporting Information S1 — Pyrosequencing error rate for pNL4-3 V3 loop. (DOC) [file pone.0046334.s001.doc]

Supporting Information S1: Pyrosequencing Error rate for pNL4-3 V3 loop^1^

|  |  |  |  | **Substitutions** | | **Insertions** | | **Deletions** | | **Total error** | |
| --- | --- | --- | --- | --- | --- | --- | --- | --- | --- | --- | --- |
| **Instrument** | **Chemistry** | **Reads** | **Bases** | **Errors** | **Error rate** | **Errors** | **Error rate** | **Errors** | **Error rate** | **Errors** | **Error rate** |
| GS FLX Jr | Titanium | 5657 | 610920 | 1267 | 0.0021 | 1535 | 0.0025 | 748 | 0.0012 | 3550 | 0.0058 |
| GS FLX | Titanium | 7772 | 838339 | 1762 | 0.0021 | 2859 | 0.0034 | 1297 | 0.0015 | 5918 | 0.0071 |
| GS-20^2,3^ | GS-20 | 6827 | NG^4^ | NG^4^ | 0.0012 | NG^4^ | 0.0073 | NG^4^ | 0.0016 | NG^4^ | 0.0098 |

^1^ UDS of the V3 amplicon from a pNL4-3 plasmid clone (Genbank accession M19921) was performed. The V3 loop was amplified in triplicate and included the molecular identifier MID13 incorporated into the PCR2 primers to allow for pooling. The amplicon was sequenced as part of a pool of samples run on the GS FLX and the GS Junior platforms. Sequences were aligned to the pNL4-3 reference V3 sequence and disagreements to the reference were mapped and tabulated using the SWAP454 package (<http://www.broadinstitute.org/science/programs/genome-biology/computational-rd/454-help>). The error rate was defined as the total number of miscalls (substitutions, insertions or deletions) summed up over the 108 nt pNL4-3 V3 sequence divided by the total number of bases called.

^2^ Wang et al, Genome Res 2007: 17: 1195-1201.

^3^ Average error rate for four plasmid clones spanning the 99 codon HIV-1 protease and the first 241 codons of the HIV-1 reverse transcriptase.

^4^ NG: Not Given.
