# Supplementary material for: A Genotypic Test for HIV-1 Tropism Combining Sanger Sequencing with Ultradeep Sequencing Predicts Virologic Response in Treatment-Experienced Patients
Source: PLoS One. 2012 Sep 27;7(9):e46334. doi: 10.1371/journal.pone.0046334 (PMC3459909; doi:10.1371/journal.pone.0046334)
Supplement: Supporting Information S2 — List of investigators and corresponding ethics committees or Institutional review boards for study A4001029. (PDF) [file pone.0046334.s002.pdf]

A4 LIST OF INVESTIGATORS AND CORRESPONDING ETHICS COMMITTEES OR INSTITUTIONAL REVIEW BOARDS

Australia

Coordinating Investigators:
<None Entered>

Center	Principal Investigator	Co-Investigator(s)	Sub-Investigator(s)	Address(es)	Institutional Review Board or Ethics Committee Address(es)
1009	Prof. David Cooper		Kathy M. Barnes<br>Prof. Bruce J. Brew<br>Dr. Alexandra Calmy<br>Assoc. Prof. Andrew Carr<br>Dr. Robert J. Fielden<br>Dr. Elizabeth Hamlyn<br>Dr. Kersten Koelsch<br>Dr. Patrick Mallon<br>Prof. Deborah J. E. Marriott<br>Richard J. Norris<br>Dr. Sarah Pett<br>Martina Rafferty<br>Dr. Christopher J. Weatherall<br>Dr. Alan Winston	National Centre in HIV Epidemiology and Clinical Research<br>Level 2, Medical Centre<br>376 Victoria Street<br>Darlinghurst, NSW 2010<br>AUSTRALIA	St. Vincents Hospital<br>Research Ethics Committee<br>Level 15<br>Victoria Street<br>Darlington, NSW 2010<br>AUSTRALIA
1015	Assoc. Prof. Jennifer Frances Hoy		Sally J. Algar<br>Ms. Mellissa J. Bryant<br>Catherine Cherry<br>Teresa Girke<br>Dr. Sophie A. Herbert<br>Ms. Karen L. Hutchinson<br>Dr. Stephen J. Kent<br>Dr. Richard J. Moore<br>Janine J. Roney<br>Craig P. Scholten<br>Alan Street<br>Dr. Steven L. Wesselingh	The Alfred Hospital<br>Infectious Diseases Unit, Clinical Research Section<br>PO Box 315<br>Melbourne, VICTORIA 3004<br>AUSTRALIA	Alfred Hospital Ethics Committee<br>Commercial Road<br>Melbourne, Victoria 3004<br>AUSTRALIA

090177e1801aef8b\1.1\Approved\26-Mar-2007 11:53

Center	Principal Investigator	Co-Investigator(s)	Sub-Investigator(s)	Address(es)	Institutional Review Board or Ethics Committee Address(es)
1017	Dr. Richard J. Moore		Dr. Jonathan S. C. Anderson<br>Paul Brian Cortissos<br>Dr. Ching Thai Lim<br>Kaye E. Lowe<br>Julia S. Pearce<br>Dr. Jeffrey I. Willcox	The Carlton Clinic<br>88 Rathdowne Street<br>Carlton, Victoria 3053<br>AUSTRALIA	Royal Australian College of General Practitioners National Research and Evaluation Ethics Committee<br>1 Palmerston Crescent<br>SOUTH MELBOURNE, VIC 3205<br>AUSTRALIA
1080	Assoc. Prof. Anthony M. Allworth		Natalie Lara Gerns<br>Dr. Mark Denis Kelly<br>Dr. Emma S. McBryde<br>Assoc. Prof. James S. McCarthy<br>Anne Maree Sleat<br>Assoc. Prof. Marion L. Woods<br>Janelle Zillmann	Infectious Diseases Unit<br>6th Floor Joyce Tweddell Building<br>Royal Brisbane Hospital<br>Herston Road<br>Herston, QUEENSLAND 4029<br>AUSTRALIA	Royal Brisbane & Womens Hospital Health ServiceDistrict Office of the Human Research Ethics Committe<br>Herston Road<br>Herston , QLD, Australia 4029<br>AUSTRALIA
1083 *	Prof. Julian Gold		Dr. Derek J. Chan<br>Mr. Robert Cherry<br>Marg Ewing<br>Dr. Virginia L. Furner<br>Mr. Jason Gao<br>Dr. Henry Mackellar Michelmore<br>Rick Osborne<br>Dr. Jeffrey J. Post<br>Mr. Tony Price<br>Jeganathan Sarangapany<br>Dr. Don E. Smith<br>Grant Sweeney	Albion Street Centre<br>150-154 Albion Street<br>Surry Hills, NSW 2010<br>AUSTRALIA	South Eastern Sydney Area Health Service (Eastern Section) Research Ethics Committee<br>Room G71, Edmund Blacket Building<br>Cnr High & Avoca Sts<br>Randwick, NSW 2031<br>AUSTRALIA
1152	Dr. Neil J. Bodsworth		Dr. Linda S. Dayan<br>Sophie E. Dinning<br>Dr. Robert J. Finlayson<br>Wilma P. Goodyear<br>Dr. Catherine M. Pell<br>Dr. Ross S. Price<br>Robyn A. Richardson<br>Dr. Emanuel G. Vlahakis	Taylor Square Private Clinic<br>393 Bourke Street<br>Surry Hills, NSW 2010<br>AUSTRALIA	South Eastern Sydney Area Health Service (Eastern Section) Research Ethics Committee<br>Room G71, Edmund Blacket Building<br>Cnr High & Avoca Sts<br>Randwick, NSW 2031<br>AUSTRALIA

* Did not randomize subjects

090177e1801aef8b\1.1\Approved\26-Mar-2007 11:53

Center	Principal Investigator	Co-Investigator(s)	Sub-Investigator(s)	Address(es)	Institutional Review Board or Ethics Committee Address(es)
1238	Dr. Cassy Workman		Dr. Kristyn Chantal Adolphe<br>Dr. Robert J. Fielden<br>Dr. Mark Denis Kelly<br>Dr. Catriona J. Ooi<br>Vanessa Rees	AIDS Research Initiative<br>48 Little Oxford Street<br>Darlinghurst, NSW 2010<br>AUSTRALIA	South Eastern Sydney Area Health Service (Eastern Section) Research Ethics Committee<br>Cnr High & Avoca Street<br>Randwick, NSW, Australia 2031<br>AUSTRALIA

090177e1801aef8b\1.1\Approved\26-Mar-2007 11:53

090177e1801aef8b\1.1\Approved\26-Mar-2007 11:53

Belgium

Coordinating Investigators:
<None Entered>

Center	Principal Investigator	Co-Investigator(s)	Sub-Investigator(s)	Address(es)	Institutional Review Board or Ethics Committee Address(es)
1007	Dr. Michel Moutschen		Patricia Dellot<br>Dr. Jean Demonty<br>Dr. Frederic Frippiat<br>Dr. Philippe Leonard<br>Dr. Francoise Uurlings	C.H.U. Sart-Tilman<br>Maladies Infectieuses<br>Domaine du Sart-Tilman B35<br>Liege, 4000<br>BELGIUM	Comite Local d'Ethique Hospitalier<br>CHU St Pierre/UMC St Pieter<br>Batiment Direction<br>Rue Haute/Hoogstraat 322<br>Bruxelles, 1000<br>BELGIUM<br><br>Comite d'Ethique de la Faculte de Medecine<br>Universite de Liege<br>Service de Pharmacologie B23<br>Liege, 4000<br>BELGIUM
1101	Dr. Jean Christophe Goffard		Dr. Michel De Cock<br>Dr. Claire Michele Farber<br>Prof. Jean-Paul van Vooren	Hospital Erasme<br>Route de Lennik 808<br>Brussels, 1070<br>BELGIUM	Comite Local d'Ethique Hospitalier<br>CHU St Pierre/UMC St Pieter<br>Batiment Direction<br>Rue Haute/Hoogstraat 322<br>Bruxelles, 1000<br>BELGIUM<br><br>Comite d'Ethique de la Faculte de medecine de l'ULB<br>CP596<br>Route de Lennick 808<br>Bruxelles, 1070<br>BELGIUM

Center	Principal Investigator	Co-Investigator(s)	Sub-Investigator(s)	Address(es)	Institutional Review Board or Ethics Committee Address(es)
1102	Dr. Bernard C. Vandercam		Angeline Henry<br>Dr. Anne Vincent<br>Dr. Jean-Cyr Yombi	Cliniques Universitaires St-Luc<br>Maladies infectieuses<br>Avenue Hippocrate 10<br>Brussels, 1200<br>BELGIUM	Comission d'Ethique Biomedical<br>Hospitalo-Facultaire<br>Prof. Dr. Maloteaux<br>Avenue Hippocrate 55.14<br>Bruxelles, 1200<br>BELGIUM<br><br>Comite Local d'Ethique Hospitalier<br>CHU St Pierre/UMC St Pieter<br>Batiment Direction<br>Rue Haute/Hoogstraat 322<br>Bruxelles, 1000<br>BELGIUM
1209	Prof. Nathan Clumeck		Dr. Stephane De Wit<br>Dr. Kabamba Kabeya<br>Elisabeth O'Doherty	C.H.U. St-Pierre<br>Clinique des Maladies<br>Infectieuses<br>Rue Haute, 322 (PL5)<br>Brussels, 1000<br>BELGIUM	Comite Local d'Ethique Hospitalier<br>CHU St Pierre/UMC St Pieter<br>Batiment Direction<br>Rue Haute/Hoogstraat 322<br>Bruxelles, 1000<br>BELGIUM

090177e1801aef8b\1.1\Approved\26-Mar-2007 11:53

090177e1801aef8b\1.1\Approved\26-Mar-2007 11:53

Canada

Coordinating Investigators:
<None Entered>

Center	Principal Investigator	Co-Investigator(s)	Sub-Investigator(s)	Address(es)	Institutional Review Board or Ethics Committee Address(es)
1125	Dr. Kevin A. Gough		Dr. Gordon Arbess<br>Dr. Chris Cavacuiti<br>Dr. Brian Cornelson<br>Dr. Ignatius Fong<br>Dr. Abbas Ghavam-Rassoul<br>Dr. Mona Loutfy<br>Dr. Mario Ostrowski	St. Michael's Hospital<br>4 Cardinal Carter Wing North<br>30 Bond Street<br>Toronto, ON M5B 1W8<br>CANADA<br><br>St. Michael's Hospital<br>Rm. 4179 Cardinal Carter Wing<br>30 Bond Street<br>Toronto, ON M5B 1W8<br>CANADA	St. Michael's Hospital Research Ethics Board<br>Queen Wing 4-052<br>30 Bond Street<br>Toronto, ON M5B 1W8<br>CANADA
1126	Dr. Francois Laplante		Dr. Jean-Guy Baril<br>Dr. Marc-Andre Charron<br>Dr. Pierre Cote<br>Dr. Serge Dufresne<br>Dr. Marie-Suzanne Joyal<br>Dr. Patrice Junod<br>Dr. Bernard Lessard<br>Dr. Yves Parent<br>Dr. Denis Poirier<br>Dr. Elise Sasseville<br>Dr. Annie Talbot<br>Dr. Dominique Tessier	Clinique Medicale du Quartier Latin<br>905, Boulevard, Rene-Levesque<br>Montreal, QC H2L 5B1<br>CANADA	IRB Services<br>Suite 328<br>14845-6 Yonge Street<br>Aurora, ON L4G 6H8<br>CANADA

090177e1801aef8b\1.1\Approved\26-Mar-2007 11:53

Center	Principal Investigator	Co-Investigator(s)	Sub-Investigator(s)	Address(es)	Institutional Review Board or Ethics Committee Address(es)
1131	Dr. Benoit Trottier		Dr. Michel Boissonnault<br>Dr. Louise Charest<br>Dr. Marc-Andre Charron<br>Dr. Harold Dion<br>Dr. Stephane Lavoie<br>Dr. Danielle Legault<br>Dr. Daniele Longpre<br>Dr. Pierre-Jean Maziade<br>Dr. Daniel Murphy<br>Dr. Vinh-Kim Nguyen<br>Dr. Robert O'Brien<br>Dr. Rejean Thomas<br>Dr. Sylvie Vezina	Clinique Medicale L'Actuel<br>1130<br>1001 De Maisonneuve Est<br>Montreal, QC H2L 4P9<br>CANADA	Ethica Clinical Research Inc.<br>Institutional<br>Suite 310<br>1255 Transcanada<br>Dorval (Montreal), QC H9P 2V4<br>CANADA
1133	Dr. Christos Tsoukas		Dr. Joseph Cox<br>Dr. Julian M. Falutz<br>Dr. Andreas Giannakis<br>Dr. Norbert Gilmore<br>Dr. Jason Szabo<br>Dr. Howard Turner	Montreal General Hospital,<br>Immune Deficiency Treatment<br>Centre, McGill University Health<br>Centre<br>Room A5-140<br>1650 Cedar Avenue<br>Montreal, QC H3G 1A4<br>CANADA	Montreal General Hospital<br>Biomedical REB<br>1650 Cedar<br>Montreal, QC H3G 1A4<br>CANADA
1134	Dr. Sharon L. Walmsley		Dr. Wayne Lawrence Gold<br>Dr. Rupert Kaul<br>Dr. Kenneth M. Logue<br>Dr. Jeff Powis<br>Dr. Irving E. Salit	University Health Network /<br>Toronto General Hospital<br>585 University Avenue, 5A-West<br>Toronto, ON M5G 2N2<br>CANADA	University Health Network Research Ethics Board<br>Room 8-18<br>700 University Avenue, 8th Floor South<br>Toronto, ON M5G 1Z5<br>CANADA
1232	Dr. Richard G. Lalonde		Dr. James Allan<br>Dr. Norbert Gilmore<br>Dr. Marina Klein<br>Dr. Roger P. Leblanc<br>Dr. John MacLeod<br>Dr. Martin Potter<br>Dr. Pierre Rene<br>Dr. Jean-Pierre Routy	Montreal Chest Institute<br>3650 St-Urbain #J803<br>Montreal, QC H2X 2P4<br>CANADA	Biomedical D Research Ethics Board / Montreal General Hospital<br>McGill University Health Centre<br>Suite C10-148<br>1650 Cedar<br>Montreal, QC H3G 1A4<br>CANADA

* Did not randomize subjects

Center	Principal Investigator	Co-Investigator(s)	Sub-Investigator(s)	Address(es)	Institutional Review Board or Ethics Committee Address(es)
1234	Prof. Ethan Rubinstein<br>Dr. Stuart J. Rosser<br>(Previous PI)		Dr. Ken Kasper<br>Dr. Evelyn W. Lo	St. Boniface General Hospital<br>Section of Infectous Diseases<br>Room N4 -047<br>409 Tachi Avenue<br>Winnipeg, MB R2H 2A6<br>CANADA	Biomedical Research Ethics Board<br>Bannatyne Campus<br>P126 Pathology Building<br>770 Bannatyne Avenue<br>Winnipeg, MB R3E OW3<br>CANADA

090177e1801aef8b\1.1\Approved\26-Mar-2007 11:53

* Did not randomize subjects

Germany

Coordinating Investigators:
<None Entered>

Center	Principal Investigator	Co-Investigator(s)	Sub-Investigator(s)	Address(es)	Institutional Review Board or Ethics Committee Address(es)
1010	Dr. Lutwinus Weitner		Dr. Axel Adam<br>Dr. Thomas Buhk<br>Dr. Stefan Fenske<br>Dr. Holger J. Gellermann<br>Mr. Hauri Goey<br>Mrs. Susanne H. Heesch<br>Dr. Knud Carl Schewe<br>Dr. Hans-Juergen Stellbrink	IBM Study Centre GmbH<br>Grindelalle 35<br>Hamburg, 20146<br>GERMANY	Ethik-Kommission der Aerztekammer Nordrhein<br>Tersteegenstrasse 9<br>Duesseldorf, 40474<br>GERMANY
1093	Dr. Keikawus Arasteh		Christian Herzmann<br>Daniella Izbicki<br>Bastian Krondorfer<br>Michael Rittweger	EPIMED<br>c/o Vivantes-Auguste-Viktoria-Klinikum<br>Rubensstrasse 125<br>Berlin, 12157<br>GERMANY	Ethik-Kommission der Aerztekammer Nordrhein<br>Tersteegenstrasse 9<br>Duesseldorf, 40474<br>GERMANY
1096	Prof. Dr. Andreas Plettenberg		Nicole Bade<br>Carola Floeter<br>Dr. Katrin Graefe<br>Dr. Stefan Hansen<br>Dr. Thore Lorenzen<br>Dr. Albrecht Stoehr<br>Dr. Stefan Unger<br>Dr. Christian Hoffmann<br>Regina Waldeck	ifi-Institut fuer interdiziplinaere Infektiologie & Immunologie GmbH<br>AK St. Georg, Haus K<br>Lohmuehlenstrasse 5<br>Hamburg, 20099<br>GERMANY	Ethik-Kommission der Aerztekammer Nordrhein<br>Tersteegenstrasse 9<br>Duesseldorf, 40474<br>GERMANY
1099	Dr. Jan van Lunzen<br>Dr. Hans-Juergen Stellbrink (Previous PI)		Dr. Olaf Degen<br>Sofie Elena Enderwitz<br>Claudia Schlesner<br>Ms. Bettina Guttowski<br>Dr. Alexander J. Zoufaly	Universitaetsklinikum Hamburg - Eppendorf<br>Innere Medizin, Medizinische Poliklinik Haus 057<br>Martinistrasse 52<br>Hamburg, 20246<br>GERMANY	Ethik-Kommission der Aerztekammer Nordrhein<br>Tersteegenstrasse 9<br>Duesseldorf, 40474<br>GERMANY

090177e1801aef8b\1.1\Approved\26-Mar-2007 11:53

Center	Principal Investigator	Co-Investigator(s)	Sub-Investigator(s)	Address(es)	Institutional Review Board or Ethics Committee Address(es)
1135	Prof. Dr. med. Gerd Faetkenheuer		Dr. Oliver A. Cornely<br>Dr. Pia Regina Hartmann<br>Dr. Gisela-Elisabeth Kremer<br>Dr. Tim Kuemmerle<br>Dr. Clara Lehmann<br>Eleonore Rund<br>Jan Rybniker<br>Dr. Christoph Wyen	Universitaetsklinik Koeln<br>Klinik I fuer Innere Medizin<br>Joseph-Stelzmann-Str 9<br>Koeln, 50924<br>GERMANY	Ethik-Kommission der<br>Aerztekammer Nordrhein<br>Tersteegenstrasse 9<br>Duesseldorf, 40474<br>GERMANY

* Did not randomize subjects

090177e1801aef8b\1.1\Approved\26-Mar-2007 11:53

090177e1801aef8b\1.1\Approved\26-Mar-2007 11:53

Netherlands

Coordinating Investigators:
Joke Patist
Dr. Aukje Rijkeboer

Center	Principal Investigator	Co-Investigator(s)	Sub-Investigator(s)	Address(es)	Institutional Review Board or Ethics Committee Address(es)
1147	Prof. Ilja Mohandas Hoepelman		Dr. Pauline M. de Puy-Ellerbroek<br>Bert Fledderus<br>Dr. Michael A. Gaytant	University Medical Center Utrecht<br>Dept of Internal Medicine & Infection Diseases, F02 - 126<br>Heidelberglaan 100<br>Utrecht, 3584 CX<br>NETHERLANDS	METC UMCU<br>Heidelberglaan 100<br>3584 CX<br>Utrecht<br>NETHERLANDS

* Did not randomize subjects

090177e1801aef8b\1.1\Approved\26-Mar-2007 11:53

Spain

Coordinating Investigators:
<None Entered>

Center	Principal Investigator	Co-Investigator(s)	Sub-Investigator(s)	Address(es)	Institutional Review Board or Ethics Committee Address(es)
1008	Dr. Bonaventura Clotet Sala		Dr. Anna Bonjoch<br>Juan Carlos Martinez<br>Jose Miranda<br>Isabel Bravo Onraita<br>Jordi Puig Pla<br>Dr. Eugenia Negredo Puigmal	Hospital Universitario Germans Trias i Pujol<br>Infectious Diseases<br>Ctra. Del Canyet, s/n<br>Badalona, Barcelona 08916<br>SPAIN	Hospital Germans Trias i Pujol<br>Comite Etico De Investigacion Clinica<br>Ctra. Canyet, s/n<br>Barcelona, 08916<br>SPAIN
1022 *	Dr. Cristina Sarria		Dr. M. Carmen Martinez Garcia<br>Dr. Jesus Sanz Sanz	Hospital de La Princesa<br>Servicio Enfermedades Infecciosas. VIH.<br>C/ Diego de Leon, 62<br>Madrid, Madrid 28006<br>SPAIN	Comite Etico de Investigacion Clinica<br>Ethics Committee of Clinic Investigation<br>C/ Diego de León, 62<br>Farmacologia Clinica Planta 9<br>Madrid, 28006<br>SPAIN<br><br>Hospital Germans Trias i Pujol<br>Comite Etico De Investigacion Clinica<br>Ctra. Canyet, s/n<br>Barcelona, 08916<br>SPAIN

* Did not randomize subjects

Center	Principal Investigator	Co-Investigator(s)	Sub-Investigator(s)	Address(es)	Institutional Review Board or Ethics Committee Address(es)
1111	Dr. Felix Gutierrez		Dr. Maria del Mar Masia<br>Dr. Enrique Bernal Morell<br>Dr. Sergio Padilla Urrea	Hospital General de Elche<br>Unidad de Infecciosas<br>C/ Partida Huertos y Molinos s/n<br>Elche, Alicante 03202<br>SPAIN	Hospital General Universitario de Elche<br>Ethics Committee of Clinical Investigation<br>Cami de L´Almazara, 11<br>Elche, Alicante 03202<br>SPAIN<br><br>Hospital Germans Trias i Pujol<br>Ethics Committee of Clinic Investigation<br>Ctra. Canyet, s/n<br>Barcelona, 08916<br>SPAIN
1112	Dr. Pere Domingo Pedrol		Dr. Josep Cadafalch Arpa<br>Dr. M. Antonia Sambeat Domenech<br>Dr. Montserrat Fuster<br>Dr. Gracia Mateo Garcia<br>Dr. Mar Gutierrez Macia	Hospital De La Santa Creu i Sant Pau<br>Servicio de Enfermedades Infecciosas. VIH<br>Avda. Sant Antoni Maria Claret 167<br>Barcelona, Barcelona 08025<br>SPAIN	Comite Etico de Invesitacion Clinica<br>Hospital de la Santa Creu i Sant Pau<br>Avda. Antonio Maria Claret, 167<br>Barcelona, 08025<br>SPAIN<br><br>Hospital Germans Trias i Pujol<br>Comite Etico De Investigacion Clinica<br>Ctra. Canyet, s/n<br>Barcelona, 08916<br>SPAIN
1113	Dr. Jose Ramon Arribas		Dr. Juan Julian Gonzalez Garcia<br>Dr. Alicia Lorenzo Hernandez<br>Dr. Maria Luisa Montes<br>Dr. Rosa Maria Munoz de Benito<br>Dr. Jose Maria Pena	Hospital Univ. La Paz<br>Unidad VIH<br>Pº de la Castellana, 261<br>Madrid, Madrid 28046<br>SPAIN	Comite Etico de Investigacion Clinica<br>Hospital La Paz<br>Paseo de la Castellana 261<br>Madrid, 28046<br>SPAIN<br><br>Hospital Germans Trias i Pujol<br>Comite Etico De Investigacion Clinica<br>Ctra. Canyet, s/n<br>Barcelona, 08916<br>SPAIN

090177e1801aef8b\1.1\Approved\26-Mar-2007 11:53

Center	Principal Investigator	Co-Investigator(s)	Sub-Investigator(s)	Address(es)	Institutional Review Board or Ethics Committee Address(es)
1161	Dr. Antonio Rivero		Dr. Angela Camacho Espeso<br>Dr. Julian de La Torre Cisneros<br>Dr. Milagros Garcia Lazaro<br>Dr. Rafael Jurado Jimenez<br>Dr. Jose M. Kindelan<br>Dr. M. Carmen Montero Ponferrada	Hospital Reina Sofia<br>Servicio de Enfermedades Infecciosas<br>Avda. Menendez Pidal, s/n<br>Cordoba, Cordoba 14004<br>SPAIN	Comite Autonomico de Ensayos Clinicos de Andalucia<br>Ethics Committee of Clinic Investigation<br>Consejeria de Salud<br>Avda. de la Innovación, s/n<br>Edificio Arena, 1<br>Sevilla, Sevilla 41020<br>SPAIN<br><br>Comite Etico de Investigacion Clinica<br>Hospital Reina Sofia<br>Avda. Menendez Pidal, s/n<br>Cordoba, 14004<br>SPAIN<br><br>Hospital Germans Trias i Pujol<br>Ethics Committee of Clinical Investigation<br>Edificio Maternal, Planta Baixa<br>Crta. De Canyet, s/n<br>Badalona, Barcelona 08916<br>SPAIN

090177e1801aef8b\1.1\Approved\26-Mar-2007 11:53

090177e1801aef8b\1.1\Approved\26-Mar-2007 11:53

Switzerland

Coordinating Investigators:
<None Entered>

Center	Principal Investigator	Co-Investigator(s)	Sub-Investigator(s)	Address(es)	Institutional Review Board or Ethics Committee Address(es)
1023	Prof. Dr. Milos Opravil		Dr. Milo Emil Huber	Universitätsspital Zürich<br>Abteilung Infektionskrankheiten und Spitalhygiene<br>Rämistrasse 100<br>Zürich, 8091<br>SWITZERLAND	Kantonale Ethik-Kommission (KEK)<br>Spezialisierte Unterkommission Innere Medizin (SPUK)<br>Praesident: Prof. Dr. Renato L. Galeazzi<br>Univeritaetsspital Zuerich<br>Sonneggstrasse12<br>Zurich, 8091<br>SWITZERLAND

090177e1801aef8b\1.1\Approved\26-Mar-2007 11:53

United Kingdom

Coordinating Investigators:
<None Entered>

Center	Principal Investigator	Co-Investigator(s)	Sub-Investigator(s)	Address(es)	Institutional Review Board or Ethics Committee Address(es)
1086	Dr. Martin Fisher		Dr. Kazeem Oladipo Aderogba<br>Dr. Angela C. Bailey<br>Katharine Bond<br>Dr. Duncan R. Churchill<br>Ms. Lisa A. Heald<br>Collins Iwuji<br>Tara E. Maher<br>Dr. Alison J. Mears<br>Dr. Kate Nambiar<br>Dr. Usharani Natarajan<br>Dr. David S. Pao<br>Dr. Nicola L. Perry<br>Samantha K. Pushpakaran-Vimala<br>Dr. Iain C. Reeves<br>Jennifer Whetham	Brighton & Sussex University Hospitals NHS Trust<br>HIV/GUM Research Dept<br>The Elton John Centre<br>Sussex House, 1 Abbey Road<br>BRIGHTON, BN2 1ES<br>UNITED KINGDOM	Brighton & Hove Research Ethics Committee<br>Brighton & Hove Primary Care Trust<br>Sixth Floor, Vantage Point<br>New England Road<br>Brighton, BN1 4GW<br>UNITED KINGDOM<br><br>South East Multi Centre Research Ethics Committee<br>Kent & Medway Health Authority<br>Preston Hall<br>Aylesford<br>Maidtone, Kent ME20 7NJ<br>UNITED KINGDOM
1087	Dr. Clifford L. Leen		Dr. Janet M. Andrews<br>Laura Ellis<br>Kristjan Helgason<br>Michael MacKenzie<br>Dr. Claire McGoldrick<br>Claire Frances McHenry<br>Ms. Sheila M. Morris<br>Dr. Hazel Rae	Western General Infirmary<br>Haematology<br>Crewe Road<br>EDINBURGH, EH4 2XU<br>UNITED KINGDOM	Lothian Research Ethics Committee<br>Deaconess House<br>148 Pleasance<br>Edinburgh, EH8 9RS<br>UNITED KINGDOM<br><br>South East Multi Centre Research Ethics Committee<br>Kent & Medway Health Authority<br>Preston Hall<br>Aylesford<br>Maidtone, Kent ME20 7NJ<br>UNITED KINGDOM

* Did not randomize subjects

Center	Principal Investigator	Co-Investigator(s)	Sub-Investigator(s)	Address(es)	Institutional Review Board or Ethics Committee Address(es)
1150	Prof. Philippa Jane Easterbrook		Dr. Elizabeth Hamlyn<br>Fatimah Karim<br>Kristin Kuldanek<br>Emma L. Macfarlane<br>Dr. Frank A. Post<br>Dr. Jan Welch<br>Dr. Tanya Welz	King's College Hospital<br>Caldecot Centre, Department of HIV<br>15-24 Caldecot Road<br>London, SE5 9RS<br>UNITED KINGDOM	Kings College Hospital Research Ethics Committee<br>Camberwell Building<br>King's College Hospital<br>94 Denmark Hill<br>LONDON, SE5 9RS<br>UNITED KINGDOM<br><br>South East Multi Centre Research Ethics Committee<br>Kent & Medway Health Authority<br>Preston Hall<br>Aylesford<br>Maidtone, Kent ME20 7NJ<br>UNITED KINGDOM
1151	Dr. Mark R. Nelson		Dr. Marta Boffito<br>Nicola Boyle<br>Mr. Carl Fletcher<br>Mr. Christopher J. Higgs<br>Dr. Akil George A. Jackson<br>Lucy Katso<br>Ngaire Latch<br>Dr. Desmond D. Maitland<br>Dr. Graeme J. Moyle<br>Jessica R. Osorio<br>Dr. Alastair J. Teague<br>Dr. Alan Winston	Chelsea & Westminster Hospital (GU)<br>Thomas Macauley Ward, 2nd Floor<br>Fulham Road<br>London, SW10 9TH<br>UNITED KINGDOM	Riverside Research Ethics Committee<br>Room 3E03A<br>3rd Floor East, Charing Cross Hospital<br>Fulham Palace Road<br>London, W6 8RF<br>UNITED KINGDOM<br><br>South East Multi Centre Research Ethics Committee<br>Kent & Medway Health Authority<br>Preston Hall<br>Aylesford<br>Maidtone, Kent ME20 7NJ<br>UNITED KINGDOM

090177e1801aef8b\1.1\Approved\26-Mar-2007 11:53

090177e1801aef8b\1.1\Approved\26-Mar-2007 11:53

United States

Coordinating Investigators:
<None Entered>

Center	Principal Investigator	Co-Investigator(s)	Sub-Investigator(s)	Address(es)	Institutional Review Board or Ethics Committee Address(es)
1027	Dr. Bisher Akil		Ann Johiro<br>Ann Johiro	Health Innovations Research<br>Suite 812<br>9201 Sunset Boulevard<br>Los Angeles, CA 90069<br>UNITED STATES	Schulman Associates Institutional Review Board, Inc.<br>4290 Glendale-Milford Road<br>Cincinnati, OH 45242<br>UNITED STATES
1033	Dr. Paul Joseph Cimoch		Dr. Rosemarie Melchor MD<br>Dr. Charles Michael Walworth<br>Susan Wellborn	Orange County Center for Special Immunology<br>Suite 411<br>11190 Warner Avenue<br>Fountain Valley, CA 92708<br>UNITED STATES	Fountain Valley Regional Hospital Institutional Review Board<br>11190 Warner Ave Ste 306<br>Fountain Valley, CA 92708<br>UNITED STATES
1037	Dr. Charles Frank Farthing		Dr. Arash Alborzi<br>Dr. Laveeza Bhatti<br>Dr. Homayoun Khanlou<br>Dr. Mehri S. McKellar<br>Dr. Juan Carlos Ricaurte<br>Dr. Alen Voskanian<br>Dr. Michele Lynn Babaie<br>Dr. Catherine Chien<br>Dr. Vijayabhaskar Reddy Kandula<br>Dr. Susan Sanchez	AIDS Healthcare Foundation Research Center<br>Suite 200<br>99 North La Cienega Blvd.<br>Beverly Hills, CA 90211<br>UNITED STATES	Schulman Associates IRB, Inc.<br>4290 Glendale-Milford Road<br>Cincinnati, OH 45242<br>UNITED STATES

* Did not randomize subjects

090177e1801aef8b\1.1\Approved\26-Mar-2007 11:53

Center	Principal Investigator	Co-Investigator(s)	Sub-Investigator(s)	Address(es)	Institutional Review Board or Ethics Committee Address(es)
1039	Dr. Stephen Eliot Follansbee		Dr. Walford Jeffrey Fessel	Kaiser Permanente<br>Investigational Drugs Pharmacy (drug shipment)<br>Suite 101<br>4131 Geary Boulevard<br>San Francisco, CA 94118<br>UNITED STATES<br><br>Kaiser Permanente Medical Center<br>Clinical Trials Unit, Suite 219<br>4141 Geary Boulevard<br>San Francisco, CA 94118<br>UNITED STATES	Kaiser Permanente Northern California IRB<br>Kaiser Foundation Research Institute<br>16th Floor<br>1800 Harrison Street<br>Oakland, CA 94612-3431<br>UNITED STATES
1042	Dr. Eliot Warren Godofsky		Michele A. Mays<br>Dr. Tanya Schreibman<br>Dr. James Donald DeMaio Jr.	Bach & Godofsky MD, PA<br>Suite 210<br>8451 Shade Avenue<br>Sarasota, FL 34243<br>UNITED STATES<br><br>University Hepatitis Center at Bach & Godofsky<br>Suite 215<br>2415 University Parkway<br>Sarasota, FL 34243<br>UNITED STATES	Schulman Associates IRB<br>4290 Glendale- Miford Road<br>Cincinnati, OH 45242<br>UNITED STATES
1047	Dr. David Holden Henry III		Dr. Patricia Ann Ford<br>Clara Granda-Cameron<br>Dr. Lee Hartner<br>Dr. Michael Joel Haut<br>Nancy Leahy<br>Dr. Bernard Arthur Mason<br>Dr. David Michael Mintzer<br>Dr. Arthur Petrie Staddon<br>Nancy Zieber	Pennsylvania Oncology Hematology Associates<br>230 West Washington Square, 2nd Floor<br>Philadelphia, PA 19106<br>UNITED STATES	Pennsylvania Hospital Research Review Committee<br>800 Spruce Street<br>Philadelphia, PA 19107<br>UNITED STATES

* Did not randomize subjects

090177e1801aef8b\1.1\Approved\26-Mar-2007 11:53

Center	Principal Investigator	Co-Investigator(s)	Sub-Investigator(s)	Address(es)	Institutional Review Board or Ethics Committee Address(es)
1049	Dr. Frederick A. Cruickshank<br>Dr. Joseph Gregory Jemsek (Previous PI)		Aimee O. Buck<br>Christie Furr Roeske<br>Joel Wesley Thompson	Jemsek Clinic<br>Suite 200<br>14330 Oakhill Park Lane<br>Huntersville, NC 28078<br>UNITED STATES	Schulman Associates Institutional Review Board, Inc.<br>4290 Glendale-Milford Road<br>Cincinnati, OH 45242<br>UNITED STATES
1056	Dr. Robert Anderson Myers Jr.		Brian Arey<br>Dr. Anita Jeanne Culp<br>Dr. John Mark Post<br>Adrianne Kazmier	Phoenix Body Positive<br>Suite 200<br>1144 East McDowell Road<br>Phoenix, AZ 85006<br>UNITED STATES	Schulman Associates IRB, Inc.<br>4290 Glendale-Milford Road<br>Cincinnati, OH 45242<br>UNITED STATES
1058	Dr. Gerald Pierone Jr.		Dr. Andrea Jo Balconis<br>Chandra Devi Kantor ARNP<br>Dorothy Bulgin-Coleman	Treasure Coast Infectious Disease Consultants<br>3715 7th Terrace<br>Vero Beach, FL 32960<br>UNITED STATES	Schulman Associates Institutional Review Board, Incorporated<br>4290 Glendale - Milford Road<br>Cincinnati, OH 45242<br>UNITED STATES
1059	Dr. Jayashree Ravishankar		Dr. Jack Alan DeHovitz<br>Susan Holman<br>Dr. Jesi Ramone<br>Lisa Shipper<br>Ronald W. Harris<br>Mr. Gabriel Larson<br>Dr. Cyril Cinco Llamoso	University Hospital of Brooklyn<br>State University of New York<br>Downstate Medical Center<br>450 Clarkson Avenue<br>Brooklyn, NY 11203<br>UNITED STATES	State University of New York<br>Downstate Medical Center<br>Institutional Review Board<br>Box 129<br>450 Clarkson Avenue<br>Brooklyn, NY 11203<br>UNITED STATES
1062	Dr. William Jay Robbins			Infectious Diseases of Central Florida PA<br>Suite 300<br>1720 South Orange Avenue<br>Orlando, FL 32806<br>UNITED STATES	Schulman Associates IRB, Inc.<br>4290 Glendale-Milford Road<br>Cincinnati, OH 45242<br>UNITED STATES

* Did not randomize subjects

Center	Principal Investigator	Co-Investigator(s)	Sub-Investigator(s)	Address(es)	Institutional Review Board or Ethics Committee Address(es)
1063	Dr. Michael Saag		Dr. Laura Hinkle Bachmann<br>Dr. John Wyatt Gnann Jr.<br>Dr. Paul Andres Goepfert<br>Dr. Sonya L. Heath<br>Dr. Victoria Anne Johnson<br>Dr. John Michael Kilby<br>Dr. Michael James Mugavero<br>Dr. Mukesh Patel<br>Jennifer Peterson<br>Dr. Amy Player<br>James L. Raper<br>Laura P. Secord<br>Dr. Ming Walker<br>Dr. James M. Sizemore	University of Alabama at Birmingham 1917 Clinic Community Care Building / 2nd Floor<br>908 20th Street South<br>Birmingham, AL 35294<br>UNITED STATES	Western Institutional Review Board<br>3535 Seventh Avenue, SW<br>Olympia, WA 98502<br>UNITED STATES
1064	Dr. Kunthavi Sathasivam		Dr. Joseph Baker<br>Tina Celenza<br>Dr. Roxanne D. Cox-Iyamu<br>Luke Johnsen<br>Barbara R. Lewis<br>Dr. Ambalavanapillai Mathivannan<br>Dr. Bryan Baugh<br>Dr. Philippe Albert Chiliade<br>Danbi Mallin	Whitman Walker Clinic<br>1701 14th Street NW<br>Washington, DC 20009<br>UNITED STATES	Whitman-Walker Clinic Institutional Review Board<br>1701 14th St. NW<br>Washington, DC 20009<br>UNITED STATES

090177e1801aef8b\1.1\Approved\26-Mar-2007 11:53

Center	Principal Investigator	Co-Investigator(s)	Sub-Investigator(s)	Address(es)	Institutional Review Board or Ethics Committee Address(es)
1065	Dr. Lawrence E. Schwartz		Dr. Marina Arbuck<br>Dr. Philip Cheney Craven<br>Ann E. Hyder<br>Dr. Elizabeth Anne Lien<br>Dr. Peter K. Marsh<br>Dr. David Winters McEniry<br>Dr. Romana V. Popa	Northwest Medical Specialities, PLLC<br>Infections Limited, PS<br>Suite B<br>220 15th Avenue<br>Puyallup, WA 98372<br>UNITED STATES<br><br>Northwest Medical Specialties, PLLC<br>Suite 405<br>1624 South I Street<br>Tacoma, WA 98405<br>UNITED STATES	Schulman Associates IRB, Inc.<br>4290 Glendale-Milford Road<br>Cincinnati, OH 45242<br>UNITED STATES
1066	Silver Sisneros		Dr. Kathleen A. Clanon<br>Dr. Howard Ellis Edelstein<br>Leslie J. Reynolds<br>Ms. Cynthia Rowden<br>Dr. Beth Schweitzer	Alameda County Medical Center, Adult Immunology Clinic<br>1411 East 31st Street<br>Oakland, CA 94602<br>UNITED STATES	ALAMEDA COUNTY MEDICAL CENTER<br>1411 EAST 31ST STREET<br>OAKLAND , CA 94602<br>UNITED STATES
1067	Dr. Louis Marshall Sloan		Dr. Marc Tribble	North Texas Infectious Diseases Consultants PA<br>Suite 710<br>3409 Worth Street<br>Dallas, TX 75246<br>UNITED STATES	Schulman Associates Institutional Review Board, Inc.<br>4290 Glendale-Milford Road<br>Cincinnati, OH 45242<br>UNITED STATES
1069	Dr. Corklin Ray Steinhart		Dr. Clifford A. Kinder<br>Amy Sue Liebmann<br>Dr. David E. Schmitt<br>Dr. Isabela B. Sierra<br>Dr. Allan John Stein	Steinhart Medical Associates<br>Suite 806<br>Mercy Professional Bldg.<br>3661 S. Miami Ave..<br>Miami, FL 33133<br>UNITED STATES<br><br>Steinhart Medical Associates<br>Suite 810<br>3661 South Miami Avenue<br>Miami, FL 33133<br>UNITED STATES	Schulman Associates Institutional Review Board, Inc.<br>4290 Glendale-Milford Road<br>Cincinnati, OH 45242<br>UNITED STATES

* Did not randomize subjects

090177e1801aef8b\1.1\Approved\26-Mar-2007 11:53

Center	Principal Investigator	Co-Investigator(s)	Sub-Investigator(s)	Address(es)	Institutional Review Board or Ethics Committee Address(es)
1070	Dr. Melanie Ann Thompson		Dacenta A. Grice	AIDS Research Consortium of Atlanta, Inc.<br>Suite 130<br>131 Ponce de Leon Avenue, NE<br>Atlanta, GA 30308<br>UNITED STATES	AIDS Research Consortium of Atlanta Incorporated-Institutional Review Board<br>Suite 130<br>131 Ponce de Leon Avenue, NE<br>Atlanta, GA 30308<br>UNITED STATES
1071	Dr. William James Towner Jr.		Dr. Joseph C. Chang<br>Dr. Hai Linh Kerrigan<br>Dr. Marc J. LaRiviere<br>Dr. John P. Martin<br>Dr. Jim H. Nomura<br>Dr. Tomiko Stein<br>Dr. Kim-Huong Thi Tran<br>Dr. Townson Tsai<br>Karole Velzy<br>Dr. Diane Yamamoto Skowron<br>Dr. Leslie Wang	Kaiser Permanente Medical Center/ Southern CA Permanente Med. Group<br>Infectious Disease/ 2nd Floor<br>1505 North Edgemont Street<br>Los Angeles, CA 90027<br>UNITED STATES	Kaiser Permanente Southern California IRB Research and Evaluation<br>2nd Floor<br>393 East Walnut Street<br>Pasadena, CA 91101<br>UNITED STATES
1073	Dr. David Allen Wheeler		Dr. Mary E. Alder<br>Dr. Sujata H. Ambardar<br>Dr. Allan J. Morrison Jr.<br>Dr. Donald Martin Poretz<br>Dr. Ann I. Rixinger	Infectious Diseases Physicians, Incorporated, Office of Clinical Research<br>Suite 250<br>3289 Woodburn Road<br>Annandale, VA 22003<br>UNITED STATES	Schulman Associates Institutional Review Board, Incorporated<br>4290 Glendale - Milford Road<br>Cincinnati, OH 45242<br>UNITED STATES
1074	Dr. Sally Williams		Dr. David Andrew Bong	The Vancouver Clinic<br>700 Northeast 87th Avenue<br>Vancouver, WA 98664<br>UNITED STATES	Schulman Associates Institutional Review Board, Incorporated<br>4290 Glendale - Milford Road<br>Cincinnati, OH 45242<br>UNITED STATES

* Did not randomize subjects

090177e1801aef8b\1.1\Approved\26-Mar-2007 11:53

090177e1801aef8b\1.1\Approved\26-Mar-2007 11:53

Center	Principal Investigator	Co-Investigator(s)	Sub-Investigator(s)	Address(es)	Institutional Review Board or Ethics Committee Address(es)
1076 *	Dr. Michael Bruce Wohlfeiler		Dr. Joseph R. Piperato<br>Jamie Lopez	Wohlfeiler, Piperato & Associates, LLC<br>Suite 202<br>16401 N.W. 2nd Ave<br>North Miami Beach, FL 33169<br>UNITED STATES	Schulman Associates Institutional Review Board, Inc.<br>4290 Glendale-Milford Road<br>Cincinnati, OH 45242<br>UNITED STATES
1078	Dr. Bienvenido Gamulo Yangco			Infectious Disease Research Institute, Inc.<br>Suite 203<br>4620 North Habana Avenue<br>Tampa, FL 33614<br>UNITED STATES	Schulman Associates IRB<br>4290 Glendale- Miford Road<br>Cincinnati, OH 45242<br>UNITED STATES
1079	Dr. Barry Stephen Zingman		Julie Naczi Sarlo	Montefiore Medical Center<br>AIDS Clinic<br>111 East 210th Street<br>Bronx, NY 10467<br>UNITED STATES	Biomedical Research Alliance of New York, LLC<br>Institutional Review Board<br>Suite 100<br>225 Community Drive<br>Great Neck, NY 11021<br>UNITED STATES
1177	Dr. Robert Key Bolan		Dr. Michael J. Hall<br>Cynthia Harrison	Jeffrey Goodman Special Care Clinic<br>1625 Schrader Boulevard<br>Los Angeles, CA 90028<br>UNITED STATES	Schulman Associates IRB<br>4290 Glendale- Miford Road<br>Cincinnati, OH 45242<br>UNITED STATES
1180	Dr. Edwin DeJesus		Dr. Roberto Ortiz	Orlando Immunology Center<br>1701 North Mills Ave<br>Orlando, FL 32803<br>UNITED STATES	Schulman Associates Institutional Review Board, Inc.<br>4290 Glendale-Milford Road<br>Cincinnati, OH 45242<br>UNITED STATES
1181	Dr. Jerome A. Ernst		Dr. Yuriy S. Akulov<br>Dr. Douglas G. Mendez	ACRIA<br>17th Floor<br>230 West 38th Street<br>New York, NY 10018<br>UNITED STATES	Schulman Associates Institutional Review Board, Incorporated<br>4290 Glendale - Milford Road<br>Cincinnati, OH 45242<br>UNITED STATES

* Did not randomize subjects

090177e1801aef8b\1.1\Approved\26-Mar-2007 11:53

Center	Principal Investigator	Co-Investigator(s)	Sub-Investigator(s)	Address(es)	Institutional Review Board or Ethics Committee Address(es)
1183	Dr. Shawn K. Hassler		Dr. Ivor A. Emanuel<br>Mr. Martin C. Kramer	Benchmark Research<br>Suite 1415<br>490 Post Street<br>San Francisco, CA 94102<br>UNITED STATES<br><br>Benchmark Research<br>Suite 1442<br>490 Post Street<br>San Francisco, CA 94102<br>UNITED STATES<br><br>Office of Shawn K. Hassler, MD<br>Suite 600<br>870 Market Street<br>San Francisco, CA 94102<br>UNITED STATES	Schulman Associates Institutional Review Board, Inc.<br>4290 Glendale-Milford<br>Cincinnati, OH 45242<br>UNITED STATES
1185	Dr. Stephen P. Hauptman			Hauptman Family Health Center<br>Suite 303<br>2000 Hamilton Street<br>Philadelphia, PA 19130<br>UNITED STATES	Schulman Associates IRB<br>4290 Glendale- Miford Road<br>Cincinnati, OH 45242<br>UNITED STATES
1188	Dr. Joseph P. McGowan		Dr. Marcia Ellen Epstein<br>Dr. Bruce Hirsch<br>Dr. David Hirschwerk<br>Dr. Angela Kim<br>Dr. Yelena Markovskaya	North Shore University Hospital<br>4th Floor, Lippert Building<br>300 Community Drive<br>Manhasset, NY 11030<br>UNITED STATES	Biomedical Research Alliance of New York, LLC<br>Suite 100<br>225 Community Drive<br>Great Neck, NY 11021<br>UNITED STATES
1190 *	Dr. George Louis Drusano		Minda Hubbard<br>Dr. Ralph Liporace	Albany Medical College<br>47 New Scotland Avenue<br>Albany, NY 12208<br>UNITED STATES	Western Institutional Review Board<br>3535 7th Avenue, Southwest<br>Olympia, WA 98502-5010<br>UNITED STATES
1191	Dr. Bruce Stephen Rashbaum		Mr. Thomas A. Kantor<br>Dr. Sylvia R. Medley	Capital Medical Associates, P.C.<br>Suite 800<br>1640 Rhode Island Avenue, NW<br>Washington, DC 20036<br>UNITED STATES	Schulman Associates IRB<br>4290 Glendale- Miford Road<br>Cincinnati, OH 45242<br>UNITED STATES

* Did not randomize subjects

090177e1801aef8b\1.1\Approved\26-Mar-2007 11:53

Center	Principal Investigator	Co-Investigator(s)	Sub-Investigator(s)	Address(es)	Institutional Review Board or Ethics Committee Address(es)
1212	Dr. Stephen Lawrence Becker		Mark L. Illeman<br>Ellen K. Opie<br>Dr. Christopher Schiessl<br>Dr. Lorna Marie Thornton	Pacific Horizon Medical Group<br>Ste 512<br>2351 Clay St<br>San Francisco, CA 94115-1931<br>UNITED STATES	Schulman Associates IRB, Inc.<br>4290 Glendale-Milford Road<br>Cincinnati, OH 45242<br>UNITED STATES
1213	Dr. Nicholaos C. Bellos		Dr. Rasha Ghurani<br>Yvonne Michelle Lindahl<br>Durward Watson<br>Dr. Robert Waldrup Henderson III<br>Dr. Mark Joseph Hupert	Southwest Infectious Disease Associates, Nicholaos C. Bellos, M.D., P.A.<br>2909 Lemmon Avenue<br>Dallas, TX 75204<br>UNITED STATES	Schulman Associates Institutional Review Board, Inc.<br>4290 Glendale-Milford Road<br>Cincinnati, OH 45242<br>UNITED STATES
1220	Dr. Trevor N. Hawkins		Dr. Mary Ellen Lawrence<br>Dr. Michael Dane Palestine	Southwest Care Center<br>Suite E<br>649 Harkle Road<br>Santa Fe, NM 87505<br>UNITED STATES	Schulman Associates Institutional Review Board, Incorporated<br>4290 Glendale - Milford Road<br>Cincinnati, OH 45242<br>UNITED STATES
1223	Dr. Daniel Benjamin Klein		Dr. Susan Jane Jacobson	Kaiser Permanente<br>27400 Hesperian Boulevard<br>Hayward, CA 94545<br>UNITED STATES<br><br>Kaiser Permanente<br>Department of Infectious Diseases (Drug Shipment)<br>30116 Eigenbrodt Way<br>Union City, CA 94587<br>UNITED STATES	Kaiser Permanente Northern California IRB<br>Kaiser Foundation Research Institute<br>16th Floor<br>1800 Harrison Street<br>Oakland, CA 94612-3431<br>UNITED STATES

* Did not randomize subjects

090177e1801aef8b\1.1\Approved\26-Mar-2007 11:53

Center	Principal Investigator	Co-Investigator(s)	Sub-Investigator(s)	Address(es)	Institutional Review Board or Ethics Committee Address(es)
1224	Dr. Jason Mark Leider		Dr. Jacobo Abadi<br>Tracey Barnett<br>Dr. Fernando C. Carnavali<br>Mindy A. Golatt<br>Dr. Julie Hoffman<br>Dr. Elizabeth Robin Jenny-Avital<br>Dr. Mindy Jill Katz<br>Maura Porricolo<br>Dr. Michael George Rosenberg	Jacobi Medical Center<br>1400 Pelham Parkway South<br>Bronx, NY 10461<br>UNITED STATES	Biomedical Research Alliance of New York, LLC<br>Suite 100<br>225 Community Drive<br>Great Neck, NY 11021<br>UNITED STATES
1226	Dr. Daniel Skiest<br>Dr. Calvin Jay Cohen (Previous PI)<br>Dr. Claudia Martorell (Previous PI)<br>Dr. Anne Burnett Morris (Previous PI)		Ms. Arlene Bermudez<br>Dr. Amy E. Colson<br>Ms. Susan M. Cournoyer<br>Dr. Carlos Flores<br>Ms. Carol A. Kane<br>Dr. Harry Mark Schrager<br>Ms. Myrna J. Schulte<br>Maribel Torano<br>Ms. Laurie F. Wojtusik<br>Ms. Margarita Canuel<br>Dr. St. John D. McGrath	CRI of New England<br>Suite 30<br>780 Chestnut Street<br>Springfield, MA 01107<br>UNITED STATES	New England IRB<br>40 Washington Street, Ste 130<br>Wellesley, MA 02481<br>UNITED STATES
1229	Dr. Roy Steigbigel		Sandra Brown<br>Dr. Lisa M. Chirch<br>Wayne Patterson<br>Frank Albergo	SUNY at Stony Brook<br>Division of Infectious Diseases<br>Hsc T-15 Room 080<br>Stony Brook, NY 11794<br>UNITED STATES<br><br>University Hospital, State University of New York at Stony Brook<br>Pharmacy Department, LI<br>Room 1-841<br>Stony Brook, NY 11794-7310<br>UNITED STATES	Committee on Research Involving Human Subjects(CORIHS)<br>Research Administration Library 5th Floor<br>State University of New York at Stony Brook<br>Stony Brook, NY 11794-3368<br>UNITED STATES

Center	Principal Investigator	Co-Investigator(s)	Sub-Investigator(s)	Address(es)	Institutional Review Board or Ethics Committee Address(es)
1240	Dr Jorge E. Rodriguez		Dr. R. Derrick Knowles	Orange Coast Medical Group<br>Suite 126<br>361 Hospital Road<br>Newport Beach, CA 92663<br>UNITED STATES	Schulman Associates IRB, Inc.<br>4290 Glendale-Milford Road<br>Cincinnati, OH 45242<br>UNITED STATES
1251	Dr. Alfred F. Burnside Jr.			The Burnside Clinic<br>14 Calendar Court<br>Columbia, SC 29206<br>UNITED STATES	Schulman Associates IRB, Inc.<br>4290 Glendale-Milford Road<br>Cincinnati, OH 45242<br>UNITED STATES

090177e1801aef8b\1.1\Approved\26-Mar-2007 11:53
