# Supplementary material for: A Genotypic Test for HIV-1 Tropism Combining Sanger Sequencing with Ultradeep Sequencing Predicts Virologic Response in Treatment-Experienced Patients
Source: PLoS One. 2012 Sep 27;7(9):e46334. doi: 10.1371/journal.pone.0046334 (PMC3459909; doi:10.1371/journal.pone.0046334)
Supplement: Supporting Information S3 — List of investigators and corresponding ethics committees or Institutional review boards for study A4001027 (MOTIVATE-1). (PDF) [file pone.0046334.s003.pdf]

A4 LIST OF INVESTIGATORS AND CORRESPONDING ETHICS COMMITTEES OR INSTITUTIONAL REVIEW BOARDS

Canada

Coordinating Investigators:

<None Entered>

Center	Principal Investigator	Co-Investigator(s)	Sub-Investigator(s)	Address(es)	Institutional Review Board or Ethics Committee Address(es)
1055	Dr. Jonathan Angel		Dr. D. William Cameron<br>Dr. Curtis Cooper<br>Dr. Gary E. Garber<br>Dr. Stephen Kravcik<br>Dr. Barry Craig Lee<br>Dr. Paul MacPherson<br>Dr. Claire Touchie	Ottawa Hospital, General Campus<br>Module G-12<br>501 Smyth Road<br>Ottawa, ON K1H 8L6<br>CANADA	Ottawa Hospital Research Ethics Board<br>Suite 106<br>751 Parkdale Avenue<br>Ottawa, ON K1Y 1J7<br>CANADA

090177e1801b1594\Approved\Approved On: 24-Sep-2007 20:04

090177e1801b1594\Approved\Approved On: 24-Sep-2007 20:04

Center	Principal Investigator	Co-Investigator(s)	Sub-Investigator(s)	Address(es)	Institutional Review Board or Ethics Committee Address(es)
1056	Dr. Brian Conway		Dr. Stanley De Vlaming<br>Dr. Robert Reynolds	Pender Community Health Centre<br>59 West Pender Street<br>Vancouver, BC V6B 1R3<br>CANADA<br><br>University of British Columbia/ Downtown Infectious Diseases Clinic<br>201 - 1200 Burrand Street<br>Vancouver, BC V6Z 2C7<br>CANADA	University of British Columbia<br>Clinical Research Ethics Board<br>Room 210<br>828 West 10th Avenue<br>Vancouver, BC V5Z 1L8<br>CANADA
1058	Dr. Kevin A. Gough		Dr. Gordon Arbess<br>Dr. Chris Cavacuiti<br>Dr. Brian Cornelson<br>Dr. Ignatius Fong<br>Dr. Abbas Ghavam-Rassoul<br>Dr. Mona Loutfy<br>Dr. Mario Ostrowski	St. Michael's Hospital<br>4 Cardinal Carter Wing North<br>30 Bond Street<br>Toronto, ON M5B 1W8<br>CANADA	St. Michael's Hospital Research Ethics Board<br>Queen Wing 4-052<br>30 Bond Street<br>Toronto, ON M5B 1W8<br>CANADA

090177e1801b1594\Approved\Approved On: 24-Sep-2007 20:04

Center	Principal Investigator	Co-Investigator(s)	Sub-Investigator(s)	Address(es)	Institutional Review Board or Ethics Committee Address(es)
1059	Dr. Francois Laplante		Dr. Jean-Guy Baril<br>Dr. Marc-Andre Charron<br>Dr. Pierre Cote<br>Dr. Serge Dufresne<br>Dr. Marie-Suzanne Joyal<br>Dr. Patrice Junod<br>Dr. Bernard Lessard<br>Dr. Yves Parent<br>Dr. Denis Poirier<br>Dr. Elise Sasseville<br>Dr. Annie Talbot<br>Dr. Dominique Tessier	Clinique Medicale du Quartier Latin<br>905, Boulevard, Rene-Levesque<br>Montreal, QC H2L 5B1<br>CANADA	IRB Services<br>Suite 328<br>14845-6 Yonge Street<br>Aurora, ON L4G 6H8<br>CANADA
1060	Dr. Julio S.G. Montaner		Dr. Marianne Harris<br>Dr. Valentina Montessori<br>Dr. Peter Phillips<br>Dr. Natasha Press	St. Paul's Hospital<br>The John Ruedy Immunodeficiency Clinic<br>5th Floor Burrard<br>1081 Burrard Street<br>Vancouver, BC V6Z 1Y6<br>CANADA	Providence Health Care<br>Office of Research Services (ORS)<br>St Paul's Hospital<br>11th Floor<br>1190 Hornby Street<br>Vancouver, BC V6Z 2K5<br>CANADA

* Did not randomize subjects

Center	Principal Investigator	Co-Investigator(s)	Sub-Investigator(s)	Address(es)	Institutional Review Board or Ethics Committee Address(es)
1061	Dr. Anita R. Rachlis		Dr. Benny Chang	Sunnybrook and Women's College Health Sciences Centre<br>2075 Bayview Avenue<br>Toronto, ON M4N 3M5<br>CANADA	Sunnybrook and Womens College Health Sciences Center<br>Sunnybrook and Womens College Health Sciences Center<br>Room S133<br>2075 Bayview Avenue<br>Toronto, ON M4N 3M5<br>CANADA
1062	Dr. Stephen David Shafran		Dr. Isabelle Chiu<br>Dr. Michelle Foisy<br>Dr. Stan Houston<br>Dr. Christine Hughes<br>Dr. Mark Joffe<br>Dr. Dennis Y. Kunimoto<br>Dr. Lilly Miedzinski<br>Dr. Lynora Saxinger<br>Dr. Geoffrey David Taylor	Royal Alexandra Hospital<br>10240 Kingsway<br>Edmonton, AB T5H 3V9<br>CANADA	Health Research Ethics Board, University of Alberta<br>213 Heritage Medical Research Centre<br>Edmonton, AB T6G 2S2<br>CANADA

* Did not randomize subjects

090177e1801b1594\Approved\Approved On: 24-Sep-2007 20:04

090177e1801b1594\Approved\Approved On: 24-Sep-2007 20:04

Center	Principal Investigator	Co-Investigator(s)	Sub-Investigator(s)	Address(es)	Institutional Review Board or Ethics Committee Address(es)
1063	Dr. Fiona Smaill		Dr. Philippe El-Helou<br>Dr. Shariq Haider<br>Dr. Christine Lee<br>Dr. Marek Smieja<br>Dr. Irwin Walker	HAMILTON HEALTH SCIENCES CORPORATION, MCMASTER SITE<br>Special Immunology Services Clinic<br>Room 2F41<br>1200 MAIN STREET WEST<br>HAMILTON, ON L8N 3Z5<br>CANADA	Hamilton Health Sciences/McMaster University Faculty of Health Sciences Research Ethics Board<br>Henderson Campus<br>90 Wing, Room #1<br>711 Concession Street<br>Hamilton, ON L8V 1C3<br>CANADA
1064	Dr. Sylvie Trottier		Dr. Michel G. Bergeron<br>Dr. Guy Boivin<br>Dr. Louise Cote<br>Dr. Helene Senay	Centre Hospitalier de l'Universite Laval (CHUL)<br>2705 boulevard Laurier<br>Sainte-Foy, QC G1V 4G2<br>CANADA	CHUL du CHUQ<br>2705 Boulevard Laurier<br>Sainte-foy, QC G1V 4G2<br>CANADA
1065	Dr. Christos Tsoukas		Dr. Joseph Cox<br>Dr. Julian M. Falutz<br>Dr. Andreas Giannakis<br>Dr. Norbert Gilmore<br>Dr. Jason Szabo<br>Dr. Howard Turner	Montreal General Hospital, Immune Deficiency Treatment Centre, McGill University Health Centre<br>Room A5-140<br>1650 Cedar Avenue<br>Montreal, QC H3G 1A4<br>CANADA	Biomedical D REB of the McGill University Health Centre<br>McGill University Health Centre<br>Suite C10-148<br>1650 Cedar<br>Montreal, QC H3G 1A4<br>CANADA

* Did not randomize subjects

Center	Principal Investigator	Co-Investigator(s)	Sub-Investigator(s)	Address(es)	Institutional Review Board or Ethics Committee Address(es)
1066	Dr. Sharon Lynn Walmsley		Dr. Wayne Lawrence Gold<br>Dr. Rupert Kaul<br>Dr. Kenneth M. Logue<br>Dr. Jeff Powis<br>Dr. Irving E. Salit	University Health Network / Toronto General Hospital<br>585 University Avenue, 5A-West<br>Toronto, ON M5G 2N2<br>CANADA	University Health Network Research Ethics Board<br>Room 8-18<br>700 University Avenue, 8th Floor South<br>Toronto, ON M5G 1Z5<br>CANADA
1067	Dr. Benoit Trottier		Dr. Michel Boissonnault<br>Dr. Louise Charest<br>Dr. Harold Dion<br>Dr. Stephane Lavoie<br>Dr. Danielle Legault<br>Dr. Daniele Longpre<br>Dr. Pierre-Jean Maziade<br>Dr. Daniel Murphy<br>Dr. Vinh-Kim Nguyen<br>Dr. Robert O'Brien<br>Dr. Rejean Thomas<br>Dr. Sylvie Vezina<br>Dr. Marc-Andre Charron	Clinique Medicale L'Actuel<br>1130<br>1001 De Maisonneuve Est<br>Montreal, QC H2L 4P9<br>CANADA	IRB Services<br>Suite 328<br>14845-6 Yonge Street<br>Aurora, ON L4G 6H8<br>CANADA

090177e1801b1594\Approved\Approved On: 24-Sep-2007 20:04

* Did not randomize subjects

Center	Principal Investigator	Co-Investigator(s)	Sub-Investigator(s)	Address(es)	Institutional Review Board or Ethics Committee Address(es)
1114	Dr. Richard G. Lalonde		Dr. James Allan<br>Dr. Norbert Gilmore<br>Dr. Marina Klein<br>Dr. Roger P. Leblanc<br>Dr. John MacLeod<br>Dr. Martin Potter<br>Dr. Pierre Rene<br>Dr. Jean-Pierre Routy	Montreal Chest Institute<br>3650 St-Urbain #J803<br>Montreal, QC H2X 2P4<br>CANADA	Biomedical D REB of the McGill University Health Centre<br>McGill University Health Centre<br>Suite C10-148<br>1650 Cedar<br>Montreal, QC H3G 1A4<br>CANADA
1115 *	Dr. Barbara Romanowski			University of Alberta - College Plaza<br>1000-8215 112 Street NW<br>Edmonton, AB T6G 2C8<br>CANADA	Health Research Ethics Board, University of Alberta<br>213 Heritage Medical Research Centre<br>Edmonton, AB T6G 2S2<br>CANADA
1116 *	Dr. Ethan Rubinstein<br>Dr. Stuart J. Rosser<br>(Previous PI)		Dr. Ken Kasper<br>Dr. Evelyn W. Lo	St. Boniface General Hospital<br>Section of Infectous Diseases<br>Room N4 -047<br>409 Tachi Avenue<br>Winnipeg, MB R2H 2A6<br>CANADA	Biomedical Research Ethics Board<br>Bannatyne Campus<br>P126 Pathology Building<br>770 Bannatyne Avenue<br>Winnipeg, MB R3E OW3<br>CANADA

* Did not randomize subjects

090177e1801b1594\Approved\Approved On: 24-Sep-2007 20:04

Center	Principal Investigator	Co-Investigator(s)	Sub-Investigator(s)	Address(es)	Institutional Review Board or Ethics Committee Address(es)
1117	Dr. Cecile Tremblay		Dr. Alexandra de Pokomandy<br>Dr. Claude Fortin<br>Dr. Danielle Rouleau<br>Dr. Emil Toma	CHUM, Notre-Dame<br>1560 Sherbrooke Est<br>Montreal, QC H2L 4M1<br>CANADA<br><br>Hotel - Dieu du CHUM / Jeanne Mance Pavillion<br>3rd Floor<br>3840 rue St-Urbain<br>Montreal, QC H2W 1T8<br>CANADA	Comite d ethique de la recherche du CHUM<br>Edifice Cooper<br>3981 boul, St-Laurent<br>Mezzanine 2, bureau M-207<br>Montreal, QC H2W1Y5<br>CANADA
1136	Dr. Roger P. Leblanc			Projet L.O.R.I.<br>Suite 023<br>3545 Cote des Neiges<br>Montreal, QC H3H 1V1<br>CANADA	Ethica Clinical Research Inc.<br>Ethica Clinical Research Inc.<br>Suite 210<br>1255 Transcanada Highway<br>Dorval (Montreal), QC H9P 2V4<br>CANADA

090177e1801b1594\Approved\Approved On: 24-Sep-2007 20:04

090177e1801b1594\Approved\Approved On: 24-Sep-2007 20:04

United States

Coordinating Investigators:

<None Entered>

Center	Principal Investigator	Co-Investigator(s)	Sub-Investigator(s)	Address(es)	Institutional Review Board or Ethics Committee Address(es)
1001	Dr. Bisher Akil		Ann Johiro	Bisher Akil Medical Corp<br>Suite 812<br>9201 Sunset Boulevard<br>Los Angeles, CA 90069<br>UNITED STATES	Schulman Associates Institutional Review Board, Inc.<br>4290 Glendale-Milford Road<br>Cincinnati, OH 45242<br>UNITED STATES
1003	Dr. Roberto Claudio Arduino		Dr. Ben Joseph Barnett<br>Dr. Andrea M.E. Holmes<br>Dr. Owen E. McCormack<br>Dr. Mallika Kamana<br>Dr. Samuel Maghidman	Thomas Street Clinic<br>2015 Thomas Street<br>Houston, TX 77009<br>UNITED STATES<br><br>University of Texas Health Sciences Center<br>MSB 6.120<br>6431 Fannin<br>Houston, TX 77030<br>UNITED STATES	University of Texas Health Science Center at Houston<br>The Committee for the Protection of Human Subjects<br>Suite 750<br>University Center Tower<br>7000 Fannin<br>Houston, TX 77030<br>UNITED STATES

* Did not randomize subjects

Center	Principal Investigator	Co-Investigator(s)	Sub-Investigator(s)	Address(es)	Institutional Review Board or Ethics Committee Address(es)
1004	Dr. George William Beatty		Dr. Jody Lawrence<br>Dr. Royce C. Lin<br>Dr. Ian McNicholl<br>Ms. Lauren E. Poole<br>Clarissa A. Ramstead	San Francisco Gen Hosp<br>Bldg. 80W84<br>995 Potrero Ave.<br>San Francisco, CA 94110-2859<br>UNITED STATES	Committee on Human Research<br>Suite 315<br>3333 California Avenue<br>San Francisco, CA 94118<br>UNITED STATES
1005	Dr. Nicholaos C. Bellos		Dr. Rasha Ghurani<br>Yvonne Michelle Lindahl<br>Durward Watson<br>Dr. Robert Waldrup Henderson III<br>Dr. Mark Joseph Hupert	Southwest Infectious Disease Associates, Nicholaos C. Bellos, M.D., P.A.<br>2909 Lemmon Avenue<br>Dallas, TX 75204<br>UNITED STATES	Schulman Associates Institutional Review Board, Inc.<br>4290 Glendale-Milford Road<br>Cincinnati, OH 45242<br>UNITED STATES
1006	Dr. Sky Robert Blue		Dr. Thomas J. Coffman	HIV Services Clinic<br>777 North Raymond<br>Boise, ID 83704<br>UNITED STATES<br><br>Treasure Valley Lab<br>1070 N. Curtis Road<br>Boise, ID 83706<br>UNITED STATES	Schulman Associates IRB, Inc.<br>4290 Glendale-Milford Road<br>Cincinnati, OH 45242<br>UNITED STATES

090177e1801b1594\Approved\Approved On: 24-Sep-2007 20:04

Center	Principal Investigator	Co-Investigator(s)	Sub-Investigator(s)	Address(es)	Institutional Review Board or Ethics Committee Address(es)
1007	Dr. Paul Joseph Cimoch		Dr. Rosemarie Melchor MD<br>Dr. Charles Michael Walworth<br>Susan Wellborn	Orange County Center for Special Immunology<br>Suite 411<br>11190 Warner Avenue<br>Fountain Valley, CA 92708<br>UNITED STATES	Fountain Valley Regional Hospital Institutional Review Board<br>11190 Warner Ave Ste 306<br>Fountain Valley, CA 92708<br>UNITED STATES
1008	Dr. Calvin Jay Cohen		Dr. Jonathan S. Appelbaum<br>Dr. Amy E. Colson<br>Ms. Colleen P. Corcoran<br>Julia Green<br>Jodi A. Jensen<br>Karen McLaughlin<br>Dr. Harry Schrager<br>Mr. Alexander G. Sheble-Hall<br>Ms. Clarissa B. Foy<br>Susan Glasper<br>Dr. Benjamin P. Linas<br>Dr. St. John D. McGrath<br>Dr. Anne Burnett Morris	Community Research Initiative of New England<br>23 Miner Street<br>Boston, MA 02215<br>UNITED STATES	New England IRB<br>40 Washington Street, Ste 130<br>Wellesley, MA 02481<br>UNITED STATES

090177e1801b1594\Approved\Approved On: 24-Sep-2007 20:04

Center	Principal Investigator	Co-Investigator(s)	Sub-Investigator(s)	Address(es)	Institutional Review Board or Ethics Committee Address(es)
1010	Dr. Stockton Edward Roberts<br>Dr. John David Brand (Previous PI)		Cindy D. Harris	MedicalEdge Healthcare Group, PA<br>Suite 101<br>801 North Zang Boulevard<br>Dallas, TX 75208<br>UNITED STATES	Schulman Associates Institutional Review Board, Incorporated<br>4290 Glendale - Milford Road<br>Cincinnati, OH 45242<br>UNITED STATES
1011	Dr. Gregg Oscar Coodley		Dr. Marcia Kerensky Coodley<br>Dr. Donald A. Valerio	Fanno Creek Clinic<br>2400 Southwest Vermont Street<br>Portland, OR 97219<br>UNITED STATES	Fanno Creek Institutional Review Board<br>2400 SouthWest Vermont Street<br>Portland, OR 97219<br>UNITED STATES
1012	Dr. Charles Frank Farthing<br>Dr. Alen Voskanian (Previous PI)		Dr. Arash Alborzi<br>Dr. Laveeza Bhatti<br>Dr. Homayoon Khanlou<br>Dr. Mehri S. McKellar<br>Dr. Juan Carlos Ricaurte<br>Dr. Michele Lynn Babaie<br>Dr. Catherine Chien<br>Dr. Susan Sanchez	AHF Research Center<br>Suite 200<br>99 North La Cienaga Boulevard<br>Beverly Hills, CA 90211<br>UNITED STATES	Schulman Associates IRB, Inc.<br>4290 Glendale-Milford Road<br>Cincinnati, OH 45242<br>UNITED STATES

090177e1801b1594\Approved\Approved On: 24-Sep-2007 20:04

Center	Principal Investigator	Co-Investigator(s)	Sub-Investigator(s)	Address(es)	Institutional Review Board or Ethics Committee Address(es)
1013	Dr. Lawrence Edward Feldman		Dr. Jeffrey A. East<br>Dr. Karen L. Raben<br>Harry G. Castro	Raben & Feldman & Research Associates Corporation<br>Suite 400<br>7000 SW 62nd Avenue<br>South Miami, FL 33143<br>UNITED STATES	Schulman Associates IRB, Inc.<br>4290 Glendale-Milford Road<br>Cincinnati, OH 45242<br>UNITED STATES
1014	Dr. Stephen Eliot Follansbee		Dr. Walford Jeffrey Fessel	Kaiser Permanente Investigational Drugs Pharmacy (drug shipment)<br>Suite 101<br>4131 Geary Boulevard<br>San Francisco, CA 94118<br>UNITED STATES<br><br>Kaiser Permanente Medical Center<br>Clinical Trials Unit, Suite 219<br>4141 Geary Boulevard<br>San Francisco, CA 94118<br>UNITED STATES	Kaiser Permanente Northern California IRB<br>Kaiser Foundation Research Institute<br>16th Floor<br>1800 Harrison Street<br>Oakland, CA 94612-3431<br>UNITED STATES

090177e1801b1594\Approved\Approved On: 24-Sep-2007 20:04

Center	Principal Investigator	Co-Investigator(s)	Sub-Investigator(s)	Address(es)	Institutional Review Board or Ethics Committee Address(es)
1015	Dr. Joel Emanuel Gallant		Dr. Patricia Andrea Barditch-Crovo<br>Ms. Lorena Coburn<br>Mr. Jason Farley<br>Ms. Jeanne Keruly<br>Mrs. Michelle A. Parish<br>Charles Phillip Raines<br>Dr. James Shepherd	John Hopkins At Greenspring Station<br>10755 Falls Road<br>Lutherville, MD 21093<br>UNITED STATES<br><br>John Hopkins University (admin use)<br>Room 453<br>1830 East Monument Street<br>Baltimore , MD 21287<br>UNITED STATES<br><br>Johns Hopkins Medical Institutions<br>Carnegie 3<br>600 North Wolfe Street<br>Baltimore, MD 21287<br>UNITED STATES	Western Institutional Review Board<br>3535 7th Avenue, Southwest<br>Olympia, WA 98502-5010<br>UNITED STATES

090177e1801b1594\Approved\Approved On: 24-Sep-2007 20:04

Center	Principal Investigator	Co-Investigator(s)	Sub-Investigator(s)	Address(es)	Institutional Review Board or Ethics Committee Address(es)
1017	Dr. Eliot Warren Godofsky		Michele A. Mays<br>Dr. Tanya Schreibman<br>Dr. James Donald DeMaio Jr.	Bach & Godofsky MD, PA<br>Suite 210<br>8451 Shade Avenue<br>Sarasota, FL 34243<br>UNITED STATES<br><br>University Hepatitis Center at Bach & Godofsky<br>Suite 215<br>2415 University Parkway<br>Sarasota, FL 34243<br>UNITED STATES	Schulman Associates Institutional Review Board, Incorporated<br>4290 Glendale - Milford Road<br>Cincinnati, OH 45242<br>UNITED STATES

090177e1801b1594\Approved\Approved On: 24-Sep-2007 20:04

Center	Principal Investigator	Co-Investigator(s)	Sub-Investigator(s)	Address(es)	Institutional Review Board or Ethics Committee Address(es)
1018	Dr. Richard Harold Haubrich		Dr. Constance Ann Benson<br>Tari Gilbert<br>Dr. Miguel Goicoechea<br>Ms. Julie Hoffman<br>Ms. Jill Kunkel<br>Dr. Scott Letendre<br>Dr. Susan Janet Little<br>Ms. Linda Meixner<br>Leticia Muttera<br>Ms. Kathy Nuffer<br>Ms. Paula Potter<br>Ms. Doreen Redmile<br>Ms. Joanne Santangelo<br>Mr. Edward Seefried	UCSD Antiviral Research Center (AVRC)<br>Leticia Muttera, PharmD (drug shipment)<br>Suite 100<br>150 West Washington Street<br>San Diego, CA 92103-2005<br>UNITED STATES<br><br>UCSD Antiviral Research Center (AVRC)<br>Suite 100<br>150 West Washington Street<br>San Diego, CA 92103-2005<br>UNITED STATES	UCSD IRB, Human Subjects Program (0052)<br>La Jolla Professional Building, Suite 2145<br>8950 Villa La Jolla Drive<br>La Jolla, CA 92037<br>UNITED STATES
1019	Dr. William Keith Henry		Dr. Ronald Schut	Hennepin County Medical Center/ Minneapolis Medical Research Foundation<br>Red-7<br>701 Park Avenue<br>Minneapolis, MN 55145<br>UNITED STATES	Hennepin County Medical Center<br>Human Subjects Research Committee<br>900 HFA Building<br>914 South 8th Street<br>Minneapolis, MN 55404<br>UNITED STATES

* Did not randomize subjects

090177e1801b1594\Approved\Approved On: 24-Sep-2007 20:04

Center	Principal Investigator	Co-Investigator(s)	Sub-Investigator(s)	Address(es)	Institutional Review Board or Ethics Committee Address(es)
1020	Dr. David Holden Henry III		Dr. Patricia Ann Ford<br>Clara Granda-Cameron<br>Dr. Lee Hartner<br>Dr. Michael Joel Haut<br>Nancy Leahy<br>Dr. Bernard Arthur Mason<br>Dr. David Michael Mintzer<br>Dr. Arthur Petrie Staddon<br>Nancy Zieber	Pennsylvania Oncology Hematology Associates<br>2nd Floor<br>230 W. Washington Square<br>Philadelphia, PA 19106<br>UNITED STATES	Pennsylvania Hospital<br>Research Review Committee<br>800 Spruce Street<br>Philadelphia, PA 19107<br>UNITED STATES
1021	Dr. Jose Norberto Hernandez		Dr. Juan Antonio Enriquez	SBMA<br>Suite 310<br>333 41st Street<br>Miami, FL 33140<br>UNITED STATES	Schulman Associates IRB, Inc.<br>4290 Glendale-Milford Road<br>Cincinnati, OH 45242<br>UNITED STATES

090177e1801b1594\Approved\Approved On: 24-Sep-2007 20:04

Center	Principal Investigator	Co-Investigator(s)	Sub-Investigator(s)	Address(es)	Institutional Review Board or Ethics Committee Address(es)
1022	Dr. Frederick A. Cruickshank<br>Dr. Joseph Gregory Jemsek (Previous PI)		Aimee O. Buck<br>Octavio Cieza<br>Christie Furr Roeske<br>Joel Wesley Thompson	Jemsek Clinic<br>Suite 200<br>14330 Oakhill Park Lane<br>Huntersville, NC 28078<br>UNITED STATES<br><br>Rosedale Infectious Diseases<br>Suite 202<br>14330 Oakhill Park Lane<br>Huntersville, NC 28078<br>UNITED STATES	Schulman Associates Institutional Review Board, Inc.<br>4290 Glendale-Milford Road<br>Cincinnati, OH 45242<br>UNITED STATES
1023	Dr. Jacob Paul Lalezari		Felicia L. Sterman MD	Quest Clinical Research<br>Ste 202<br>2300 Sutter St<br>San Francisco, CA 94115-3029<br>UNITED STATES	Schulman Associates Institutional Review Board, Inc.<br>4290 Glendale-Milford Road<br>Cincinnati, OH 45242<br>UNITED STATES

090177e1801b1594\Approved\Approved On: 24-Sep-2007 20:04

090177e1801b1594\Approved\Approved On: 24-Sep-2007 20:04

Center	Principal Investigator	Co-Investigator(s)	Sub-Investigator(s)	Address(es)	Institutional Review Board or Ethics Committee Address(es)
1024	Dr. Harry William Lampiris		Dr. Oliver Bacon<br>Dr. Peter Christian Jensen<br>Dr. Joseph Kai Wong	Research Pharmacy<br>San Francisco Veterans Affairs Medical Center<br>MS 119<br>4150 Clement Street<br>San Francisco, CA 94121<br>UNITED STATES<br><br>San Francisco Veterans Affairs Medical Center<br>Infectious Disease Section 111W<br>4150 Clement Street<br>San Francisco, CA 94121<br>UNITED STATES	University of California, San Francisco<br>Committee on Human Research<br>Suite 315<br>3333 California Street<br>San Francisco, CA 94118<br>UNITED STATES

* Did not randomize subjects

Center	Principal Investigator	Co-Investigator(s)	Sub-Investigator(s)	Address(es)	Institutional Review Board or Ethics Committee Address(es)
1026	Dr. Donna Mildvan		Gwendolyn D. Costantini FNP<br>Donald S. Garmon<br>Sondra M. Middleton<br>Dr. Nadim Salomon<br>Dr. Ronald D. D'Amico<br>Dr. Jeffrey Martin Jacobson	Beth Israel Medical Center<br>Investigational Drug Service<br>Department of Pharmacy<br>Silver Building, Lobby Level<br>330 East 17th Street<br>New York, NY 10003<br>UNITED STATES<br><br>Beth Israel Medical Center<br>Peter Krueger Clinic<br>317 East 17th Street<br>New York, NY 10003<br>UNITED STATES<br><br>Beth Israel Medical Center<br>Division of Infectious Diseases<br>AIDS Clinical Trials Unit<br>350 East 17th Street<br>New York, NY 10003<br>UNITED STATES	Beth Israel Medical Center<br>Human Subjects Division<br>Committee on Scientific Activities<br>First Avenue @ 16th Street<br>New York, NY 10003<br>UNITED STATES
1027	Dr. Ronald Dean Wilcox		Dr. Rebecca Adair Clark	Ronald Dean Wilcox, MD<br>HOP Clinic<br>136 South Roman Street<br>New Orleans, LA 70112<br>UNITED STATES	Louisiana State University Medical Center<br>Institutional Review Board<br>433 Bolivar Street<br>New Orleans, LA 70112<br>UNITED STATES

* Did not randomize subjects

090177e1801b1594\Approved\Approved On: 24-Sep-2007 20:04

090177e1801b1594\Approved\Approved On: 24-Sep-2007 20:04

Center	Principal Investigator	Co-Investigator(s)	Sub-Investigator(s)	Address(es)	Institutional Review Board or Ethics Committee Address(es)
1029	Dr. Robert Anderson Myers Jr.		Brian Arey<br>Dr. Anita Jeanne Culp<br>Dr. John Mark Post<br>Adrianne Kazmier	Phoenix Body Positive<br>Suite 200<br>1144 East McDowell Road<br>Phoenix, AZ 85006<br>UNITED STATES	Schulman Associates Institutional Review Board, Incorporated<br>4290 Glendale - Milford Road<br>Cincinnati, OH 45242<br>UNITED STATES
1030	Dr. Daniel Dysart Pearce		Dr. Felix Francisco Carpio-Cedraro<br>Debra Johnson<br>Deanna Perez	Altamed Health Services/ Clinical Trials Unit<br>5427 East Whittier Boulevard<br>Los Angeles, CA 90022<br>UNITED STATES	Schulman Associates IRB, Inc.<br>4290 Glendale-Milford Road<br>Cincinnati, OH 45242<br>UNITED STATES
1031	Dr. Gerald Pierone Jr.		Dorothy Bulgin-Coleman<br>Chandra Devi Kantor ARNP	Treasure Coast Infectious Disease Consultants<br>3715 7th Terrace<br>Vero Beach, FL 32960<br>UNITED STATES	Schulman Associates Institutional Review Board, Incorporated<br>4290 Glendale - Milford Road<br>Cincinnati, OH 45242<br>UNITED STATES
1032	Dr. Jayashree Ravishankar		Dr. Jack Alan DeHovitz<br>Susan Holman<br>Dr. Jesi Ramone<br>Lisa Shipper<br>Ronald W. Harris<br>Mr. Gabriel Larson<br>Dr. Cyril Cinco Llamoso	University Hospital of Brooklyn<br>State University of New York Downstate Medical Center<br>450 Clarkson Avenue<br>Brooklyn, NY 11203<br>UNITED STATES	State University of New York Downstate Medical Center<br>Institutional Review Board<br>Box 129<br>450 Clarkson Avenue<br>Brooklyn, NY 11203<br>UNITED STATES

* Did not randomize subjects

Center	Principal Investigator	Co-Investigator(s)	Sub-Investigator(s)	Address(es)	Institutional Review Board or Ethics Committee Address(es)
1033	Dr. Robert Ray Redfield Jr.		Dr. Anthony Amoroso<br>Dr. Charles Edward Davis Jr.<br>Dr. Bruce L. Gilliam<br>Dr. Ronald B. Reisler<br>Dr. Daniel A. Wolde-Rufael	University of Maryland Institute of Human Virology<br>725 West Lombard Street<br>Baltimore, MD 21201<br>UNITED STATES	University of Maryland, Office of Research Subjects<br>Health Sciences Facility (HSF-1)<br>Room 146<br>685 West Baltimore Street<br>Baltimore, MD 21201-1559<br>UNITED STATES
1034	Dr. Richard Craig Reichman		Dr. Susan Ellen Cohn<br>Dr. Lisa Maria Demeter<br>Ms Carol Greisberger<br>Dr. Christine Koval<br>Dr. Amneris Esther Luque<br>Dr. Peter Raymond Mariuz<br>Jane A. Reid	University of Rochester Medical Center<br>601 Elmwood Avenue<br>Rochester, NY 14642<br>UNITED STATES	WIRB<br>P O Box 12029<br>3535 7th Avenue Southwest<br>Olympia, WA 98502<br>UNITED STATES
1035	Dr. William Jay Robbins			Infectious Diseases of Central Florida PA<br>Suite 300<br>1720 South Orange Avenue<br>Orlando, FL 32806<br>UNITED STATES	Schulman Associates Institutional Review Board, Incorporated<br>4290 Glendale - Milford Road<br>Cincinnati, OH 45242<br>UNITED STATES

* Did not randomize subjects

090177e1801b1594\Approved\Approved On: 24-Sep-2007 20:04

Center	Principal Investigator	Co-Investigator(s)	Sub-Investigator(s)	Address(es)	Institutional Review Board or Ethics Committee Address(es)
1036	Dr. Michael Saag		Dr. Laura Hinkle Bachmann<br>Dr. John Wyatt Gnann Jr.<br>Dr. Paul Andres Goepfert<br>Dr. Sonya L. Heath<br>Dr. Victoria Anne Johnson<br>Dr. John Michael Kilby<br>Dr. Michael James Mugavero<br>Dr. Mukesh Patel<br>Jennifer Peterson<br>Dr. Amy Player<br>James L. Raper<br>Laura P. Secord<br>Dr. Ming Walker<br>Dr. James M. Sizemore	University of Alabama at Birmingham 1917 Clinic<br>Community Care Building / 2nd Floor<br>908 20th Street South<br>Birmingham, AL 35294<br>UNITED STATES	Western Institutional Review Board<br>3535 Seventh Avenue, SW<br>Olympia, WA 98502<br>UNITED STATES

090177e1801b1594\Approved\Approved On: 24-Sep-2007 20:04

090177e1801b1594\Approved\Approved On: 24-Sep-2007 20:04

Center	Principal Investigator	Co-Investigator(s)	Sub-Investigator(s)	Address(es)	Institutional Review Board or Ethics Committee Address(es)
1037	Dr. Richard Alan Elion<br>Dr. Kunthavi Sathasivam (Previous PI)		Dr. Joseph Baker<br>Tina Celenza<br>Dr. Roxanne D. Cox-Iyamu<br>Luke Johnsen<br>Barbara R. Lewis<br>Dr. Ambalavanapillai Mathivannan<br>Dr. Bryan Baugh<br>Dr. Philippe Albert Chiliade<br>Danbi Mallin	Whitman Walker Clinic<br>1701 14th Street NW<br>Washington, DC 20009<br>UNITED STATES	Whitman-Walker Clinic Institutional Review Board<br>1701 14th St. NW<br>Washington, DC 20009<br>UNITED STATES
1038	Dr. Lawrence E. Schwartz		Dr. Marina Arbuck<br>Ann E. Hyder<br>Dr. Elizabeth Anne Lien<br>Dr. Peter K. Marsh<br>Dr. David Winters McEniry<br>Dr. Romana V. Popa<br>Dr. Philip Cheney Craven	Northwest Medical Specialties, PLLC<br>Infections Limited PS<br>Suite B<br>220 15th Ave SE<br>Puyallup, WA 98372<br>UNITED STATES<br><br>Northwest Medical Specialties, PLLC<br>Suite 405<br>1624 South I Street<br>Tacoma, WA 98405<br>UNITED STATES	Schulman Associates IRB, Inc.<br>4290 Glendale-Milford Road<br>Cincinnati, OH 45242<br>UNITED STATES

* Did not randomize subjects

Center	Principal Investigator	Co-Investigator(s)	Sub-Investigator(s)	Address(es)	Institutional Review Board or Ethics Committee Address(es)
1039	Dr. Louis Marshall Sloan		Dr. Marc Tribble	North Texas Infectious Diseases Consultants PA<br>Suite 710<br>3409 Worth Street<br>Dallas, TX 75246<br>UNITED STATES	Schulman Associates Institutional Review Board, Inc.<br>4290 Glendale-Milford Road<br>Cincinnati, OH 45242<br>UNITED STATES
1041	Dr. Clifford A. Kinder<br>Dr. Corklin Ray Steinhart (Previous PI)		Dr. Michelle Powell<br>Dr. David E. Schmitt<br>Dr. Allan John Stein<br>Amy Sue Liebmann<br>Dr. Isabela B. Sierra	Steinhart Medical Associates<br>Suite 806<br>Mercy Professional Bldg.<br>3661 S. Miami Ave..<br>Miami, FL 33133<br>UNITED STATES<br><br>Steinhart Medical Associates<br>Suite 810<br>3661 South Miami Avenue<br>Miami, FL 33133<br>UNITED STATES	Schulman Associates Institutional Review Board, Inc.<br>4290 Glendale-Milford Road<br>Cincinnati, OH 45242<br>UNITED STATES

* Did not randomize subjects

090177e1801b1594\Approved\Approved On: 24-Sep-2007 20:04

Center	Principal Investigator	Co-Investigator(s)	Sub-Investigator(s)	Address(es)	Institutional Review Board or Ethics Committee Address(es)
1042	Dr. Melanie Ann Thompson		Dacenta A. Grice	AIDS Research Consortium of Atlanta, Incorporated<br>Suite 130<br>131 Ponce de Leon Avenue<br>Atlanta, GA 30308<br>UNITED STATES	AIDS Research Consortium of Atlanta Incorporated-Institutional Review Board<br>Suite 130<br>131 Ponce de Leon Avenue<br>Atlanta, GA 30308<br>UNITED STATES
1043	Dr. William James Towner Jr.		Dr. Joseph C. Chang<br>Dr. Hai Linh Kerrigan<br>Dr. Marc J. LaRiviere<br>Dr. John P. Martin<br>Dr. Jim H. Nomura<br>Dr. Tomiko Stein<br>Dr. Kim-Huong Thi Tran<br>Dr. Townson Tsai<br>Karole Velzy<br>Dr. Diane Yamamoto Skowron<br>Dr. Leslie Wang	Kaiser Permanente Medical Center/ Southern CA Permanente Med. Group<br>Infectious Disease/ 2nd Floor<br>1505 North Edgemont Street<br>Los Angeles, CA 90027<br>UNITED STATES	Kaiser Permanente Southern California IRB Research and Evaluation<br>2nd Floor<br>393 East Walnut Street<br>Pasadena, CA 91101<br>UNITED STATES
1045	Silver Sisneros		Dr. Kathleen A. Clanon<br>Dr. Howard Ellis Edelstein<br>Leslie J. Reynolds<br>Ms. Cynthia Rowden<br>Dr. Beth Schweitzer	Alameda County Medical Center, Adult Immunology Clinic<br>1411 East 31st Street<br>Oakland, CA 94602<br>UNITED STATES	ALAMEDA COUNTY MEDICAL CENTER<br>1411 EAST 31ST STREET<br>OAKLAND , CA 94602<br>UNITED STATES

090177e1801b1594\Approved\Approved On: 24-Sep-2007 20:04

Center	Principal Investigator	Co-Investigator(s)	Sub-Investigator(s)	Address(es)	Institutional Review Board or Ethics Committee Address(es)
1046	Dr. David Allen Wheeler		Dr. Mary Beth E. Alder<br>Dr. Sujata H. Ambardar<br>Dr. Allan J. Morrison Jr.<br>Dr. Donald Martin Poretz<br>Dr. Ann I. Rixinger	Infectious Diseases Physicians, Incorporated, Office of Clinical Research<br>Suite 250<br>3289 Woodburn Road<br>Annandale, VA 22003<br>UNITED STATES	Schulman Associates Institutional Review Board, Incorporated<br>4290 Glendale - Milford Road<br>Cincinnati, OH 45242<br>UNITED STATES
1047	Dr. Sally Williams		Dr. David Andrew Bong	The Vancouver Clinic<br>700 Northeast 87th Avenue<br>Vancouver, WA 98664<br>UNITED STATES	Schulman Associates Institutional Review Board, Incorporated<br>4290 Glendale - Milford Road<br>Cincinnati, OH 45242<br>UNITED STATES
1048	Dr. Michael Bruce Wohlfeiler		Dr. Deborah L. Holmes<br>Dr. Joseph R. Piperato	Wohlfeiler, Piperato & Associates, LLC<br>Suite 202<br>16401 N.W. 2nd Ave<br>North Miami Beach, FL 33169<br>UNITED STATES<br><br>Wohlfeiler, Piperato, & Associates, LLC<br>1613 Alton Road<br>Miami Beach, FL 33139<br>UNITED STATES	Schulman Associates IRB, Inc.<br>4290 Glendale-Milford Road<br>Cincinnati, OH 45242<br>UNITED STATES

* Did not randomize subjects

090177e1801b1594\Approved\Approved On: 24-Sep-2007 20:04

Center	Principal Investigator	Co-Investigator(s)	Sub-Investigator(s)	Address(es)	Institutional Review Board or Ethics Committee Address(es)
1050	Dr. Bienvenido Gamulo Yangco			Infectious Disease Research Institute, Inc.<br>Suite 203<br>4620 North Habana Avenue<br>Tampa, FL 33614<br>UNITED STATES	Schulman Associates IRB, Inc.<br>4290 Glendale-Milford Road<br>Cincinnati, OH 45242<br>UNITED STATES
1051	Dr. Barry Stephen Zingman		Julie Naczi Sarlo	Montefiore Medical Center<br>AIDS Clinic<br>111 East 210th Street<br>Bronx, NY 10467<br>UNITED STATES	Biomedical Research Alliance of New York, LLC<br>Institutional Review Board<br>Suite 100<br>225 Community Drive<br>Great Neck, NY 11021<br>UNITED STATES

090177e1801b1594\Approved\Approved On: 24-Sep-2007 20:04

* Did not randomize subjects

090177e1801b1594\Approved\Approved On: 24-Sep-2007 20:04

Center	Principal Investigator	Co-Investigator(s)	Sub-Investigator(s)	Address(es)	Institutional Review Board or Ethics Committee Address(es)
1052	Dr. Steven Williams		Dr. Gregory James Mertz<br>Dr. Corey Alan Tancik<br>Dr. Karla Thornton<br>Dr. Elaine Thomas	University of New Mexico Health Sciences Center<br>University Hospital, Investigational Drug Services<br>2211 Lomas Boulevard NE<br>Albuquerque, NM 87106<br>UNITED STATES<br><br>University of New Mexico Health Sciences Center, Truman Street Clinic<br>625 Truman Street NE<br>Albuquerque, NM 87110<br>UNITED STATES	Human Research Review Committee<br>University of New Mexico Health Sciences Center School of Medicine<br>MSC08 4560<br>1 University of New Mexico<br>Albuquerque, NM 87131-0001<br>UNITED STATES

* Did not randomize subjects

Center	Principal Investigator	Co-Investigator(s)	Sub-Investigator(s)	Address(es)	Institutional Review Board or Ethics Committee Address(es)
1053	Dr. Roy Gulick		Dr. Marshall Jay Glesby<br>Ms. Valery Hughes<br>Dr. Kristen Marks<br>Dr. Mary Amelia Vogler<br>Dr. Timothy J. Wilkin	Cornell Clinical Trials Unit, Chelsea Center<br>Ground Floor<br>119 West 24th Street<br>New York, NY 10011<br>UNITED STATES<br><br>Weill Medical College, Cornell Clinical Trial Unit<br>Box 566<br>525 East 68th Street<br>New York, NY 10021<br>UNITED STATES	New York Presbyterian Hospital, Weill Medical College of Cornell University<br>Committee on Human Rights in Research<br>Room A-130<br>525 East 68th Street<br>New York, NY 10021<br>UNITED STATES
1054	Dr. William David Hardy		Dr. Paula Louise Gaut<br>Dr. Aruna Rekha Murthy<br>Dr. Vivian Shirvani	Cedars-Sinai Medical Center/ Infectious Disease<br>Becker 220<br>8700 Beverly Boulevard<br>Los Angeles, CA 90048<br>UNITED STATES	Cedars-Sinai Medical Center Institutional Review Board<br>Suite 742<br>8383 Wilshire Boulevard<br>Beverly Hills, CA 90211<br>UNITED STATES
1068	Dr. Robert Key Bolan		Dr. Michael J. Hall<br>Cynthia Harrison<br>Dr. Matthew Bosse<br>Dr. Gaetano Vaccaro	Jeffrey Goodman Special Care Clinic<br>1625 Schrader Boulevard<br>Los Angeles, CA 90028<br>UNITED STATES	Schulman Associates IRB, Inc.<br>4290 Glendale-Milford Road<br>Cincinnati, OH 45242<br>UNITED STATES

090177e1801b1594\Approved\Approved On: 24-Sep-2007 20:04

090177e1801b1594\Approved\Approved On: 24-Sep-2007 20:04

Center	Principal Investigator	Co-Investigator(s)	Sub-Investigator(s)	Address(es)	Institutional Review Board or Ethics Committee Address(es)
1069	Dr. Laura Kogelman		Dr. James A. Hellinger<br>Dr. Christine Ann Wanke	Tufts University New England Medical Center, Division of Infectious Diseases<br>Box 238<br>750 Washington Street<br>Boston, MA 02111<br>UNITED STATES	Tufts- New England Medical Center Clinical Labortatories<br>#817<br>750 Washington Street<br>Boston, MA 02111<br>UNITED STATES
1071	Dr. Edwin DeJesus		Dr. Anthony Lee<br>Dr. Roberto Ortiz	Orlando Immunology Center<br>1701 North Mills Ave<br>Orlando, FL 32803<br>UNITED STATES	Schulman Associates Institutional Review Board, Inc.<br>4290 Glendale-Milford Road<br>Cincinnati, OH 45242<br>UNITED STATES
1072	Dr. Jerome A. Ernst		Dr. Yuriy S. Akulov<br>Dr. Douglas G. Mendez	ACRIA<br>17th Floor<br>230 West 38th Street<br>New York, NY 10018<br>UNITED STATES	Schulman Associates Institutional Review Board, Incorporated<br>4290 Glendale - Milford Road<br>Cincinnati, OH 45242<br>UNITED STATES
1073	Dr. Roberto B. Corales<br>Dr. Steven Mark Fine<br>(Previous PI)		Dr. Michael L. Christie<br>Deborah J. Dougherty<br>Nancy A. Woods	Aids Community Health Center<br>4th Floor<br>87 N. Clinton Avenue<br>Rochester, NY 14604<br>UNITED STATES	Western Institutional Review Board<br>3535 Seventh Avenue, SW<br>Olympia, WA 98502<br>UNITED STATES

* Did not randomize subjects

Center	Principal Investigator	Co-Investigator(s)	Sub-Investigator(s)	Address(es)	Institutional Review Board or Ethics Committee Address(es)
1074	Dr. Stephen P. Hauptman			Hauptman Family Health Center<br>Suite 303<br>2000 Hamilton Street<br>Philadelphia, PA 19130<br>UNITED STATES	Schulman Associates Institutional Review Board, Incorporated<br>4290 Glendale - Milford Road<br>Cincinnati, OH 45242<br>UNITED STATES
1075	Dr. Shawn K. Hassler		Dr. Ivor A. Emanuel<br>Mr. Martin C. Kramer	Benchmark Research<br>Suite 1415<br>490 Post Street<br>San Francisco, CA 94102<br>UNITED STATES<br><br>Benchmark Research<br>Business Offices<br>Suite 1442<br>490 Post Street<br>San Francisco, CA 94102<br>UNITED STATES<br><br>Office of Shawn K. Hassler, MD<br>Suite 600<br>870 Market Street<br>San Francisco, CA 94102<br>UNITED STATES	Schulman Associates Institutional Review Board, Inc.<br>4290 Glendale-Milford Road<br>Cincinnati, OH 45242<br>UNITED STATES

090177e1801b1594\Approved\Approved On: 24-Sep-2007 20:04

090177e1801b1594\Approved\Approved On: 24-Sep-2007 20:04

Center	Principal Investigator	Co-Investigator(s)	Sub-Investigator(s)	Address(es)	Institutional Review Board or Ethics Committee Address(es)
1076 *	Dr. Barbara Marie Gripshover		Dr. Robert Asaad<br>Dr. Michael M. Lederman<br>Dr. Michelle V. Lisgaris<br>Dr. Grace A. McComsey<br>Dr. Robert Andrew Salata	University Hospitals of Cleveland<br>Clinical Trials Unit<br>2061 Cornell Road<br>Cleveland, OH 44106<br>UNITED STATES	University Hospitals of Cleveland<br>11100 Euclid Avenue<br>Cleveland, OH 44106<br>UNITED STATES
1077	Dr. Jeffrey Lloyd Lennox		Dr. Carlos Chiriboga Del Rio<br>Dr. Ighovwerha Ofotokun<br>Dr. Sophie A. Lukashok	Emory University<br>Infectious Disease Program,<br>Ponce de Leon Center<br>341 Ponce DeLeon Avenue NE<br>Atlanta, GA 30308<br>UNITED STATES	Institutional Review Board<br>James Keller, MD Chairman<br>1256 Briarcliff Road<br>4th Floor, South Wing<br>Atlanta, GA 30306<br>UNITED STATES
1078 *	Dr. Harold Luther Martin Jr.		Dr. Leslie A. Baken	Park Nicollet Health Services, Park Nicollet Clinic<br>3850 Park Nicollet Boulevard<br>Minneapolis, MN 55416<br>UNITED STATES<br><br>Park Nicollet Health Services, Park Nicollet Institute<br>3800 Park Nicollet Boulevard<br>Minneapolis, MN 55416<br>UNITED STATES	Park Nicollet IRB<br>3800 Park Nicollet Boulevard<br>Minneapolis, MN 55416<br>UNITED STATES

* Did not randomize subjects

090177e1801b1594\Approved\Approved On: 24-Sep-2007 20:04

Center	Principal Investigator	Co-Investigator(s)	Sub-Investigator(s)	Address(es)	Institutional Review Board or Ethics Committee Address(es)
1079	Dr. Joseph P. McGowan		Dr. Marcia Ellen Epstein<br>Dr. Bruce Hirsch<br>Dr. David Hirschwerk<br>Dr. Angela Kim<br>Dr. Yelena Markovskaya	North Shore University Hospital<br>4th Floor, Lippert Building<br>300 Community Drive<br>Manhasset, NY 11030<br>UNITED STATES	Biomedical Research Alliance of New York, LLC<br>Suite 100<br>225 Community Drive<br>Great Neck, NY 11021<br>UNITED STATES
1080	Dr. Ronald Takeshi Mitsuyasu		Dr. Margrit Ellen Carlson<br>Dr. Emery Chang<br>Dr. Judith S. Currier<br>Dr. Matthew Leibowitz<br>Dr. Ardis Moe<br>Dr. Obiamiwe C. Umeh MD	UCLA CARE Clinic<br>Suite 100<br>1399 Roxbury Drive<br>Los Angeles, CA 90035<br>UNITED STATES	Medical Human Subjects Protection Committee<br>2107 Peter V. Ueberroth Building<br>10945 Le Conte Avenue<br>Los Angeles, CA 90095<br>UNITED STATES
1081	Dr. Ralph Liporace<br>Dr. George Louis Drusano (Previous PI)		Minda Hubbard	Albany Medical College<br>47 New Scotland Avenue<br>Albany, NY 12208<br>UNITED STATES<br><br>Albany Medical College<br>MC-158<br>66 Hackett Boulevard<br>Albany, NY 12208<br>UNITED STATES	Western Institutional Review Board<br>3535 Seventh Avenue, SW<br>Olympia, WA 98502<br>UNITED STATES

Center	Principal Investigator	Co-Investigator(s)	Sub-Investigator(s)	Address(es)	Institutional Review Board or Ethics Committee Address(es)
1082	Dr. Bruce Stephen Rashbaum		Mr. Thomas A. Kantor<br>Dr. Sylvia R. Medley	Capital Medical Associates, P.C.<br>Suite 800<br>1640 Rhode Island Avenue, NW<br>Washington, DC 20036<br>UNITED STATES	Schulman Associates Institutional Review Board, Inc.<br>4290 Glendale-Milford Road<br>Cincinnati, OH 45242<br>UNITED STATES
1084 *	Dr. Sorana Segal-Maurer		Dr. Tom Chaing<br>Dr. Cruz Fana<br>Dr. Dragana Orloviz<br>Dr. David Scott Rubin<br>Dr. Wehbeh Wehbeh	New York Hospital Queens<br>Special Care Center<br>56-45 Main Street<br>Flushing, NY 11355<br>UNITED STATES	New York Hospital Queens IRB<br>Lang Research Center<br>56-45 Main Street<br>Flushing, NY 11355<br>UNITED STATES
1085	Dr. Paul Richard Skolnik		Ms. Betsy Adams<br>Dr. Maura A. Fagan<br>Dr. Jon Fuller<br>Ms. Charlene Gaca<br>Ms. Ruth Haivanis<br>Myrlande Jules-Villefranche	Boston Medical Center<br>Center for HIV/AIDS Care and Research<br>Dowling Ground<br>850 Harrison Avenue<br>Boston, MA 02118-2393<br>UNITED STATES	Western IRB<br>3535 Seventh Avenue Southwest<br>Olympia, WA 98502<br>UNITED STATES
1086	Dr. James Michael Sosman		Sarah B. Affeldt<br>Dr. Franklin Michael Graziano<br>Dr. Andrew William Urban	University of Wisconsin Hospital and Clinics<br>600 Highland Avenue<br>Madison, WI 53792<br>UNITED STATES	University of Wisconsin - Madison Health Sciences Institutional Review Boards<br>Room B3088 VA Medical Center<br>2500 Overlook Terrace<br>Madison, WI 53705<br>UNITED STATES

* Did not randomize subjects

090177e1801b1594\Approved\Approved On: 24-Sep-2007 20:04

Center	Principal Investigator	Co-Investigator(s)	Sub-Investigator(s)	Address(es)	Institutional Review Board or Ethics Committee Address(es)
1087	Dr. Donna Elaine Sweet		Janis Cerullo<br>Jeannette M. Lanier<br>Dr. Ha Ta<br>Kathryn D. Thiessen	Kansas University Internal Medicine - Midtown<br>1125 North Topeka<br>Wichita, KS 67214<br>UNITED STATES<br><br>Kansas University Medical School - Wichita, Medical Practice Association<br>1010 N Kansas Street<br>Wichita, KS 67214<br>UNITED STATES	KU Medical School-Wichita<br>1010 N Kansas<br>Wichita, KS 67214<br>UNITED STATES
1088	Dr. Susan Swindells		Dr. Ziba Jalali<br>Dr. Miguel Gerardo Madariaga<br>Dr. Joseph Hoagbin<br>Dr. Edna Klingler	University of Nebraska Med Ctr<br>985400 Nebraska Medical Ctr<br>Omaha, NE 68198-5400<br>UNITED STATES	University of Nebraska Medical Center<br>Institutional Review Board<br>987830 Nebraska Medical Center<br>Omaha, NE 68198-7830<br>UNITED STATES

* Did not randomize subjects

090177e1801b1594\Approved\Approved On: 24-Sep-2007 20:04

Center	Principal Investigator	Co-Investigator(s)	Sub-Investigator(s)	Address(es)	Institutional Review Board or Ethics Committee Address(es)
1090	Dr. Pablo Tebas-Medrano		Dr. Ian Frank<br>Mr Joseph H. Quinn<br>Mr. Wayne Wagner	Hospital of the University of Pennsylvania<br>3400 Spruce Street<br>Philadelphia, PA 19104<br>UNITED STATES<br><br>Infectious Disease Clinical Trials Unit<br>834 Penn Tower<br>Philadelphia, PA 19104<br>UNITED STATES	University of Pennsylvania<br>Committee of Studies Involving Human Beings/Office of Regulatory Affairs<br>Mezzanine Level<br>133 South 36th Street<br>Philadelphia, PA 19104<br>UNITED STATES
1093 *	Dr. Stephen Lawrence Becker		Mark L. Illeman<br>Ellen K. Opie<br>Dr. Lorna Marie Thornton	Pacific Horizon Medical Group<br>Suite 512<br>2351 Clay Street<br>San Francisco, CA 94115<br>UNITED STATES	Schulman Associates IRB, Inc.<br>4290 Glendale-Milford Road<br>Cincinnati, OH 45242<br>UNITED STATES

* Did not randomize subjects

090177e1801b1594\Approved\Approved On: 24-Sep-2007 20:04

Center	Principal Investigator	Co-Investigator(s)	Sub-Investigator(s)	Address(es)	Institutional Review Board or Ethics Committee Address(es)
1094	Dr. Rafael E. Campo		Dr. Dushyantha T. Jayaweera<br>Mr. Christopher G. Kaszubski<br>Rose Lalanne<br>Karen Marshall<br>Sandra Mercado	Jackson Memorial Hospital<br>West Wing Basement Pharmacy B26<br>1611 NW 12th Avenue<br>Miami, FL 33136<br>UNITED STATES	Western IRB<br>3535 Seventh Avenue Southwest<br>Olympia, WA 98502<br>UNITED STATES
			Dr. Allan Eduardo Rodriguez<br>Mr. Thomas J. Tanner	University of Miami School of Medicine<br>Infectious Diseases Clinical Research Unit<br>8th Floor - West<br>1500 NW 12th Avenue<br>Miami, FL 33136<br>UNITED STATES	
1095	Dr. Leslie E. Diaz		Robert J. Schaffer<br>Dr. Laurie A. Welton<br>Patricia Ann Whalen	Infectious Disease of the Palm Beaches, Inc.<br>Suite 120<br>840 US Highway #1<br>North Palm Beach , FL 33408<br>UNITED STATES	Schulman Associates Institutional Review Board, Incorporated<br>4290 Glendale - Milford Road<br>Cincinnati, OH 45242<br>UNITED STATES

090177e1801b1594\Approved\Approved On: 24-Sep-2007 20:04

* Did not randomize subjects

Center	Principal Investigator	Co-Investigator(s)	Sub-Investigator(s)	Address(es)	Institutional Review Board or Ethics Committee Address(es)
1097	Dr. Gary Green			Kaiser Permanente Medical Center<br>HIV Services (drug shipment)<br>MOB East Suite 320<br>401 Bicentennial Way<br>Santa Rosa, CA 95403<br>UNITED STATES<br><br>Santa Rosa Kaiser Medical Center<br>MOB East Suite 320<br>401 Bicentennial Way<br>Santa Rosa, CA 95403<br>UNITED STATES	Kaiser Permanente Northern California IRB<br>Kaiser Foundation Research Institute<br>16th Floor<br>1800 Harrison Street<br>Oakland, CA 94612-3431<br>UNITED STATES
1098 *	Dr. David Burr Clifford<br>Dr. Richard Kevin Groger (Previous PI)		Dr. Erin K. Quirk	Washington University School of Medicine<br>ACTU<br>4570 Children's Place<br>St. Louis, MO 63110<br>UNITED STATES	Washington University Human Studies Committee<br>Suite 233<br>22 North Euclid Avenue<br>St. Louis, MO 63108<br>UNITED STATES

* Did not randomize subjects

090177e1801b1594\Approved\Approved On: 24-Sep-2007 20:04

Center	Principal Investigator	Co-Investigator(s)	Sub-Investigator(s)	Address(es)	Institutional Review Board or Ethics Committee Address(es)
1099	Dr. Peter Gordon		Mr. Steven Chang<br>Dr. Scott Mel Hammer<br>Dr. Christine Hogan<br>Ms. Chrisa Hunnewell<br>Mr. Steven Palmer<br>Dorothy Sawo<br>Michael Yin<br>Ms. Janis Zadel<br>Dr. Christina Koizumi<br>Dr. Angela Talley	CUMC Research Pharmacy<br>IP 749<br>161 Fort Washington Avenue<br>New York, NY 10032<br>UNITED STATES<br><br>New York - Presbyterian Hospital<br>Columbia University Medical Center<br>HP6<br>180 Fort Washtington<br>New York, NY 10032<br>UNITED STATES	Columbia University Medical Center<br>Institutional Review Board<br>4th Floor<br>722 West 168th Street<br>New York, NY 10032<br>UNITED STATES
1100	Dr. Trevor N. Hawkins		Dr. Mary Ellen Lawrence<br>Dr. Michael Dane Palestine	Southwest Care Center<br>Suite E<br>649 Harkle Road<br>Santa Fe, NM 87505<br>UNITED STATES	Schulman Associates Institutional Review Board, Incorporated<br>4290 Glendale - Milford Road<br>Cincinnati, OH 45242<br>UNITED STATES

090177e1801b1594\Approved\Approved On: 24-Sep-2007 20:04

Center	Principal Investigator	Co-Investigator(s)	Sub-Investigator(s)	Address(es)	Institutional Review Board or Ethics Committee Address(es)
1101	Dr. Charles Byron Hicks		Dr. John Alexander Bartlett<br>Dr. Gary Matthew Cox<br>Ms. Kara McGee<br>Dr. Michael James Mugavero<br>Dr. Nathan Maclyn Thielman	Duke University Medical Center<br>Division of Infectious Diseases<br>Clinic 2J, Box 3284<br>Hospital South, Trent Drive<br>Durham, NC 27710<br>UNITED STATES	Duke University Health Systems<br>Institutional Review Board<br>Suite 9000, DUMC Box 2991<br>2400 Pratt Street<br>Durham, NC 27705<br>UNITED STATES
1102	Dr. Michael Alan Horberg		Dr. Alan Man	Kaiser Permanente Medical Center - Santa Clara<br>900 Kiely Boulevard<br>Santa Clara, CA 95051<br>UNITED STATES<br><br>Kaiser Permanente Medical Center - Santa Clara<br>Building 200, Suite 209 (drug shipment)<br>1333 Lawrence Expressway<br>Santa Clara, CA 95051<br>UNITED STATES	Kaiser Permanente Northern California IRB<br>Kaiser Foundation Research Institute<br>16th Floor<br>1800 Harrison Street<br>Oakland, CA 94612-3431<br>UNITED STATES

090177e1801b1594\Approved\Approved On: 24-Sep-2007 20:04

090177e1801b1594\Approved\Approved On: 24-Sep-2007 20:04

Center	Principal Investigator	Co-Investigator(s)	Sub-Investigator(s)	Address(es)	Institutional Review Board or Ethics Committee Address(es)
1103	Dr. Daniel Benjamin Klein		Dr. Susan Jane Jacobson	Kaiser Permanente<br>27400 Hesperian Boulevard<br>Hayward, CA 94545<br>UNITED STATES<br><br>Kaiser Permanente<br>Department of Infectious Diseases (Drug Shipment)<br>30116 Eigenbrodt Way<br>Union City, CA 94587<br>UNITED STATES	Kaiser Permanente Northern California IRB<br>Kaiser Foundation Research Institute<br>16th Floor<br>1800 Harrison Street<br>Oakland, CA 94612-3431<br>UNITED STATES
1104	Dr. Matthew Leibowitz<br>Dr. Steven Aloysius Miles (Previous PI)			UCLA Medical Center, William Reeve, PharmD<br>Drug Information Center<br>650 Charles E. Young Drive South<br>Los Angeles, CA 90095-6984<br>UNITED STATES	UCLA Office for the Protection of Research Subjects<br>Office for Protection of Research Subjects,<br>UCLA, Box 951694;<br>2107 Ueberroth Building,<br>Los Angeles, CA 90095-1694<br>UNITED STATES

Center	Principal Investigator	Co-Investigator(s)	Sub-Investigator(s)	Address(es)	Institutional Review Board or Ethics Committee Address(es)
1105	Dr. Daniel Skiest<br>Dr. Calvin Jay Cohen (Previous PI)<br>Dr. Claudia Martorell (Previous PI)<br>Dr. Anne Burnett Morris (Previous PI)		Ms. Carol A. Kane<br>Dr. Armando Paez<br>Frances Santiago<br>Ms. Myrna J. Schulte<br>Maribel Torano<br>Ms. Arlene Bermudez<br>Ms. Margarita Canuel<br>Dr. Amy E. Colson<br>Ms. Susan M. Cournoyer<br>Dr. Carlos Flores<br>Dr. St. John D. McGrath<br>Dr. Armando Paez<br>Frances Santiago<br>Dr. Harry Mark Schrager<br>Ms. Laurie F. Wojtusik	Community Research Initiative<br>Suite 31<br>780 Chestnut Street<br>Springfield, MA 01107<br>UNITED STATES	New England IRB<br>40 Washington Street, Ste 130<br>Wellesley, MA 02481<br>UNITED STATES
1106	Dr. Todd Stephen Wills<br>Dr. Jeffrey Philip Nadler (Previous PI)		Dr. Beata Casanas<br>Dr. Abbigail Chandler<br>Daniela M. Chiriboga Salazar<br>Dr. Juan Diaz<br>Dr. Douglas Allen Holt<br>Don Kurtyka ARNP<br>Dr. Anibal Maldonado<br>Dr. Javier Marinez<br>Dr. Jose Andres Montero<br>Yagneshvari Patel	Hillsborough County Health Department, Specialty Care Clinic<br>1105 East Kennedy Boulevard<br>Tampa, FL 33602<br>UNITED STATES	Florida Department of Health Institutional Review Board<br>4052 Bald Cypress Way Bin A-24<br>Tallahassee, FL 32399<br>UNITED STATES<br><br>University of South Florida Institutional Review Board<br>12901 Bruce D. Downs Blvd<br>MDC Box 35<br>Tampa, FL 33612

090177e1801b1594\Approved\Approved On: 24-Sep-2007 20:04

090177e1801b1594\Approved\Approved On: 24-Sep-2007 20:04

Center	Principal Investigator	Co-Investigator(s)	Sub-Investigator(s)	Address(es)	Institutional Review Board or Ethics Committee Address(es)
			Dr. Charurut Somboonwit<br>Chakrapol Sriaroon<br>Dr. Ana Velez<br>Terry L. Wilder<br>Dr. Aaron Cooks<br>Dr. Philbert Ford<br>Dr. Beata Herman<br>Dr. Patricia Leon<br>Dr. Igor Melnychuk<br>Dr. Michael Phillips<br>Dr. Shereen Saba<br>Sharon R. Weaver		UNITED STATES
1107	Dr. Paul Edward Sax		Dr. Cameron Ashbaugh<br>Dr. Lindsey Robert Baden<br>Joanne Delaney<br>Dr. Todd Ellerin<br>Jon Gothing<br>Dr. Daniel R. Kuritzkes<br>Dr. Rebeca M. Plank<br>William Theisen<br>Dr. Sigal Yawetz<br>Ms. Brianne Fitzgerald<br>Dr. Amy Garlin	Brigham and Women's Hospital<br>PBBA4<br>75 Francis Street<br>Boston, MA 02115<br>UNITED STATES	Partners Human Research Committee<br>Partners Human Research Committee<br>Suite 1002<br>116 Huntington Avenue<br>Boston, MA 02116<br>UNITED STATES

Center	Principal Investigator	Co-Investigator(s)	Sub-Investigator(s)	Address(es)	Institutional Review Board or Ethics Committee Address(es)
1108	Dr. Jack Thomas Stapleton		Kristine A. Davis<br>Ms. Julie K. Katseres<br>Dr. Jeffrey Meier<br>Ms. Barbara Ann Wiley	University of Iowa<br>Hospitals and Clinics<br>200 Hawkins Drive<br>Iowa City, IA 52242<br>UNITED STATES	Committee A Human Subjects Office<br>University of Iwoa<br>IRB-01<br>300 CMAB<br>Iowa City, IA 52242<br>UNITED STATES
1109	Dr. Roy Steigbigel		Sandra Brown<br>Dr. Lisa M. Chirch<br>Wayne Patterson<br>Frank Albergo	University Hospital, SUNY at Stony Brook<br>Drug Shipment<br>Pharmacy Dept, L1 Room 1-841<br>Health Science Drive<br>Stony Brook, NY 11794-7310<br>UNITED STATES<br><br>University Hospital, State University of New York at Stony Brook<br>Health Science Center<br>T15, 15-080<br>Stony Brook, NY 11794-8153<br>UNITED STATES	State University of New York at Stony Brook<br>Committee on Research Involving Human Subjects (CORIHS)<br>Research Administration,<br>West Melville Library, 5th Floor<br>Stony Brook, NY 11794-3368<br>UNITED STATES

090177e1801b1594\Approved\Approved On: 24-Sep-2007 20:04

Center	Principal Investigator	Co-Investigator(s)	Sub-Investigator(s)	Address(es)	Institutional Review Board or Ethics Committee Address(es)
1110	David Asmuth		Dr. Donna M. DeFreitas<br>Dr. Gregory Paul Melcher<br>Melissa A. Schreiber<br>Dr. Javeed Siddiqui<br>Dr. Paolo V. Troia-Cancio	ACTU located at CARES<br>1500 21st Street<br>Sacramento, CA 95814<br>UNITED STATES<br><br>University of California Davis, Medical Center (Drug Shipment)<br>Investigational Pharmacy<br>2315 Stockton Boulevard<br>Sacramento, CA 95817<br>UNITED STATES	University of California, Davis School of Medicine Human Subjects Review Committee<br>Ambulatory Care Clinic Building, Suite 3870<br>4860 Y Street<br>Sacramento, CA 95817<br>UNITED STATES
1111	Dr. Jason Mark Leider		Dr. Jacobo Abadi<br>Tracey Barnett<br>Dr. Fernando C. Carnavali<br>Mindy A. Golatt<br>Dr. Julie Hoffman<br>Dr. Elizabeth Robin Jenny-Avital<br>Dr. Mindy Jill Katz<br>Maura Porricolo<br>Dr. Michael George Rosenberg	Jacobi Medical Center<br>1400 Pelham Parkway South<br>Bronx, NY 10461<br>UNITED STATES	Biomedical Research Alliance of New York, LLC<br>Suite 100<br>225 Community Drive<br>Great Neck, NY 11021<br>UNITED STATES

090177e1801b1594\Approved\Approved On: 24-Sep-2007 20:04

Center	Principal Investigator	Co-Investigator(s)	Sub-Investigator(s)	Address(es)	Institutional Review Board or Ethics Committee Address(es)
1119	Dr. Jason Andrew Flamm		Sue Elam	Kaiser Hospital<br>1st Floor Pharmacy (drug shipment)<br>2025 Morse Avenue<br>Sacramento, CA 95825<br>UNITED STATES<br><br>Kaiser Hospital<br>2025 Morse Avenue<br>Sacramento, CA 95825<br>UNITED STATES	Kaiser Permanente Northern California IRB<br>Kaiser Foundation Research Institute<br>16th Floor<br>1800 Harrison Street<br>Oakland, CA 94612-3431<br>UNITED STATES
1120	Dr Jorge E. Rodriguez		Dr. R. Derrick Knowles	Orange Coast Medical Group<br>Suite 126<br>361 Hospital Road<br>Newport Beach, CA 92663<br>UNITED STATES	Schulman Associates IRB, Inc.<br>4290 Glendale-Milford Road<br>Cincinnati, OH 45242<br>UNITED STATES

* Did not randomize subjects

090177e1801b1594\Approved\Approved On: 24-Sep-2007 20:04

090177e1801b1594\Approved\Approved On: 24-Sep-2007 20:04

Center	Principal Investigator	Co-Investigator(s)	Sub-Investigator(s)	Address(es)	Institutional Review Board or Ethics Committee Address(es)
1121	Dr. Javier Osvaldo Morales-Ramirez		Dr. Glyced Flores-De-Jesus<br>Dr. Orlando A. Lopez Santiago<br>Dr. Efrain Rodriguez-Vigil<br>Dr. Sonia E. Velazquez Navarro	Centro Mas Salud Dr. Gualberto Rabell<br>900 Cerra St., Corner Hoare<br>Stop 15, San Juan, PR 00909<br>UNITED STATES<br><br>Clinical Research Puerto Rico, Incorporated<br>Suite 501<br>359 De Diego Avenue<br>San Juan, PR 00909<br>UNITED STATES	Schulman Associates IRB, Inc.<br>4290 Glendale-Milford Road<br>Cincinnati, OH 45242<br>UNITED STATES
1122 *	Dr. Gladys E. Sepulveda-Arzola		Dr. Rafael O. Mendoza-Rodriguez	Intitutio de Investigacion Cientifica / Anexo Hospital Oncologico<br>Anexo Hospital Oncologico<br>Office #6<br>917 TiTo Castro Avenue<br>Ponce, PR 00731<br>UNITED STATES	Schulman Associates Institutional Review Board, Inc.<br>4290 Glendale-Milford<br>Cincinnati, OH 45242<br>UNITED STATES

* Did not randomize subjects

Center	Principal Investigator	Co-Investigator(s)	Sub-Investigator(s)	Address(es)	Institutional Review Board or Ethics Committee Address(es)
1124	Dr. Carmen D. Zorrilla		Dr. Lorna L. De Jesus Cartagena MD<br>Dr. Santiago Marrero de Leon<br>Dr. Olga I. Mendez Flores<br>Vivian M. Tamayo Agrait<br>Dr. Maribel Acevedo MD	University of Puerto Rico, School of Medicine<br>Gamma Project, Cardiovascular Center<br>8th Floor, Pharmacy Room<br>San Juan, PR 00935<br>UNITED STATES<br><br>University of Puerto Rico/Maternal Infant Studies Center (CEMI)<br>Biomedical Building II/First Floor<br>Rio Piedras, PR 00935<br>UNITED STATES	University of Puerto Rico<br>Medical Sciences Campus<br>PO Box 365067<br>San Juan, PR 00936-5067<br>UNITED STATES
1127	Dr. Judith E. Feinberg		Dr. Carl Jack Fichtenbaum<br>Dr. Peter Alan Grubbs<br>Dr. Pamposh Darbari Kaul<br>Dr. Jaime Robertson	University of Cincinnati Medical Center<br>Holmes Division - Mail Location 0405<br>Eden Avenue and Albert Sabin Way<br>Cincinnati, OH 45267-0405<br>UNITED STATES	University of Cincinnati Medical Center Institutional Review Board<br>G08 Wherry Hall<br>Eden Avenue<br>Cincinnati, OH 45267-0567<br>UNITED STATES

090177e1801b1594\Approved\Approved On: 24-Sep-2007 20:04

Center	Principal Investigator	Co-Investigator(s)	Sub-Investigator(s)	Address(es)	Institutional Review Board or Ethics Committee Address(es)
1128	Dr. Alfred F. Burnside Jr.			The Burnside Clinic<br>14 Calendar Court<br>Columbia, SC 29206<br>UNITED STATES	Schulman Associates Institutional Review Board, Inc.<br>4290 Glendale-Milford<br>Cincinnati, OH 45242<br>UNITED STATES
1129	Dr. Thomas B. Campbell		Cathi Basler<br>Dr. Monica Lynn Carten<br>Dr. Elizabeth Connick<br>Dr. John George Gerber<br>Dr. Steven Constant Johnson<br>Dr. John R. Koeppe<br>Sarah C. Lammers<br>Dr. Marilyn Eckstein Levi<br>Dr. Kenneth Allen Lichtenstein<br>Dr. Nancy E. Madinger<br>Beverly A. Putnam<br>Mary Graham Ray<br>James A. Scott	University of Colorado Health Sciences Center/University of Colorado Hospital<br>4200 East Ninth Avenue<br>Denver, CO 80262<br>UNITED STATES	Colorado Multiple Institutional Review Board<br>Building 500, Room E2353<br>13001 East 17th Place<br>Aurora, CO 80010-7238<br>UNITED STATES
1130	Dr. Lucia M. Martinez-Bejar<br>Dr. Lucia M. Martinez-Bejar (Previous PI)		Dr. Lemuel Aigbivbalu<br>Dr. Ana Alvarez<br>Bernadette Belgado<br>Dr. Jeri Dyson<br>Carol Fulton	Shands/Jacksonville<br>Department of Pharmacy<br>655 West 8th Street<br>Jacksonvile, FL 32209<br>UNITED STATES	Western IRB<br>3535 Seventh Avenue, SW<br>Olympia, WA 98502<br>UNITED STATES

090177e1801b1594\Approved\Approved On: 24-Sep-2007 20:04

090177e1801b1594\Approved\Approved On: 24-Sep-2007 20:04

Center	Principal Investigator	Co-Investigator(s)	Sub-Investigator(s)	Address(es)	Institutional Review Board or Ethics Committee Address(es)
	Dr. Angela Xavier (Previous PI)		Dr. Sabiha Hussain<br>Dr. Saniyyah I. Mahmoudi<br>Dr. June M. McAdams<br>Dr. Ayesha Mirza<br>Elaine Poon<br>Dr. Mobeen Hasan Rathore<br>Ms. Sandra Taylor<br>Dr. Motasem A. Abuelreish<br>Dr. Denise Darracott<br>Dr. Eiman El-Sayed<br>Dr. Ivan Guerrero<br>Dr. Abeer Khayat<br>Dr. Jonathan A. Schneider	University of Florida, Jacksonville<br>Rainbow Center<br>655 West 8th Street<br>Jacksonville, FL 32209<br>UNITED STATES	
1131	Dr. Todd Allen Fralich		Dr. Jennifer Bartczak	North Point Medical<br>Suite 205<br>6405 North Federal Highway<br>Fort Lauderdale, FL 33308<br>UNITED STATES	Schulman Associates IRB, Inc.<br>4290 Glendale-Milford Road<br>Cincinnati, OH 45242<br>UNITED STATES

* Did not randomize subjects

Center	Principal Investigator	Co-Investigator(s)	Sub-Investigator(s)	Address(es)	Institutional Review Board or Ethics Committee Address(es)
1132 *	Dr. Karam Chucri Mounzer		Cecile Gallo<br>Dr. Jay Robert Kostman<br>Joseph Patrick Ondercin<br>Ms. Elizabeth Schmidt<br>Dr. Sofia Sherman-Weber<br>Sarah Smith	Philadelphia Fight<br>5th Floor<br>1233 Locust Street<br>Philadelphia, PA 19107<br>UNITED STATES	Philadelphia Fight Institutional Review Board<br>1233 Locust Street<br>5th Floor<br>Philadelphia, PA 19107<br>UNITED STATES
1133	Dr. Michael Grant Sension		Dr. Kathleen K. Graham<br>Dr. Sheetal Sharma	North Broward Hospital District<br>Comprehensive Care Center<br>1101 NW First Street<br>Ft. Lauderdale, FL 33311<br>UNITED STATES	North Broward Hospital District IRE<br>1600 S. Andrews Ave.<br>Ft. Lauderdale, FL 33311<br>UNITED STATES
1135	Dr. Allan Rowan Kelly			Kelly, Allan Rowman MD<br>929 College Ave.<br>Fort Worth, TX 76104-3048<br>UNITED STATES	Schulman Associates IRB, Inc.<br>4290 Glendale-Milford Road<br>Cincinnati, OH 45242<br>UNITED STATES
1139 *	Dr. Robert P. Smith Jr.		Sandra T. Putnam	Maine Medical Center / Virology Treatment Center<br>48 Gilman Street<br>Portland, ME 04102<br>UNITED STATES	Maine Medical Center<br>IRB Office<br>80 Research Drive<br>Scarborough, ME 04074<br>UNITED STATES

* Did not randomize subjects

090177e1801b1594\Approved\Approved On: 24-Sep-2007 20:04

Center	Principal Investigator	Co-Investigator(s)	Sub-Investigator(s)	Address(es)	Institutional Review Board or Ethics Committee Address(es)
1141	Dr. Paula Rosa Greiger-Zanlungo		Dr. Debra Spicehandler	Greiger Clinic<br>Room 501<br>12 North 7th Avenue<br>Mount Vernon, NY 10550<br>UNITED STATES	Schulman Associates Institutional Review Board, Inc.<br>4290 Glendale-Milford Road<br>Cincinnati, OH 45242<br>UNITED STATES<br><br>Schulman Associates Institutional Review Board, Incorporated<br>4290 Glendale - Milford Road<br>Cincinnati, OH 45242<br>UNITED STATES

090177e1801b1594\Approved\Approved On: 24-Sep-2007 20:04

090177e1801b1594\Approved\Approved On: 24-Sep-2007 20:04

Center	Principal Investigator	Co-Investigator(s)	Sub-Investigator(s)	Address(es)	Institutional Review Board or Ethics Committee Address(es)
1144	Dr. George Ghazaros Burnazian		Dr. Benjamin L. Portnoy	Administrative Research Site (Global)<br>Global Clinical Trial Link, LLC<br>430 Lorna Square<br>Birmingham, AL 35216<br>UNITED STATES<br><br>Administrative Research Site (Local)<br>Global Clinical Trial Link, LLC<br>7700 Main Street, 2nd Floor<br>Houston, TX 77030<br>UNITED STATES<br><br>Office of Dr. George Burnazian<br>Suite 530<br>1200 Binz<br>Houston, TX 77004<br>UNITED STATES	Schulman Associates IRB, Inc.<br>4290 Glendale-Milford Road<br>Cincinnati, OH 45242<br>UNITED STATES
1146 *	Dr. Anthony LaMarca		Dr. Denise LaMarca<br>Dr. Raymond Seales	Therafirst Medical Centers<br>4011 North Federal Highway<br>Ft. Lauderdale, FL 33308<br>UNITED STATES	Schulman Associates IRB, Inc.<br>4290 Glendale-Milford Road<br>Cincinnati, OH 45242<br>UNITED STATES

* Did not randomize subjects

Center	Principal Investigator	Co-Investigator(s)	Sub-Investigator(s)	Address(es)	Institutional Review Board or Ethics Committee Address(es)
1147	Dr. Kathleen King Casey		Dr. Nasir Ahmad<br>Dr. Jose Mario Fune<br>Dr. Nancy J. Kruvant-Gornish<br>Dr. Edward Liu	Jersey Shore Medical Center<br>71 Davis Avenue<br>Neptune, NJ 07753<br>UNITED STATES	Jersey Shore University Medical Center, IRB<br>1945 Route 33<br>Neptune, NJ 07754<br>UNITED STATES<br><br>Western Institutional Review Board<br>P.O. Box 12029<br>3535 Seventh Avenue Southwest<br>Olympia, WA 98508-2029<br>UNITED STATES
1148	Dr. David Wright		Dr. Mary E. Bartz<br>Dr. Cynthia Curll Brinson<br>Dr. Michael E. Killian<br>Dr. Gary L. Werntz	Central Texas Clinical Research<br>Suite 302<br>900 East 30th Street<br>Austin, TX 78705<br>UNITED STATES	Schulman Associates IRB, Inc.<br>4290 Glendale-Milford Road<br>Cincinnati, OH 45242<br>UNITED STATES

* Did not randomize subjects

090177e1801b1594\Approved\Approved On: 24-Sep-2007 20:04
