# Supplementary material for: A Genotypic Test for HIV-1 Tropism Combining Sanger Sequencing with Ultradeep Sequencing Predicts Virologic Response in Treatment-Experienced Patients
Source: PLoS One. 2012 Sep 27;7(9):e46334. doi: 10.1371/journal.pone.0046334 (PMC3459909; doi:10.1371/journal.pone.0046334)
Supplement: Supporting Information S4 — List of investigators and corresponding ethics committees or Institutional review boards for study A4001028 (MOTIVATE-2). (PDF) [file pone.0046334.s004.pdf]

A4 LIST OF INVESTIGATORS AND CORRESPONDING ETHICS COMMITTEES OR INSTITUTIONAL REVIEW BOARDS

Australia

Coordinating Investigators:
<None Entered>

Center	Principal Investigator	Co-Investigator(s)	Sub-Investigator(s)	Address(es)	Institutional Review Board or Ethics Committee Address(es)
1005	Prof. David Cooper		Kathy M. Barnes<br>Prof. Bruce J. Brew<br>Dr. Alexandra Calmy<br>Assoc. Prof. Andrew Carr<br>Dr. Robert J. Fielden<br>Dr. Elizabeth Hamlyn<br>Dr. Kersten Koelsch<br>Dr. Patrick Mallon<br>Prof. Deborah J. E. Marriott<br>Richard J. Norris<br>Dr. Sarah Pett<br>Martina Rafferty<br>Dr. Christopher J. Weatherall<br>Dr. Winnie Yin Tong<br>Dr. Alan Winston	National Centre in HIV Epidemiology and Clinical Research<br>Level 2, Medical Centre<br>376 Victoria Street<br>Darlinghurst, NSW 2010<br>AUSTRALIA	St. Vincents Hospital<br>Research Ethics Committee<br>Level 15<br>Victoria Street<br>Darlington, NSW 2010<br>AUSTRALIA
1020	A/Prof. Jennifer Frances Hoy		Sally J. Algar<br>Ms. Mellissa J. Bryant<br>Dr. Luke F. Chen<br>Catherine Cherry<br>Teresa Girke<br>Dr. Sophie A. Herbert<br>Ms. Karen L. Hutchinson<br>Dr. Stephen J. Kent<br>Dr. Richard J. Moore<br>Janine J. Roney<br>Dr. Jessica Rotty<br>Alan Street<br>Dr. Steven L. Wesselingh	The Alfred Hospital<br>Infectious Diseases Unit, Clinical Research Section<br>PO Box 315<br>Melbourne, VICTORIA 3004<br>AUSTRALIA	Alfred Hospital<br>Alfred Hospital<br>Commercial Raod<br>Melbourne, Victoria 3004<br>AUSTRALIA

090177e1801b151d\Approved\Approved On: 03-Oct-2007 20:09

090177e1801b151d\Approved\Approved On: 03-Oct-2007 20:09

Center	Principal Investigator	Co-Investigator(s)	Sub-Investigator(s)	Address(es)	Institutional Review Board or Ethics Committee Address(es)
1021	Dr. Richard J. Moore		Dr. Jonathan S. C. Anderson<br>Paul Brian Cortissos<br>Dr. Ching Thai Lim<br>Kaye E. Lowe<br>Julia S. Pearce<br>Dr. Jeffrey I. Willcox	The Carlton Clinic Pty Ltd<br>88 Rathdowne Street<br>Carlton, VICTORIA 3053<br>AUSTRALIA	Royal Australian College of General Practitioners National Research and Evaluation Ethics Committee<br>1 Palmerston Crescent<br>SOUTH MELBOURNE, VIC 3205<br>AUSTRALIA
1022	Dr. Dominic E. Dwyer		Dr. Deborah Couldwell<br>Dr. Nicole Gilroy<br>Dr. Jon Iredell<br>Claire Kesby<br>Dr. Donald Packman<br>Margaret Piper<br>Assoc. Prof. Graeme Stewart	Westmead Hospital<br>Department of Virology, CIDMLS, ICPMR<br>PO Box 533<br>Wentworthville, NSW 2145<br>AUSTRALIA	Western Sydney Area Health Service Human Research Ethics Committee<br>Westmead Hospital<br>Cnr Hawkesbury & Darcy Roads<br>Westmead, NSW 2145<br>AUSTRALIA
1027	Assoc. Prof. Anthony M. Allworth		Holly Asher<br>Natalie Lara Gerns<br>Dr. Mark Denis Kelly<br>Dr. Emma S. McBryde<br>Assoc. Prof. James S. McCarthy<br>Anne Maree Sleat<br>Assoc. Prof. Marion L. Woods<br>Janelle Zillmann	Infectious Diseases Unit<br>6th Floor Joyce Tweddell Building<br>Royal Brisbane Hospital<br>Herston Road<br>Herston, QUEENSLAND 4029<br>AUSTRALIA	Royal Brisbane & Womens Hospital Health ServiceDistrict Office of the Human Research Ethics Committe<br>Herston Road<br>Herston , QLD, Australia 4029<br>AUSTRALIA
1028	Dr. Mark T. Bloch		Shikha Agrawal<br>Colin Anderson<br>Dr. David R. Austin<br>Dr. Kate Bessey<br>Caroline E. Egan<br>Wilma P. Goodyear<br>Dr. Andrew Gowers<br>Peter Grzonkowski<br>Dr. Hsin-Hua H. Liu<br>Samantha Miller<br>Dr. Ercel Ozser<br>Dr. Dick C. Quan<br>Ruth Hutchinson	Holdsworth House General Practice<br>Suite 1<br>32A Oxford Street<br>Darlinghurst, 2010<br>AUSTRALIA	South Eastern Sydney Area Health Service (Eastern Section) Research Ethics Committee<br>Room G71, Edmund Blacket Building<br>Cnr High & Avoca Sts<br>Randwick, NSW 2031<br>AUSTRALIA

* Did not randomize subjects

Center	Principal Investigator	Co-Investigator(s)	Sub-Investigator(s)	Address(es)	Institutional Review Board or Ethics Committee Address(es)
1029	Dr. John Chuah		Dr. Stuart Aitken<br>Fiona Clark<br>Brenda Henry<br>Denise Lester<br>Ngaire Wendt	Gold Coast Sexual Health Clinic<br>2019 Gold Coast Highway<br>Miami, Queensland 4220<br>AUSTRALIA	Gold Coast Health Services Human Research Ethics Committee<br>108 Nerang Street<br>SOUTHPORT, QLD 4215<br>AUSTRALIA
1030	Prof. Julian Gold		Dr. Timothy G. Barnes<br>Dr. Derek J. Chan<br>Mr. Robert Cherry<br>Dr. Catherine Ealing<br>Margaret Ewing<br>Dr. Vanessa Farr<br>Dr. Virginia L. Furner<br>Mr. Jason Gao<br>Dr. Henry Mackellar Michelmore<br>Richard Osborne<br>Dr. Jeffrey J. Post<br>Mr. Anthony Price<br>Jeganathan Sarangapany<br>A/Prof. Don E. Smith<br>Grant Sweeney	Albion Street Centre<br>150 - 154 Albion Street<br>Sydney, NSW 2010<br>AUSTRALIA	South Eastern Sydney Area Health Service (Eastern Section) Research Ethics Committee<br>Room G71, Edmund Blacket Building<br>Cnr High & Avoca Sts<br>Randwick, NSW 2031<br>AUSTRALIA
1031 *	Dr. Norman J. Roth		Dr. Mark Chong<br>Dr. Beng H. Eu<br>Dr. Michael J. Porter<br>Dr. Sven A. Strecker<br>Helen P. Wood	Prahan Market Clinic<br>131 Commercial Road<br>South Yarra, VICTORIA 3141<br>AUSTRALIA	Royal Australian College of General Practitioners National Research and Evaluation Ethics Committee<br>1 Palmerston Crescent<br>SOUTH MELBOURNE, VIC 3205<br>AUSTRALIA
1086	Dr. Neil J. Bodsworth		Dr. Linda S. Dayan<br>Sophie E. Dinning<br>Dr. Robert J. Finlayson<br>Wilma P. Goodyear<br>Dr. Catherine M. Pell<br>Dr. Ross S. Price<br>Robyn A. Richardson<br>Dr. Emanuel G. Vlahakis	Taylor Square Private Clinic<br>393 Bourke Street<br>Surry Hills, NSW 2010<br>AUSTRALIA	South Eastern Sydney Area Health Service (Eastern Section) Research Ethics Committee<br>Room G71, Edmund Blacket Building<br>Cnr High & Avoca Sts<br>Randwick, NSW 2031<br>AUSTRALIA

* Did not randomize subjects

090177e1801b151d\Approved\Approved On: 03-Oct-2007 20:09

Center	Principal Investigator	Co-Investigator(s)	Sub-Investigator(s)	Address(es)	Institutional Review Board or Ethics Committee Address(es)
1120	Dr. Cassy Workman		Dr. Kristyn Chantal Adolphe<br>Dr. Robert J. Fielden<br>Dr. Mark Denis Kelly<br>Dr. Catriona J. Ooi<br>Vanessa Rees	AIDS Research Initiative<br>48 Little Oxford Street<br>Darlinghurst, NSW 2010<br>AUSTRALIA	South Eastern Sydney Area Health Service (Eastern Section) Research Ethics Committee<br>Cnr High & Avoca Street<br>Randwick, NSW, Australia 2031<br>AUSTRALIA
1123	Dr. Nicholas Doong		Jeffrey H. Hudson	Dr. Doong's Surgery<br>8 Burwood Road<br>Burwood, NSW 2134<br>AUSTRALIA	Royal Australian College of General Practitioners National Research and Evaluation Ethics Committee<br>1 Palmerston Crescent<br>SOUTH MELBOURNE, VIC 3205<br>AUSTRALIA

090177e1801b151d\Approved\Approved On: 03-Oct-2007 20:09

090177e1801b151d\Approved\Approved On: 03-Oct-2007 20:09

Belgium

Coordinating Investigators:
Patricia Dellot

Center	Principal Investigator	Co-Investigator(s)	Sub-Investigator(s)	Address(es)	Institutional Review Board or Ethics Committee Address(es)
1009	Dr. Michel Moutschen		Dr. Jean Demonty<br>Dr. Frederic Frippiat<br>Dr. Philippe Leonard<br>Dr. Francoise Uurlings<br>Patricia Dellot	C.H.U. Sart-Tilman<br>Maladies Infectieuses<br>Domaine du Sart-Tilman B35<br>Liege, 4000<br>BELGIUM	Comite Local d'Ethique Hospitalier<br>CHU St Pierre/UMC St Pieter<br>Batiment Direction<br>Rue Haute/Hoogstraat 322<br>Bruxelles, 1000<br>BELGIUM<br><br>Comite d'Ethique de la Faculte de Medecine<br>Universite de Liege<br>Service de Pharmacologie B23<br>Liege, 4000<br>BELGIUM
1032 *	Prof. Dirk Vogelaers		Marleen Bogaert<br>Yolanda Pelgrom<br>Beatrijs Van der Gucht<br>Dr. Filip Van Wanzeele	U.Z. Gent<br>De Pintelaan 125<br>Gent, 9000<br>BELGIUM	Comite Local d'Ethique Hospitalier<br>CHU St Pierre/UMC St Pieter<br>Batiment Direction<br>Rue Haute/Hoogstraat 322<br>Bruxelles, 1000<br>BELGIUM<br><br>Ethisch Comite UZ Gent<br>P/a Heymans Instituut<br>UZ Gent /2P4<br>De Pintelaan<br>Gent, 9000<br>BELGIUM

* Did not randomize subjects

Center	Principal Investigator	Co-Investigator(s)	Sub-Investigator(s)	Address(es)	Institutional Review Board or Ethics Committee Address(es)
1033	Dr. Bernard C. Vandercam		Angeline Henry<br>Dr. Anne Vincent<br>Dr. Jean-Cyr Yombi	Cliniques Universitaires St-Luc<br>Maladies infectieuses<br>Avenue Hippocrate 10<br>Brussels, 1200<br>BELGIUM	Comission d'Ethique Biomedical Hospitalo-Facultaire<br>Prof. Dr. Maloteaux<br>Avenue Hippocrate 55.14<br>Bruxelles, 1200<br>BELGIUM<br><br>Comite Local d'Ethique Hospitalier<br>CHU St Pierre/UMC St Pieter<br>Batiment Direction<br>Rue Haute/Hoogstraat 322<br>Bruxelles, 1000<br>BELGIUM
1040	Dr. Jean Christophe Goffard		Dr. Michel De Cock<br>Dr. Claire Michele Farber<br>Prof. Jean-Paul Van Vooren	Hospital Erasme<br>Route de Lennik 808<br>Brussels, 1070<br>BELGIUM	Comite Local d'Ethique Hospitalier<br>CHU St Pierre/UMC St Pieter<br>Batiment Direction<br>Rue Haute/Hoogstraat 322<br>Bruxelles, 1000<br>BELGIUM<br><br>Comite d'Ethique de la Faculte de medecine de l'ULB<br>CP596<br>Route de Lennick 808<br>Bruxelles, 1070<br>BELGIUM
1118	Prof. Nathan Clumeck		Dr. Stephane De Wit<br>Dr. Kabamba Kabeya<br>Elisabeth O'Doherty	C.H.U. St-Pierre<br>Clinique des Maladies Infectieuses<br>Rue Haute, 322 (PL5)<br>Brussels, 1000<br>BELGIUM	Comite Local d'Ethique Hospitalier<br>CHU St Pierre/UMC St Pieter<br>Batiment Direction<br>Rue Haute/Hoogstraat 322<br>Bruxelles, 1000<br>BELGIUM

* Did not randomize subjects

090177e1801b151d\Approved\Approved On: 03-Oct-2007 20:09

Canada

Coordinating Investigators:
<None Entered>

Center	Principal Investigator	Co-Investigator(s)	Sub-Investigator(s)	Address(es)	Institutional Review Board or Ethics Committee Address(es)
1236 *	Dr. Danielle Rouleau		Dr. Alexandra de Pokomandy<br>Dr. Claude Fortin	Hopital Notre-Dame du CHUM<br>1560 Sherbrooke Est<br>Montreal, Quebec H2L 4M1<br>CANADA<br><br>Hopital Notre-Dame du CHUM<br>Pavillon Louis-Charles Simard<br>10 etage, UHRESS, Z10904<br>2065 Alexandre de Sève<br>Montreal, Quebec H2L 2W5<br>CANADA	Comite d'ethique de la recherche du CHUM (Hopital Notre-Dame)<br>Edifice Cooper<br>Mezzanine 2<br>3981 boul. St-Laurent<br>Montreal, QC H2W 1Y5<br>CANADA

* Did not randomize subjects

090177e1801b151d\Approved\Approved On: 03-Oct-2007 20:09

France

Coordinating Investigators:
<None Entered>

Center	Principal Investigator	Co-Investigator(s)	Sub-Investigator(s)	Address(es)	Institutional Review Board or Ethics Committee Address(es)
1006	Prof. Francois Raffi		Dr. Clotilde Allavena<br>Dr. Eric Billaud<br>Dr. Benedicte Bonnet<br>Dr. David Boutoille<br>Dr. Cecile Brunet-Francois<br>Dr. Veronique Gagey<br>Dr. Bridgette Milpied<br>Dr. Pascale Morineau le Houssine<br>Dr. Veronique Reliquet-Guesnier	CHU de Nantes - Hopital Hotel Dieu<br>Service de Médicine Interne<br>1 place Alexis Ricordeau<br>Nantes, 44093<br>FRANCE	CCPPRB No. 2 des Pays de la Loire<br>Immeuble Deurbroucq<br>5, allees de I'lle Gloriette<br>Nantes, Cedex 01 44093<br>FRANCE
1008 *	Dr. Isabelle Poizot Martin		Dr. Marie-Pierre Drogoul-Vey<br>Dr. Veronique Frixon-Marin	Hopital Sainte Marguerite<br>Hopital de Jour CISIH<br>270 Boulevard de Sainte Marguerite<br>Marseille, Cedex 09 13274<br>FRANCE	CCPPRB No. 2 des Pays de la Loire<br>Immeuble Deurbroucq<br>5, allees de I'lle Gloriette<br>Nantes, Cedex 01 44093<br>FRANCE
1034	Dr. Michele Bentata		Dr. Radia Djebbar Bekkouche<br>Dr. Rachid Mansouri<br>Dr. Francois Rouges	Hopital Avicenne<br>Service de Médicine Interne<br>125 rue de Stalingrad<br>Bobigny, Cedex 93009<br>FRANCE	CCPPRB No. 2 des Pays de la Loire<br>Immeuble Deurbroucq<br>5, allees de I'lle Gloriette<br>Nantes, Cedex 01 44093<br>FRANCE
1035	Dr. Jean-Michel Molina		Dr. Suna Balkan<br>Dr. Nathalie De Castro<br>Dr. Nathalie Colin de Verdiere<br>Dr. Samuel Ferret<br>Dr. Sandra Fournier<br>Dr. Andre Furco-Mazzantini<br>Dr. Jerome Goguel<br>Dr. Henri-Frederic Guyon<br>Dr. Matthieu Lafaurie	Hospital St-Louis<br>Service des Maladies Infectieuses<br>1 Avenue Claude Vellefaux<br>Paris, Cedex 10 75475<br>FRANCE	CCPPRB No. 2 des Pays de la Loire<br>Immeuble Deurbroucq<br>5, allees de I'lle Gloriette<br>Nantes, Cedex 01 44093<br>FRANCE

* Did not randomize subjects

090177e1801b151d\Approved\Approved On: 03-Oct-2007 20:09

090177e1801b151d\Approved\Approved On: 03-Oct-2007 20:09

Center	Principal Investigator	Co-Investigator(s)	Sub-Investigator(s)	Address(es)	Institutional Review Board or Ethics Committee Address(es)
			Dr. Marie Lagrange-Xelot<br>Dr. Juliette Pavie<br>Dr. Diane Ponscarme<br>Dr. Anne Rachline<br>Marthe Rigal<br>Prof. Willy Rozenbaum<br>Dr. Muriel Tourneur		
1036	Prof. Pierre-Marie Girard		Dr. Ana-Maria Begle<br>Dr. Diane Bollens<br>Dr. Benedicte Lefebvre<br>Dr. Zineb Ouazene	Hopital Saint Antoine<br>Service des Maladies Infectieuses<br>184 rue du Faubourg Saint Antoine<br>Paris, Cedex 12 75571<br>FRANCE	CCPPRB No. 2 des Pays de la Loire<br>Immeuble Deurbroucq- 5, allee de l'ile Gloriette<br>44093 Nantes Cedex 01<br>FRANCE
1037	Dr. Laurent Hocqueloux		Dr. Mohamadou Niang<br>Dr. Thierry Prazuck	CHR d'Orleans la Source<br>Service des Maladies Infectieuses<br>Avenue de l'Hopital<br>BP 6709<br>Orleans, Cedex 02 45067<br>FRANCE	CCPPRB No. 2 des Pays de la Loire<br>Immeuble Deurbroucq<br>5, allees de I'lle Gloriette<br>Nantes, Cedex 01 44093<br>FRANCE
1038	Dr. Renaud Verdon		Dr. Claude Bazin<br>Dr. Sylvie Dargere<br>Anne Martin<br>Dr. Vincent Noyon<br>Dr. Jean-Jaques Parienti<br>Rene Zebekolo	Centre Hospitalier Universitaire de Caen<br>Unité des Maladies Infectieuses<br>Avenue de la Côte de Nacre<br>Caen, Cedex 14033<br>FRANCE	CCPPRB No. 2 des Pays de la Loire<br>Immeuble Deurbroucq<br>5, allees de I'lle Gloriette<br>Nantes, Cedex 01 44093<br>FRANCE
1087 *	Dr. Alain Lafeuillade		Dr. Antoine Cheret<br>Dr. Gilles Hittinger	Hopital Chalucet<br>Unite d'Infectologie<br>Rue Chalucet<br>Toulon, BP1412 83056<br>FRANCE	CCPPRB No. 2 des Pays de la Loire<br>Immeuble Deurbroucq- 5, allee de l'ile Gloriette<br>44093 Nantes Cedex 01<br>FRANCE

* Did not randomize subjects

Center	Principal Investigator	Co-Investigator(s)	Sub-Investigator(s)	Address(es)	Institutional Review Board or Ethics Committee Address(es)
1088	Prof. Jacques Reynes		Dr. Nadine Atoui<br>Dr. Vincent Baillat<br>Dr. Carine Favier<br>Dr. Vincent Le Moing<br>Dr. Corinne Merle<br>Elisabeth Picou<br>Dr. Martine Siffert	Hopital Gui de Chauliac<br>Service des Maladies Infectieuses<br>80 avenue Augustin Fliche<br>Montpellier, 34295<br>FRANCE	CCPPRB No. 2 des Pays de la Loire<br>Immeuble Deurbroucq- 5, allee de l'ile Gloriette<br>44093 Nantes Cedex 01<br>FRANCE
1089	Dr. Gilles Pialoux		Dr. Catherine Chakvetadze<br>Dr. Tuna Lukiana<br>Dr. Anaenza Maresca<br>Dr. Laurence Slama	Hopital Tenon, Service des Maladies Infectieuses<br>4 Rue de la Chine<br>Paris, 75, 75020<br>FRANCE	CCPPRB No. 2 des Pays de la Loire<br>Immeuble Deurbroucq- 5, allee de l'ile Gloriette<br>44093 Nantes Cedex 01<br>FRANCE
1091	Dr. Roland Landman		Dr. Isabelle Fournier<br>Dr. Pierre Klutse<br>Dr. Golriz Pahlavan<br>Dr. Bao Phung<br>Dr. Pascal Ralaimazava	Hopital Bichat-Claude Bernard, Service des Maladies Infectieuses<br>46 rue Henri Huchard<br>Paris, 75018<br>FRANCE	CCPPRB No. 2 des Pays de la Loire<br>Immeuble Deurbroucq<br>5, allees de I'lle Gloriette<br>Nantes, Cedex 01 44093<br>FRANCE
1111 *	Prof. Jean-Francois Delfraissy		Dr. Christelle Chantalat<br>Dr. Marc De Lavaissiere<br>Dr. Martin Duracinsky<br>Dr. Cecile Goujard<br>Dr. Marie-Stephane N'Guessan<br>Dr. Delphine Peretti<br>Dr. Yann Quertainmont	Hopital de Bicêtre<br>Service de Médicine Interne, Maladies Infectieuses<br>78 Rue du Général Leclerc<br>Le Kremlin Bicêtre, 94270<br>FRANCE	CCPPRB No. 2 des Pays de la Loire<br>Immeuble Deurbroucq<br>5, allees de I'lle Gloriette<br>Nantes, Cedex 01 44093<br>FRANCE
1119	Dr. Laurent Cotte		Dr. Claude Augustin-Normand<br>Dr. Francois Bailly<br>Dr. Nadine Benmakhlouf<br>Dr. Valerie Gueripel<br>Dr. Bertrand Lebouche<br>Dr. Marianne Maynard-Muet<br>Dr. Patrick Miaihles<br>Dr. Sylvie Radenne<br>Dr. Isabelle Schlienger<br>Prof. Christian Trepo	Hopital Hotel Dieu Lyon<br>Service SIDA<br>1 Place de l'Hôpital<br>Lyon , Cedex 02 69288<br>FRANCE	CCPPRB No. 2 des Pays de la Loire<br>Immeuble Deurbroucq<br>5, allees de I'lle Gloriette<br>Nantes, Cedex 01 44093<br>FRANCE

* Did not randomize subjects

090177e1801b151d\Approved\Approved On: 03-Oct-2007 20:09

Center	Principal Investigator	Co-Investigator(s)	Sub-Investigator(s)	Address(es)	Institutional Review Board or Ethics Committee Address(es)
1122	Dr. Christophe Piketty		Dr. Dominigue Batisse<br>Dr. Martin Buisson<br>Dr. Philippe Castiel<br>Dr. Jean Derouineau<br>Dr. Gustavo Gonzalez-Canali<br>Dr. Karima Haddadi<br>Dr. Mufide Kahraman<br>Dr. Marina Karmochkine<br>Dr. Michel Kazatchkine<br>Dr. Aldo Abraham Trylesinski<br>Dr. Laurence Weiss	Hospital European George Pompidou<br>Service d'Immunologie Clinique<br>20 rue Leblanc<br>Paris, 75015<br>FRANCE	CCPPRB No. 2 des Pays de la Loire<br>Immeuble Deurbroucq- 5, allee de l'ile Gloriette<br>44093 Nantes Cedex 01<br>FRANCE
1124	Dr. Jacques Durant		Dr. Eric Cua<br>Prof. Pierre P. Dellamonica<br>Dr. Francine DeSalvador<br>Dr. Isabelle Perbost<br>Pascal Pugliese<br>Dr. Veronique Rahelinirina	Hopital de l Archet 1<br>Service d Infectiologie<br>151 Route de St. Antoine<br>de Ginestiere BP 3079<br>Nice Cedex 3, 06, 06202<br>FRANCE	CCPPRB No. 2 des Pays de la Loire<br>Immeuble Deurbroucq- 5, allee de l'ile Gloriette<br>44093 Nantes Cedex 01<br>FRANCE

090177e1801b151d\Approved\Approved On: 03-Oct-2007 20:09

090177e1801b151d\Approved\Approved On: 03-Oct-2007 20:09

Germany

Coordinating Investigators:
<None Entered>

Center	Principal Investigator	Co-Investigator(s)	Sub-Investigator(s)	Address(es)	Institutional Review Board or Ethics Committee Address(es)
1007 *	Prof. Thomas Harrer		Michael Baeuerle<br>Dr. Ellen Harrer<br>Dr. Matthias Schmitt-Haendle	Universitaetsklinik Erlangen-Nuernberg<br>Innere Medizin III<br>Krankenhausstr. 12<br>Erlangen, 91054<br>GERMANY	Ethik-Kommission der Aerztekammer Nordrhein<br>Tersteegenstrasse 9<br>Duesseldorf, 40474<br>GERMANY
1018	Dr. Lutwinus Weitner		Dr. Axel Adam<br>Dr. Thomas Buhk<br>Dr. Stefan Fenske<br>Dr. Holger J. Gellermann<br>Mr. Hauri Goey<br>Mrs. Susanne H. Heesch<br>Dr. Knud Carl Schewe<br>Prof.Dr. Hans-Juergen Stellbrink	Schwerpunktpraxis HIV<br>Brennerstr. 71<br>Hamburg, 20099<br>GERMANY	Ethik-Kommission der Aerztekammer Nordrhein<br>Tersteegenstrasse 9<br>Duesseldorf, 40474<br>GERMANY
1019	Dr. Lothar Schneider		Rasim Guler	Internist, HIV Schwerpunkt<br>Pickertstr. 2<br>Fuerth, 90762<br>GERMANY	Ethik-Kommission der Aerztekammer Nordrhein<br>Tersteegenstrasse 9<br>Duesseldorf, 40474<br>GERMANY
1024	Dr. Stefan Esser		Robert Jablonka<br>Simone Kohaus<br>Dr. Maija Koppermann<br>Dr. Birgit Ross	Klinik u. Poliklinik f. Dermatologie, Venerologie u. Allergologie, Universitaetsklinikum Essen<br>Hufelandstr. 55<br>Essen, 45122<br>GERMANY	Ethik-Kommission der Aerztekammer Nordrhein<br>Tersteegenstrasse 9<br>Duesseldorf, 40474<br>GERMANY
1025	Prof. Dr. Peter Kern		Dr. Beate Gruner<br>Dr. Georg Haerter<br>PD Dr. Stefan Reuter	Universitaetsklinik, Abteilung Innere Medizin III, Infektiologie und Klinische Immunologie<br>Robert-Koch-Strasse 8<br>Ulm, 89081<br>GERMANY	Ethik-Kommission der Aerztekammer Nordrhein<br>Tersteegenstrasse 9<br>Duesseldorf, 40474<br>GERMANY

* Did not randomize subjects

090177e1801b151d\Approved\Approved On: 03-Oct-2007 20:09

Center	Principal Investigator	Co-Investigator(s)	Sub-Investigator(s)	Address(es)	Institutional Review Board or Ethics Committee Address(es)
1041	Prof. Dr. Reinhold E. Schmidt		Dr. Hans Heiken<br>Dr. Jens Kittner<br>Margit Lubach-Ruitmann<br>Dirk Meyer-Olson<br>Ulrike Moebius<br>Prof. Dr. Matthias Stoll<br>Kai-Uwe Ulbricht	Medizinische Hochschule Hannover, Klinische Immunologie, Zentrum Innere Medizin<br>Carl-Neuberg-Strasse 1<br>Hannover, 30625<br>GERMANY	Ethik-Kommission der Aerztekammer Nordrhein<br>Tersteegenstrasse 9<br>Duesseldorf, 40474<br>GERMANY
1042	Prof. Johannes Richard Bogner<br>Prof. Dr. Frank-Detlef Goebel (Previous PI)		Dr. Jens Greiser<br>Nelleke Maaike Jakob<br>Mr. Boris Dominik Juelg<br>Dr. Helmut Liess<br>Carola Ludwig<br>Dr. Mirjam Osthoff<br>Matthias Sauter<br>Claudia Schaefer<br>Dr. Thomas Sternfeld<br>Dr. Bettina Supthut-Schroeder<br>Dr. Martina Maleticova Goetsch<br>Dr. Joerg Roling	Ludwig-Maximilians-Universitaet Medizinische Poliklinik - Klinikum Innenstadt<br>Pettenkoferstr. 8A<br>Muenchen, 80336<br>GERMANY	Ethik-Kommission der Aerztekammer Nordrhein<br>Tersteegenstrasse 9<br>Duesseldorf, 40474<br>GERMANY
1043	Dr. Jan van Lunzen<br>Prof.Dr. Hans-Juergen Stellbrink (Previous PI)		Dr. Olaf Degen<br>Sofie Elena Enderwitz<br>Claudia Schlesner<br>Ms. Bettina Guttowski<br>Dr. Alexander J. Zoufaly	Universitaetsklinikum Hamburg - Eppendorf<br>Innere Medizin, Medizinische Poliklinik Haus 057<br>Martinistrasse 52<br>Hamburg, 20246<br>GERMANY	Ethik-Kommission der Aerztekammer Nordrhein<br>Tersteegenstrasse 9<br>Duesseldorf, 40474<br>GERMANY
1044	Dr. Keikawus Arasteh		Jukka Marcus Hartikainen<br>Daniella Izbicki<br>Bastian Krondorfer<br>Michael Rittweger	EPIMED<br>c/o Vivantes-Auguste-Viktoria-Klinikum<br>Rubensstrasse 125<br>Berlin, 12157<br>GERMANY	Ethik-Kommission der Aerztekammer Nordrhein<br>Tersteegenstrasse 9<br>Duesseldorf, 40474<br>GERMANY

* Did not randomize subjects

Center	Principal Investigator	Co-Investigator(s)	Sub-Investigator(s)	Address(es)	Institutional Review Board or Ethics Committee Address(es)
1046	Prof. Dr.med. Schlomo Staszewski		Dr. Amina Carlebach<br>Dr. Annette Haberl<br>Guido Heidenreich-Urbach<br>Dr. med. Tessa Lennemann<br>Stephan Merten<br>Axel Mueller<br>Beate Nolte<br>Dr. Carsten Rottmann<br>Geetha Sarrach<br>Peter Schott<br>Mrs. Anja Von Nessen<br>Claudia Wengenroth<br>Kathleen Mantzsch<br>Gabriele Nisius<br>Dr. Na-Ri Oh	Klinikum der Johann Wolfgang Goethe-Universitaet, ZIM<br>Medizinische Klinik II<br>Infektionsambulanz/Schwerpunkt HIV<br>Theodor-Stern-Kai 7<br>Frankfurt, 60590<br>GERMANY	Ethik-Kommission der Aerztekammer Nordrhein<br>Tersteegenstrasse 9<br>Duesseldorf, 40474<br>GERMANY
1047	Prof. Dr.med. Andreas Plettenberg		Nicole Bade<br>Carola Floeter<br>Dr. Katrin Graefe<br>Dr. Stefan Hansen<br>Thore Lorenzen<br>Dr.med. Albrecht Stoehr<br>Stefan Unger<br>Regina Waldeck<br>Dr.med. Christian Hoffmann	ifi-Institut fuer interdiziplinaere Infektiologie & Immunologie GmbH<br>AK St. Georg, Haus K<br>Lohmuehlenstrasse 5<br>Hamburg, 20099<br>GERMANY	Ethik-Kommission der Aerztekammer Nordrhein<br>Tersteegenstrasse 9<br>Duesseldorf, 40474<br>GERMANY
1048	Dr. Andreas Trein MD		Eiko Schnaitmann<br>Denise Schoell<br>Ms. Aygul Turan	Arztpraxis<br>Schwabstrasse 57-59<br>Stuttgart, 70197<br>GERMANY	Ethik-Kommission der Aerztekammer Nordrhein<br>Tersteegenstrasse 9<br>Duesseldorf, 40474<br>GERMANY

090177e1801b151d\Approved\Approved On: 03-Oct-2007 20:09

Center	Principal Investigator	Co-Investigator(s)	Sub-Investigator(s)	Address(es)	Institutional Review Board or Ethics Committee Address(es)
1069	Prof. Dr. med. Gerd Faetkenheuer		Dr. Kai Breuer<br>PD Dr. med Oliver A. Cornely<br>Dr. Pia Hartmann<br>Dr. Gisela-Elisabeth Kremer<br>Dr. med. Tim Kuemmerle<br>Dr. Clara Lehmann<br>Eleonore Rund<br>Dr.med. Jan Rybniker<br>Dr. Christoph Wyen	Universitaetsklinik Koeln<br>Klinik I fuer Innere Medizin<br>Joseph-Stelzmann-Str 9<br>Koeln, 50924<br>GERMANY	Ethik-Kommission der<br>Aerztekammer Nordrhein<br>Tersteegenstrasse 9<br>Duesseldorf, 40474<br>GERMANY
1092	Prof. Dr. Juergen Rockstroh		Mrs. Anja Nixdorf<br>Dr. Carolynne Schwarze-Zander<br>Dr. Martin Vogel<br>Dr. Esther Voigt<br>Dr. Jan-Christian Wasmuth	Rheinische-Friedrich-Wilhelms-Universitaet Bonn,<br>Infektionsambulanz HIV<br>Sigmund Freud Str 25<br>Bonn, 53105<br>GERMANY	Ethik-Kommission der<br>Aerztekammer Nordrhein<br>Tersteegenstrasse 9<br>Duesseldorf, 40474<br>GERMANY
1112 *	Dr. Heribert Knechten		Dr. Beate Keldenich<br>Christian Hohn	Arztpraxis fuer Innere Medizin<br>Blondelstr 9<br>Aachen, 52062<br>GERMANY	Ethik-Kommission der<br>Aerztekammer Nordrhein<br>Tersteegenstrasse 9<br>Duesseldorf, 40474<br>GERMANY
1113	Prof. Dr. Norbert H. Brockmeyer		Tanja Blome<br>Claudia Nabring<br>Dr. Dirk Pabst<br>Dr. Anja Potthoff<br>Dr. Renate Schlottmann	Klinik fuer Dermatologie und Allergologie der Ruhr-Uni<br>St. Josef Hospital<br>Gudrunstrasse 56<br>Bochum, 44791<br>GERMANY	Ethik-Kommission der<br>Aerztekammer Nordrhein<br>Tersteegenstrasse 9<br>Duesseldorf, 40474<br>GERMANY
1144	Dr. Antonius Mutz		Dr. Wilhelmine Rolle	Staedtische Kliniken Natruper Holz<br>Infektionsambulanz HIV<br>Sedanstr. 115<br>Osnabrueck, 49090<br>GERMANY	Ethik-Kommission der<br>Aerztekammer Nordrhein<br>Tersteegenstrasse 9<br>Duesseldorf, 40474<br>GERMANY

* Did not randomize subjects

090177e1801b151d\Approved\Approved On: 03-Oct-2007 20:09

Center	Principal Investigator	Co-Investigator(s)	Sub-Investigator(s)	Address(es)	Institutional Review Board or Ethics Committee Address(es)
1146	Dr. med. Jorg-Andres Rump		Dr. Susanne Usadel	Arztpraxis<br>Merianstr. 5<br>Freiburg, 79098<br>GERMANY	Ethik-Kommission der<br>Aerztekammer Nordrhein<br>Tersteegenstrasse 9<br>Duesseldorf, 40474<br>GERMANY
1147	Dr. Dieter Schuster		Dr. Juergen Brust<br>Gundula Klag<br>Dr. Nicole Pittack	Schwerpunktpraxis<br>Q5, 14-22<br>Mannheim, 68161<br>GERMANY	Ethik-Kommission der<br>Aerztekammer Nordrhein<br>Tersteegenstrasse 9<br>Duesseldorf, 40474<br>GERMANY
1154	Dr. Mark Oette		Carola Blondin<br>Bjoern-Erik Ole Jensen<br>Dr. Arne Kroidl<br>Dr.med. Ralf Winzer<br>Stefanie Koch	Universitaetsklinik Duesseldorf,<br>Klinik fuer Gastroenterologie und Infektiologie<br>HIV- Ambulanz MX1 Ambulanz,<br>Geb. 1166<br>Moorenstrasse 5<br>Duesseldorf, 40225<br>GERMANY	Ethik-Kommission der<br>Aerztekammer Nordrhein<br>Tersteegenstrasse 9<br>Duesseldorf, 40474<br>GERMANY
1170	Dr. Markus Mueller		Dr. Bernhard Frietsch<br>Dr. Albrecht Ulmer	Gemeinschaftspraxis Dres. A. Ulmer, B. Frietsch, M. Mueller<br>Schwabstrasse 26<br>Stuttgart, 70197<br>GERMANY	Ethik-Kommission der<br>Aerztekammer Nordrhein<br>Tersteegenstrasse 9<br>Duesseldorf, 40474<br>GERMANY

* Did not randomize subjects

090177e1801b151d\Approved\Approved On: 03-Oct-2007 20:09

Italy

Coordinating Investigators:
<None Entered>

Center	Principal Investigator	Co-Investigator(s)	Sub-Investigator(s)	Address(es)	Institutional Review Board or Ethics Committee Address(es)
1016	Dr. Francesco Mazzotta		Dr. Massimo Di Pietro<br>Dr. Sergio Lo Caputo<br>Dr. Piera Pierotti	Ospedale Santa Maria Annunziata<br>Unità Operativa di Malattie Infettive<br>Via dell'Antella 58<br>Antella, FIRENZE 50011<br>ITALY	Comitato Etico per la Sperimentazione Clinica del Medicinali<br>C/O SS Convenzioni per prestazioni Sanitarie<br>Azienda Sanitaria di Firenze (Azienda USL 10)<br>Via Gabriele D'Annunzio 29<br>Firenze, 50135<br>ITALY
1017	Dr. Pietro Caramello		Dr. Sinibaldo Carosella<br>Dr. Mariana Farenga<br>Dr. Gian Carlo Orofino<br>Dr. Silvia Quaglia	Ospedale Amedeo Savoia<br>I Div. di Malattie Infettive<br>Corso Svizzera 164<br>Torino, 10149<br>ITALY	Commissione Regionale per le Sperimentazioni Cliniche<br>Assessorato alla Sanità<br>Regione Piemonte<br>Viale Regina Margherita, 153/bis<br>TORINO, 10100<br>ITALY
1026	Dr. Antonio Di Biagio<br>Prof. Dante Bassetti (Previous PI)		Dr. Matteo Bassetti<br>Dr. Francesca Bisio<br>Dr. Chiara Dentone<br>Dr. Raffaella Rosso	Ospedale San Martino<br>Clinica di Malattie Infettive<br>Largo R. Benzi 10<br>Genova, 16132<br>ITALY	Comitato Etico dell' Azienda Ospedaliera San Martino<br>Largo Rosanna Benzi 10<br>Genova, 16132<br>ITALY
1049	Prof. Giampiero Carosi		Dr. Francesca Moretti<br>Dr. Eugenia Quiros Roldan<br>Dr. Valeria Tirelli<br>Ms. Maria Cristina Uccelli	Spedali Civili di Brescia<br>Dipatimento Malattie Infettive<br>Piazza Spedali Civili, 1<br>Brescia, 25123<br>ITALY	Comitato Etico Azienda Spedali Civili di Brescia<br>P. le Spedali Civili, L<br>Brescia, 25123<br>ITALY

090177e1801b151d\Approved\Approved On: 03-Oct-2007 20:09

Center	Principal Investigator	Co-Investigator(s)	Sub-Investigator(s)	Address(es)	Institutional Review Board or Ethics Committee Address(es)
1050	Prof. Roberto Esposito		Dr. Barbara Beghetto<br>Dr. Vanni Borghi<br>Dr. Giovanni Guaraldi<br>Dr. Nicola Mongiardo<br>Dr. Cristina Mussini<br>Dr. Giulia Nardini	Ospedale di Modena<br>Clinica di Malattie Infettive e Tropicali<br>Via del Pozzo, 71<br>Modena, 41100<br>ITALY	Comitato Etico Provinciale di Modena<br>Universita degli Studi di Modena e Reggio Emilia<br>Via del Pozzo 71<br>Modena, 41100<br>ITALY
1051	Prof. Adriano Lazzarin		Liviana D. Della Torre<br>Dr. Silvia Nozza<br>Dr.ssa Vega E. Rusconi<br>Dr. Giuseppe Tambussi	Centro Ricerca e cura Ospedale San Raffaele<br>Divisione di Malattie Infettive<br>Via Stamira d'Ancona, 20<br>Milano, 20100<br>ITALY	Comitato Etico Fondazione Centro San Raffaele del Monte Tabor<br>Istituto Scientifico Ospedale San Raffaele<br>Via Olgettina, 60<br>Milano, 20132<br>ITALY
1070 *	Prof Fernando Aiuti		Dr. Valentina Conti<br>Dr. Wladimiro De Santis<br>Dr. Alessandra Fantauzzi<br>Dr. Caterina Fimiani<br>Dr. Helene Martini<br>Dr. Ivano Mezzaroma	Università La Sapienza - Policlinico Umberto I<br>Dipartmento di Medicina Clinica<br>Roma<br>ITALY	Comitato Etico dell' Azienda Ospedaliera Univeritaria Policlinico Umberto I<br>Viale del Policlinico 155, ROMA 00185<br>ITALY
1075	Prof. Francesco Leoncini		Dr. Beatrice Borchi<br>Dr. Antonio Carocci<br>Dr. Riccardo Giuntini<br>Dr. Gaetana Sterrantino<br>Dr. Sergio M. Marchi	Azienda Ospedaliera Careggi<br>U.O. di Malattie Infettive<br>Viale G. Pieraccini 17<br>Firenze, 50193<br>ITALY	Comitato Etico<br>Azienda Ospedaliera Careggi<br>Viale Morgagni 85<br>Firenze, 50139<br>ITALY
1078 *	Prof. Vincenzo Vullo		Dr. Martina Carnevalini<br>Dr. Claudia D'Agostino<br>Dr. Miriam Lichtner<br>Dr. Anna Paola Massetti<br>Prof. Claudio Mastroianni<br>Ginevra Azzurra Miccoli	Università di Roma "La Sapienza"<br>Dipartmento di Malattie Infettive<br>Viale del Policlinico, 155<br>Roma, 00185<br>ITALY	Comitato Etico<br>dell'Azienda Policlinico Umberto I<br>Viale del Polilinico, 155<br>Roma, 00161<br>ITALY

* Did not randomize subjects

090177e1801b151d\Approved\Approved On: 03-Oct-2007 20:09

Center	Principal Investigator	Co-Investigator(s)	Sub-Investigator(s)	Address(es)	Institutional Review Board or Ethics Committee Address(es)
1149	Dr. Francesco Montella		Dr. Fiorella Di Sora<br>Dr. Agapito Tarasi	Ospedale San Giovanni<br>Unità di Immunologia<br>Via San Giovanni in Laterano, 155<br>Roma, RM 00184<br>ITALY	Comitato Etico dell' Ospedale San Giovanni<br>Via dell' Amba Aradan 8<br>Rome, 00184<br>ITALY
1178 *	Prof. Enzo Raise		Dr. Francesca Ebo<br>Dr. Franklyn Eseme	Ospedale SS. Giovanni e Paolo<br>Div di Malattie Infettive<br>Campo SS Giovanni e Paolo<br>Venezia, 30170<br>ITALY	COMITATO ETICO VALUTAZ. DELLE SPERIMENTAZIONI CLINICHE DEI MEDICINALI DEL POL. UNIV. DI CAGLIARI<br>Via S. Giorgio,12<br>Cagliari, 09100<br>ITALY
1179	Prof. Paolo Emilio Manconi		Dr. Nicoletta Corso<br>Dr. Alessandro Masala<br>Dr. Francesco Ortu<br>Dr. Paola Piano	Poloclinico Universiatario<br>Scienze Mediche "M. Aresu"<br>SS 554 per Bivio Sestu<br>Monserrato-Cagliari, CA 09042<br>ITALY	COMITATO ETICO VALUTAZ. DELLE SPERIMENTAZIONI CLINICHE DEI MEDICINALI DEL POL. UNIV. DI CAGLIARI<br>Via S. Giorgio,12<br>Cagliari, 09100<br>ITALY

090177e1801b151d\Approved\Approved On: 03-Oct-2007 20:09

* Did not randomize subjects

090177e1801b151d\Approved\Approved On: 03-Oct-2007 20:09

Netherlands

Coordinating Investigators:
<None Entered>

Center	Principal Investigator	Co-Investigator(s)	Sub-Investigator(s)	Address(es)	Institutional Review Board or Ethics Committee Address(es)
1056 *	Dr. Marchina Elisabeth Van Der Ende		Maria Deenenkamp<br>Dr. Machteld Van der Feltz	Erasmus MC<br>Locatie Centrum<br>Dr Molewaterplein 40<br>Rotterdam, 3015 GD<br>NETHERLANDS	METC UMCU<br>Heidelberglaan 100<br>3584 CX<br>Utrecht<br>NETHERLANDS
1057 *	Dr. Clemens Richter		Nienke Langebeek<br>Petra Van Bentum<br>Dr. Joop Van den Berg<br>Dr Juultje Van Der Berg	Ziekenhuis Rijnstate<br>Wagnerlaan 55<br>Arnhem, 6815 AD<br>NETHERLANDS	METC UMCU<br>Heidelberglaan 100<br>3584 CX<br>Utrecht<br>NETHERLANDS
1080	Prof. Ilja Mohandas Hoepelman		Dr. Pauline M. de Puy-Ellerbroek<br>Bert Fledderus<br>Dr. Michael A. Gaytant<br>Joke Patist<br>Dr. Aukje Rijkeboer<br>Dr. James Cohen Stuart<br>Steven F.L. Van Lelyveld	University Medical Center Utrecht<br>Dept of Internal Medicine &<br>Infection Diseases, F02 - 126<br>Heidelberglaan 100<br>Utrecht, 3584 CX<br>NETHERLANDS	METC UMCU<br>Heidelberglaan 100<br>3584 CX<br>Utrecht<br>NETHERLANDS
1083 *	Dr. Rosalinde Maria Perenboom		Dr. Frans Claessen<br>Dr. Sven A. Danner<br>Linda Hegeman<br>Dr. Michiel Van Agtmael<br>Dr. Marit Van Vonderen	VU Medical Centre<br>Dept of Internal Medicine 4A-38<br>De Boelelaan 1117<br>Amsterdam, 1081 HV<br>NETHERLANDS	METC UMCU<br>Heidelberglaan 100<br>3584 CX<br>Utrecht<br>NETHERLANDS
1084 *	Dr. Christiaan Ten Napel		Dr. Gerritt Jan Kootstra<br>Dr. Helene Wiggers<br>Margo van de Burg	Medisch Spectrum Twente<br>Internal Medicine, Infection Diseases 53D<br>Ariensplein 1<br>Enschede, 7500 KA<br>NETHERLANDS	METC UMCU<br>Heidelberglaan 100<br>3584 CX<br>Utrecht<br>NETHERLANDS

* Did not randomize subjects

Center	Principal Investigator	Co-Investigator(s)	Sub-Investigator(s)	Address(es)	Institutional Review Board or Ethics Committee Address(es)
1103	Dr. J. M. Prins		Hans-Erik Nobel<br>Dr. Gitte Van Twillert<br>Michele Van Vught	Academisch Centrum<br>Universiteit van Amsterdam<br>Meibergdreef 9<br>Amsterdam, 1105 AZ<br>NETHERLANDS	METC UMCU<br>Heidelberglaan 100<br>3584 CX<br>Utrecht<br>NETHERLANDS

090177e1801b151d\Approved\Approved On: 03-Oct-2007 20:09

* Did not randomize subjects

090177e1801b151d\Approved\Approved On: 03-Oct-2007 20:09

Poland

Coordinating Investigators:
<None Entered>

Center	Principal Investigator	Co-Investigator(s)	Sub-Investigator(s)	Address(es)	Institutional Review Board or Ethics Committee Address(es)
1052	Dr. Marek Beniowski		Dr. Jakub Kwiatkowski<br>Dr. Elzbieta Mularska<br>Dr. Adam Witor	Szpital Specjalistyczny Centrum Diagnostyki i Terapii AIDS<br>ul. Zjednoczenia 10<br>Chorzow, 41-500<br>POLAND	Komisja Bioetyczna przy Centrum Medycznym Ksztalcenia Podyplomowego<br>ul. Marymoncka 99<br>01 - 813<br>Warszawa<br>POLAND
1053 *	Prof. Anna Boron-Kaczmarska		Dr. Dorota Bander<br>Dr. Magdalena Leszczyszyn-Pynka<br>Dr. Anita Wnuk	SP Wojewodzki Szpital Zespolony im. M. Sklodowskiej - Curie<br>Katedra i Klinika Chorob Zakaznych PAM<br>ul. Arkonska 4<br>Szczecin, 71-455<br>POLAND	Komisja Bioetyczna przy Centrum Medycznym Ksztalcenia Podyplomowego<br>ul. Marymoncka 99<br>01 - 813<br>Warszawa<br>POLAND
1054 *	Prof. Waldemar Halota		Dr. Edyta Grabczewska<br>Dr. Anita Olczak<br>Dr. Malgorzata Pawlowska	Wojewodzki Szpital Obserwacyjno - Zakazny<br>Klinika Chorob Zakaznych<br>ul. Floriana 12<br>Bydgoszcz, 85-030<br>POLAND	Komisja Bioetyczna przy Centrum Medycznym Ksztalcenia Podyplomowego<br>ul. Marymoncka 99<br>01 - 813<br>Warszawa<br>POLAND
1055	Dr. Andrzej Horban		Dr. Elzbieta Bakowska<br>Dr. Anna Ignatowska<br>Dr. Grzegorz Karczewski<br>Dr. Piotr Pulik	Wojewodzki Szpital Zakazny Centrum Diagnostyki i Terapii AIDS<br>ul. Wolska 37<br>Warszawa, 01-201<br>POLAND	Komisja Bioetyczna przy Centrum Medycznym Ksztalcenia Podyplomowego<br>ul. Marymoncka 99<br>01 - 813<br>Warszawa<br>POLAND

* Did not randomize subjects

Center	Principal Investigator	Co-Investigator(s)	Sub-Investigator(s)	Address(es)	Institutional Review Board or Ethics Committee Address(es)
1094 *	Prof. Tomasz Mach		Dr. Monika Bociaga-Jasik<br>Dr. Pawel Skwara	Szpital Uniwersytecki, Klinika Chorob Zakaznych<br>ul. Sniadeckich 5<br>Krakow, 31-531<br>POLAND	Komisja Bioetyczna przy Centrum Medycznym Ksztalcenia Podyplomowego<br>ul. Marymoncka 99<br>01 - 813<br>Warszawa<br>POLAND
1116 *	Dr. Tomasz Smiatacz		Dr. Marta Gesing<br>Dr. Maria Jankowska<br>Dr. Malgorzata Lemanska<br>Dr. Hanna Trocha	Wojewodzki Szpital Zakazny, Klinika Chorob Zakaznych AM<br>ul. Smoluchowskiego 18<br>Gdansk, 80-214<br>POLAND	Komisja Bioetyczna przy Centrum Medycznym Ksztalcenia Podyplomowego<br>ul. Marymoncka 99<br>01 - 813<br>Warszawa<br>POLAND
1117	Prof. Robert Flisiak		Prof. Danuta Prokopowicz<br>Dr. Ewa Siwak<br>Dr. Alicja Wiercinska-Drapalo	Klinika Obserwacyjno-Zakazna AM<br>Wojewodzki Szpital Specjalistyczny im. Dluskiego<br>ul. Zurawia 14<br>Bialystok, 15-540<br>POLAND	Komisja Bioetyczna przy Centrum Medycznym Ksztalcenia Podyplomowego<br>ul. Marymoncka 99<br>01 - 813<br>Warszawa<br>POLAND

* Did not randomize subjects

090177e1801b151d\Approved\Approved On: 03-Oct-2007 20:09

Spain

Coordinating Investigators:
Dr. Antonio Alcolea Pilz
Jordi Puig Pla
Dr. Jesus Fortún (Previous Coordinating Investigator)

Center	Principal Investigator	Co-Investigator(s)	Sub-Investigator(s)	Address(es)	Institutional Review Board or Ethics Committee Address(es)
1002	Jose Josep Gatell Artigas		Dr. Jose Luis Blanco<br>Dr. Agatha Leon	Hospital Clinic I Provincial<br>Enfermedades Infecciones<br>C/. Villarroel 170<br>Barcelona, Barcelona 08036<br>SPAIN	Comite Etico de Investigacion Clinica<br>Hospital Clinic i Provincial<br>C/Villarroel, 170<br>Barcelona, 08036<br>SPAIN<br><br>Comite Etico de Investigacion Clinica<br>Hospital Germans Trias I Pujol<br>Ctra. De Canyet s/n<br>Badalona, Barcelona 08916<br>SPAIN
1003	Dr. Juan Gonzalez Lahoz		Dr. Luz Martin Carbonero<br>Pilar MaGarcia<br>Dr. Francisco Blanco Quintana<br>Dr. Antonio Alcolea Pilz	Hospital Carlos III<br>Servicio de Enfermedades Infecciosas<br>C/ Sinesio Delgado, 10-12<br>Madrid, Madrid 28029<br>SPAIN	Comite Etico de Investigacion Clinica<br>Hospital Carlos III<br>C/Sinesio Delgado 10<br>Madrid, 28029<br>SPAIN<br><br>Comite Etico de Investigacion Clinica<br>Hospital Germans Trias I Pujol<br>Ctra. De Canyet s/n<br>Badalona, Barcelona 08916<br>SPAIN
1004	Dr. Bonaventura Clotet Sala		Dr. Anna Bonjoch<br>Isabel Bravo Onraita<br>Juan Carlos Martinez<br>Jose Miranda<br>Dr. Eugenia Negredo Puigmal<br>Jordi Puig Pla	Hospital Universitario Germans Trias i Pujol<br>Infectious Diseases<br>Ctra. Del Canyet, s/n<br>Badalona, Barcelona 08916<br>SPAIN	Comite Etico de Investigacion Clinica<br>Hospital Germans Trias I Pujol<br>Ctra. De Canyet s/n<br>Badalona, Barcelona 08916<br>SPAIN

090177e1801b151d\Approved\Approved On: 03-Oct-2007 20:09

090177e1801b151d\Approved\Approved On: 03-Oct-2007 20:09

Center	Principal Investigator	Co-Investigator(s)	Sub-Investigator(s)	Address(es)	Institutional Review Board or Ethics Committee Address(es)
1012	Dr. Jose Iribarren Loyarte		Julia Arrizabalaga<br>Dr. Francisco Rodriguez Arrondo<br>Dr. Xabier Camino<br>Dr. Miguel Angel Von Bichmann de Miguel	Hospital de Donostia<br>Enfermedades infecciosas<br>Pº Dr. Beguiristain, 109<br>San Sebastian , 20014<br>SPAIN	Comite Etico de Investigacion Clinica<br>Hospital de Donosita<br>Paseo Dr. Beguiristain, 109<br>San Sebastian, 20014<br>SPAIN<br><br>Comite Etico de Investigacion Clinica<br>Hospital Germans Trias I Pujol<br>Ctra. De Canyet s/n<br>Badalona, Barcelona 08916<br>SPAIN
1013	Dr. Santiago Moreno		Dr. Antonio Antela<br>Dr. Jose Luis Casado<br>Dr. Fernando Dronda<br>Ana Moreno<br>Rosa Perez<br>Dr. M. Jesus Perez-Elias	Hospital Ramon y Cajal<br>Servicio de Enfermedades Infecciosas<br>Carretera Colmenar Viejo, Km. 9. 100<br>Madrid, 28034<br>SPAIN	Comite Etico de Investigacion Clinica<br>Hospital Germans Trias I Pujol<br>Ctra. De Canyet s/n<br>Badalona, Barcelona 08916<br>SPAIN<br><br>Comite Etico de Investigacion Clinica<br>Hospital Ramon y Cajal<br>Ctra.Colmenar Viejo Km 9,1<br>Madrid, 28034<br>SPAIN

* Did not randomize subjects

Center	Principal Investigator	Co-Investigator(s)	Sub-Investigator(s)	Address(es)	Institutional Review Board or Ethics Committee Address(es)
1014	Dr. Cristina Sarria		Dr. M. Carmen Martinez Garcia<br>Dr. Jesus Sanz Sanz	Hospital de La Princesa<br>Servicio Enfermedades Infecciosas. VIH.<br>C/ Diego de Leon, 62<br>Madrid, Madrid 28006<br>SPAIN	Comite Etico de Investigacion Clinica<br>Hospital Universitario de La Princesa<br>C/ Diego de Leon 62<br>MADRID, MADRID 28006<br>SPAIN<br><br>Comite Etico de Investigacion Clinica<br>Hospital Germans Trias I Pujol<br>Ctra. De Canyet s/n<br>Badalona, Barcelona 08916<br>SPAIN
1015	Dr. Federico Pulido Ortega		Concepcion Cepeda<br>Rafael Hervas	Hospital 12 de Octubre<br>Unidad Infeccion VIH<br>Medicina Interna<br>Ctra de andalucia Km 5.4<br>Madrid, Madrid 28041<br>SPAIN	Comite Etico de Investigacion Clinica<br>Hospital Germans Trias I Pujol<br>Ctra. De Canyet s/n<br>Badalona, Barcelona 08916<br>SPAIN<br><br>Comite Etico de Investigacion Clinica<br>Hospital Universitario 12 de Octubre<br>Ctra. Andalucia Km 5,4<br>MADRID, 28041<br>SPAIN
1059	Dr. Juan Julian Gonzalez Garcia		Dr. Jose Ramon Arribas<br>Dr. Alicia Lorenzo Hernandez<br>Dr. Maria Luisa Montes<br>Dr. Rosa Maria Munoz de Benito<br>Dr. Jose M. Pena	Hospital Univ. La Paz<br>Unidad VIH<br>Pº de la Castellana, 261<br>Madrid, Madrid 28046<br>SPAIN	Comite Etico de Investigacion Clinica<br>Hospital La Paz<br>Paseo de la Castellana 261<br>Madrid, 28046<br>SPAIN<br><br>Comite Etico de Investigacion Clinica<br>Hospital Germans Trias I Pujol<br>Ctra. De Canyet s/n<br>Badalona, Barcelona 08916<br>SPAIN

090177e1801b151d\Approved\Approved On: 03-Oct-2007 20:09

Center	Principal Investigator	Co-Investigator(s)	Sub-Investigator(s)	Address(es)	Institutional Review Board or Ethics Committee Address(es)
1060 *	Dr. Pere Domingo Pedrol		Dr. Josep Cadafalch Arpa<br>Dr. M. Antonia Sambeat Domenech<br>Dr. Montserrat Fuster<br>Dr. Gracia Mateo Garcia<br>Dr. Mar Gutierrez Macia	Hospital De La Santa Creu i Sant Pau<br>Servicio de Enfermedades Infecciosas. VIH<br>Avda. Sant Antoni Maria Claret 167<br>Barcelona, Barcelona 08025<br>SPAIN	Comite Etico de Invesitacion Clinica<br>Hospital de la Santa Creu i Sant Pau<br>Avda. Antonio Maria Claret, 167<br>Barcelona, 08025<br>SPAIN<br><br>Comite Etico de Investigacion Clinica<br>Hospital Germans Trias I Pujol<br>Ctra. De Canyet s/n<br>Badalona, Barcelona 08916<br>SPAIN
1061	Dr. Felix Gutierrez		Maria del Mar Masia<br>Dr. Enrique Bernal Morell<br>Dr. Sergio Padilla Urrea	Hospital General de Elche<br>Unidad de Infecciosas<br>C/ Partida Huertos y Molinos s/n<br>Elche, Alicante 03202<br>SPAIN	Comite Etico de Investigacion Clinica<br>Hospital Germans Trias I Pujol<br>Ctra. De Canyet s/n<br>Badalona, Barcelona 08916<br>SPAIN<br><br>Hospital General Universitario de Elche<br>Comite Etico de Investigacion Clinica<br>Cami del a Almazara No.11<br>Elche, Alicante 03202<br>SPAIN

* Did not randomize subjects

090177e1801b151d\Approved\Approved On: 03-Oct-2007 20:09

Center	Principal Investigator	Co-Investigator(s)	Sub-Investigator(s)	Address(es)	Institutional Review Board or Ethics Committee Address(es)
1062	Dr. Pompeyo Viciana Fernandez		Dr. Luis Fernando Lopez Crotes<br>Rosario Mata	Hospital Universitario Virgen del Rocio<br>Unidad Clinica de Enfermedades Infecciosas<br>Avda. Manuel Siurot, s/n<br>Sevilla, Sevilla 41013<br>SPAIN	Comite Autonomico de Ensayos Clinicos de Andalucia<br>Consejeria de Salud<br>Avda. de la Innovacion, s/n - Edificio Arena 1, Sevilla 41020<br>SPAIN<br><br>Comite Etico de Investigacion Clinica<br>Hospital Germans Trias I Pujol<br>Ctra. De Canyet s/n<br>Badalona, Barcelona 08916<br>SPAIN<br><br>HOSPITAL VIRGEN DEL ROCIO<br>Comite Etico de Investigacion Clinica<br>Avda. Manuel Siurot, S/N<br>SEVILLA, SEVILLA 41013<br>SPAIN
1096	Dr. Antonio Rivero		Dr. Angela Camacho Espeso<br>Dr. Julian de La Torre Cisneros<br>Dr. Milagros Garcia Lazaro<br>Dr. Rafael Jurado Jimenez<br>Dr. Jose M. Kindelan<br>Dr. M. Carmen Montero Ponferrada	Hospital Reina Sofia<br>Servicio de Enfermedades Infecciosas<br>Avda. Menendez Pidal, s/n<br>Cordoba, Cordoba 14004<br>SPAIN	Comite Autonomico de Ensayos Clinicos de Andalucia<br>Consejeria de Salud<br>Avda. de la Innovacion, s/n - Edificio Arena 1, Sevilla 41020<br>SPAIN<br><br>Comite Etico de Investigacion Clinica<br>Hospital Germans Trias I Pujol<br>Ctra. De Canyet s/n<br>Badalona, Barcelona 08916<br>SPAIN<br><br>Hospital Reina Sofia<br>Comite Etico de Investigacion Clinica<br>Avda. Menendez Pidal, s/n<br>Cordoba, Cordoba 14004<br>SPAIN

* Did not randomize subjects

090177e1801b151d\Approved\Approved On: 03-Oct-2007 20:09

Center	Principal Investigator	Co-Investigator(s)	Sub-Investigator(s)	Address(es)	Institutional Review Board or Ethics Committee Address(es)
1104	Dr. Fernando Lozano de Leon		Dr. Eva Leon<br>Dr. Juan Antonio Pineda<br>Dr. Gloria Sebastian	Hospital Ntra. Sra. de Valme<br>Enfermedades Infecciosas<br>Autovia de Cadiz, s/n<br>Planta 7<br>Sevilla, Sevilla 41014<br>SPAIN	Comite Autonomico de Ensayos Clinicos de Andalucia<br>Consejeria de Salud<br>Avda. de la Innovacion, s/n - Edificio Arena 1, Sevilla 41020<br>SPAIN<br><br>Comite Etico de Investigacion Clinica<br>Hospital Germans Trias I Pujol<br>Ctra. De Canyet s/n<br>Badalona, Barcelona 08916<br>SPAIN<br><br>Comité Ético de Investigación Clínica<br>Hospital Universitario Nuestra Senora de Valme<br>Ctra. Cádiz s/n<br>Sevilla, 41014<br>SPAIN
1105	Dr. Rafael Rubio Garcia		Dr. Victor Julian Moreno	Hospital 12 de Octubre<br>Internal Medicine<br>Ctra. de Andalucia Km. 5400<br>Madrid, Madrid 28041<br>SPAIN	Comite Etico de Investigacion Clinica<br>Hospital Germans Trias I Pujol<br>Ctra. De Canyet s/n<br>Badalona, Barcelona 08916<br>SPAIN<br><br>Comite Etico de Investigacion Clinica<br>Hospital Universitario 12 de Octubre<br>Ctra. Andalucia Km 5,4<br>MADRID, 28041<br>SPAIN

090177e1801b151d\Approved\Approved On: 03-Oct-2007 20:09

Sweden

Coordinating Investigators:
<None Entered>

Center	Principal Investigator	Co-Investigator(s)	Sub-Investigator(s)	Address(es)	Institutional Review Board or Ethics Committee Address(es)
1063 *	Prof. Magnus Gisslen		Lars-Magnus Andersson<br>Dr. Lars Hagberg<br>Lissie Johansson	Sahlgrenska<br>Universitetssjukhuset<br>Infektionskliniken<br>Göteborg, 41685<br>SWEDEN	The Regional Ethical Research<br>Committee in Stockholm<br>Box 289<br>Stockholm, 171 77<br>SWEDEN
1097 *	Leo Flamholc		Ulla Akerholm<br>Dr. Per Bjorkman<br>Dr. Ewa Wallmark	Universitetssjukhuset MAS<br>Infektionskliniken<br>Malmö , 214 01<br>SWEDEN	The Regional Ethical Research<br>Committee in Stockholm<br>Box 289<br>Stockholm, 171 77<br>SWEDEN
1115	Dr. Anders Karlsson		Dr. Magnus Hedenstierna<br>Inger Petz	Dr Anders Karlsson<br>Sodersjukhuset<br>Venhalsan, Stockholm 118 83<br>SWEDEN	The Regional Ethical Research<br>Committee in Stockholm<br>Box 289<br>Stockholm, 171 77<br>SWEDEN

090177e1801b151d\Approved\Approved On: 03-Oct-2007 20:09

* Did not randomize subjects

Switzerland

Coordinating Investigators:
<None Entered>

Center	Principal Investigator	Co-Investigator(s)	Sub-Investigator(s)	Address(es)	Institutional Review Board or Ethics Committee Address(es)
1010	Prof. Dr. Bernard Hirschel		Dr. Sophie Claire Ghislaine Crespo	Hôpitaux Universitaires de Genève - HUG<br>Department de medicine interne, maladies infectieuses<br>24, rue Micheli-du-Crest<br>1211 Genève, 14<br>SWITZERLAND	Commission centrale d'ethique de la recherche<br>sur l etre humaine des Hopitaux Universitaires de Geneve (HUG)<br>President: Prof: Henri Bounameaux<br>Div. d'angiologie et hemostasc<br>Hopital Cantonal Universitaires<br>Geneve 14<br>SWITZERLAND
1011	Prof. Dr. Milos Opravil		Dr. Milo Emil Huber	Universitätsspital Zürich<br>Abteilung Infektionskrankheiten und Spitalhygiene<br>Rämistrasse 100<br>Zürich, 8091<br>SWITZERLAND	SPUK fur innere Medizin<br>Prof. Dr med. Jurg Muller<br>USZ, Dep. Innere Med.<br>Ramistrasse 100<br>Zurich, 8091<br>SWITZERLAND
1106	Dr. Matthias Cavassini		Dr. Oriol Manuel Altes<br>Dr. Caroline Chapuis-Taillard<br>Claudia Franc<br>Dr. Patricia Halfon<br>Dr. Mona Khonkarly<br>Dr. Owen Robinson<br>Philip Tarr	CHUV<br>Departément de Médicine Interne<br>Rue du Bugnon 46<br>Lausanne, 1011<br>SWITZERLAND	Commission d'éthique de la recherche clinique<br>de la Faculte de Biologie et de Medecine<br>Prof. Michel Burnier (president centrale)<br>Faculté de Médecine Secretariat central<br>Rue du Bugnon 21<br>Lausanne, 1005<br>SWITZERLAND
1107 *	Dr. Enos Bernasconi		Dr. Lorenzo Magenta	Ospedale Regionale di Lugano Sede<br>Ospedale Civico, Ambulatorio Malattie Infecttive<br>Via Tesserete 46<br>Lugano, 6900<br>SWITZERLAND	Comitato Etico Cantonale<br>c/o Sezione sanitaria<br>Via Orico 5<br>Bellinzona, TI CH-6500<br>SWITZERLAND

* Did not randomize subjects

090177e1801b151d\Approved\Approved On: 03-Oct-2007 20:09

Center	Principal Investigator	Co-Investigator(s)	Sub-Investigator(s)	Address(es)	Institutional Review Board or Ethics Committee Address(es)
1114	Prof. Dr. Manuel Battegay<br>Dr. Henning Drechsler (Previous PI)		Dr. Luigia Elzi<br>Dr. Jan Sven Fehr<br>Dr. Erik Mossdorf<br>Verena Werder<br>Dr. Jan Sven Fehr	Kantonsspital Basel,<br>Medizinische Poliklinik<br>Petersgraben 4<br>Basel, 4031<br>SWITZERLAND	Ethikkommission beider Basel<br>EKBB<br>Prof. Dr Hans Kummer<br>Hebelstrasse 53<br>Basel<br>SWITZERLAND
1121	Dr. Pietro Vernazza		Dr. Serge Hediger<br>Dr. Arnob Roy<br>Dr. Patrick Schmid<br>Helen Weyermann	Kantonsspital St. Gallen<br>Infektologie - Innere Medizin<br>Klinik A<br>Rorschacherstrasse 95<br>St. Gallen, 9007<br>SWITZERLAND	Kantonale Ethikkommission des Kantons St. Gallen<br>Dr. G. Kreienbuhl<br>Rorschacherstrasse 95,<br>Kantonsspital<br>Haus 25/409, St Gallen 9007<br>SWITZERLAND

090177e1801b151d\Approved\Approved On: 03-Oct-2007 20:09

* Did not randomize subjects

United Kingdom

Coordinating Investigators:
<None Entered>

Center	Principal Investigator	Co-Investigator(s)	Sub-Investigator(s)	Address(es)	Institutional Review Board or Ethics Committee Address(es)
1066	Dr. Clifford L. Leen		Dr. Janet M. Andrews<br>Laura Ellis<br>Dr. Claire McGoldrick<br>Ms. Sheila M. Morris<br>Dr. Hazel Heather Rae<br>Michael MacKenzie	Western General Infirmary<br>Haematology<br>Crewe Road<br>EDINBURGH, EH4 2XU<br>UNITED KINGDOM	Lothian Research Ethics Committee<br>Deaconess House<br>148 Pleasance<br>Edinburgh, EH8 9RS<br>UNITED KINGDOM<br><br>South East Multi Centre Research Ethics Committee<br>Kent & Medway Health Authority<br>Preston Hall<br>Aylesford<br>Maidtone, Kent ME20 7NJ<br>UNITED KINGDOM
1067 *	Dr. Martin Fisher		Dr. Kazeem Oladipo Aderogba<br>Dr. Angela C. Bailey<br>Katharine Bond<br>Dr. Duncan R. Churchill<br>Ms. Lisa A. Heald<br>Kristjan Helgason<br>Tara E. Maher<br>Dr. Alison J. Mears<br>Dr. Kate Nambiar<br>Dr. Usharani Natarajan<br>Dr. David S. Pao<br>Dr. Nicola L. Perry<br>Samantha K. Pushpakaran-Vimala<br>Dr. Iain C. Reeves<br>Jennifer Whetham	Brighton & Sussex University Hospitals NHS Trust<br>HIV/GUM Research Dept<br>The Elton John Centre<br>Sussex House, 1 Abbey Road<br>BRIGHTON, BN2 1ES<br>UNITED KINGDOM	Brighton & Hove Research Ethics Committee<br>Brighton & Hove Primary Care Trust<br>Sixth Floor, Vantage Point<br>New England Road<br>Brighton, BN1 4GW<br>UNITED KINGDOM<br><br>South East Multi Centre Research Ethics Committee<br>Kent & Medway Health Authority<br>Preston Hall<br>Aylesford<br>Maidtone, Kent ME20 7NJ<br>UNITED KINGDOM

090177e1801b151d\Approved\Approved On: 03-Oct-2007 20:09

Center	Principal Investigator	Co-Investigator(s)	Sub-Investigator(s)	Address(es)	Institutional Review Board or Ethics Committee Address(es)
1068 *	Dr. David J. White		Dr. Peter E. Bruck<br>Jayne Alison Groves	Birmingham Heartlands Hospital<br>Infectious Diseases / G.U. Medicine<br>51 Bordesley Green East<br>BIRMINGHAM, B9 5SS<br>UNITED KINGDOM	East Birmingham Local Research Ethics Committee<br>Warwick House<br>Birmingham Heartlands Hospital<br>Bordesley Green East<br>Birmingham, B9 5SS<br>UNITED KINGDOM<br><br>South East Multi Centre Research Ethics Committee<br>Kent & Medway Health Authority<br>Preston Hall<br>Aylesford<br>Maidtone, Kent ME20 7NJ<br>UNITED KINGDOM
1081	Prof. Philippa Jane Easterbrook		Dr. Elizabeth Hamlyn<br>Fatimah Karim<br>Kristin Kuldanek<br>Emma L. Macfarlane<br>Dr. Frank A. Post<br>Dr. Jan Welch<br>Dr. Tanya Welz	King's College Hospital<br>Caldecot Centre, Department of HIV<br>15-24 Caldecot Road<br>London, SE5 9RS<br>UNITED KINGDOM	Kings College Hospital Research Ethics Committee<br>Camberwell Building<br>King's College Hospital<br>94 Denmark Hill<br>LONDON, SE5 9RS<br>UNITED KINGDOM<br><br>South East Multi Centre Research Ethics Committee<br>Kent & Medway Health Authority<br>Preston Hall<br>Aylesford<br>Maidtone, Kent ME20 7NJ<br>UNITED KINGDOM
1082	Dr. Mark R. Nelson		Dr. Marta Boffito<br>Nicola Boyle<br>Mr. Carl Fletcher<br>Mr. Christopher J. Higgs<br>Dr. Akil George Anthony Jackson<br>Lucy Katso<br>Ngaire Latch<br>Dr. Desmond D. Maitland	Chelsea & Westminster Hospital (GU)<br>Thomas Macauley Ward, 2nd Floor<br>Fulham Road<br>London, SW10 9TH<br>UNITED KINGDOM	Riverside Research Ethics Committee<br>Room 3E03A<br>3rd Floor East, Charing Cross Hospital<br>Fulham Palace Road<br>London, W6 8RF<br>UNITED KINGDOM

* Did not randomize subjects

090177e1801b151d\Approved\Approved On: 03-Oct-2007 20:09

Center	Principal Investigator	Co-Investigator(s)	Sub-Investigator(s)	Address(es)	Institutional Review Board or Ethics Committee Address(es)
			Sifiso Mguni<br>Dr. Graeme J. Moyle<br>Jessica R. Osorio<br>Kasha Priya Singh<br>Dr. Alastair J. Teague<br>Jane Waters<br>Dr. Alan Winston<br>Mazen Yehya		South East Multi Centre Research Ethics Committee<br>Kent & Medway Health Authority<br>Preston Hall<br>Aylesford<br>Maidtone, Kent ME20 7NJ<br>UNITED KINGDOM
1100	Prof. Margaret Anne Johnson		Dr. Sanjay Rasiklal Bhagani<br>Mr. Patrick B. Byrne<br>Ms. Anne Carroll<br>Ms. Zoe L. Cuthbertson<br>Mr. Anthony Drinkwater<br>Dr. Samantha Ganeshaguru<br>Dr. Sabine Kinloch-de Loes<br>Dr. Sara Madge<br>Dr. Gabrielle Nora Murphy<br>Dr. Beth Prinz<br>Ms. Frances M. Turner<br>Dr. Mervyn John Tyrer<br>Anele Waters<br>Dr. Mike S. Youle	Royal Free Hospital<br>Thoracic Medicine - HIV/AIDS, Garrett Anderson Ward (11th Floor)<br>Pond Street<br>Hampstead<br>London, NW3 2QG<br>UNITED KINGDOM	ROYAL FREE HOSPITAL<br>POND STREET<br>HAMPSTEAD<br>LONDON<br>NW3<br>UNITED KINGDOM<br><br>South East Multi Centre Research Ethics Committee<br>Kent & Medway Strategic Health Authority<br>Preston Hall<br>Aylesford<br>Maidtone, Kent ME20 7NJ<br>UNITED KINGDOM

090177e1801b151d\Approved\Approved On: 03-Oct-2007 20:09

Center	Principal Investigator	Co-Investigator(s)	Sub-Investigator(s)	Address(es)	Institutional Review Board or Ethics Committee Address(es)
1108 *	Prof. Jonathan N. Weber		Dr. Tristan J. Barber<br>Bonaventure C. DeSouza<br>Mr Kenn Legg	St Mary's Hospital<br>Jeffriss Wing Pharmacy<br>Praed Street<br>London, W2 1NY<br>UNITED KINGDOM	South East Multi Centre Research Ethics Committee<br>Kent & Medway Strategic Health Authority<br>Preston Hall<br>Aylesford<br>Maidtone, Kent ME20 7NJ<br>UNITED KINGDOM<br><br>St Mary's Research Ethics Committee<br>St Mary's Hospital<br>Mailbox 121<br>141 Praed Street<br>London, W2 1NY<br>UNITED KINGDOM
1109	Dr. Edmund Wilkins		Dr. Alec Bonington<br>Dr. Susan Clarke<br>Dr. Edward Dunbar<br>Dr. Penelope Lewthwaite<br>Dr. Jane Mallewa<br>Dr. Libuse Ratcliffe<br>Ms. Andrea Robertson<br>Ms. Elaine Stockwell<br>Dr. Andrew P. Ustianowski<br>Dr. Fransisco Javier Vilar	North Manchester General Hospital<br>Clinical Trials Unit<br>Delauneys Road<br>Crumpsall<br>Manchester, M8 5RB<br>UNITED KINGDOM	Greater Manchester Strategic Health Authority<br>Room 181<br>!st Floor Gateway House<br>Picadilly South<br>Manchester, M60 7LP<br>UNITED KINGDOM<br><br>South East Multi Centre Research Ethics Committee<br>Kent & Medway Strategic Health Authority<br>Preston Hall<br>Aylesford<br>Maidtone, Kent ME20 7NJ<br>UNITED KINGDOM

* Did not randomize subjects

090177e1801b151d\Approved\Approved On: 03-Oct-2007 20:09

Center	Principal Investigator	Co-Investigator(s)	Sub-Investigator(s)	Address(es)	Institutional Review Board or Ethics Committee Address(es)
1192 *	Edmund Ong		Dr. Hamad Hadi<br>Dr. Brendan Payne<br>Dr. Nikhil Premchand<br>Dr. David Price<br>Dr. Matthias Schmid<br>Dr. Michael H. Snow<br>Dr. Jacob Wembri<br>Mrs. Joyce Wotherspoon	Newcastle General Hospital<br>Dept. of Infection and Tropical Medicine<br>Westgate Road<br>Newcastle Upon Tyre, NE4 6BE<br>UNITED KINGDOM	Newcastle & North Tyneside Research Ethics Committee 2<br>Room G14<br>Dental School<br>Framlington Place<br>Newcastle, NE2 4HH<br>UNITED KINGDOM<br><br>South East Multi Centre Research Ethics Committee<br>Preston Hall<br>Aylesford<br>Maidstone, Kent ME20 7NJ<br>UNITED KINGDOM
1208	Dr. Philip Hay		Aderonke Adebiyi<br>Dr. Richard Lau<br>Dr. Macky Natha<br>Dr. Muchaneta Ndoro<br>Dr. Mark Pakianathan<br>Dr. David R. Phillips<br>Dr. Melanie Rosenvinge<br>Dr. Tariq Sadiq	St. George's Hospital<br>Dept. of GU Medecine<br>Blackshaw Road<br>London, SW17 0QT<br>UNITED KINGDOM	South East Multi Centre Research Ethics Committee<br>Preston Hall<br>Aylesford<br>Maidstone, Kent ME20 7NJ<br>UNITED KINGDOM
1210 *	Dr. Martin Wiselka		Sally Batham<br>Ms. Susan Johnson	Leicester Royal Infirmary<br>Department of Infectious and Tropical Medicine<br>Ward 38<br>Leicester, LEICS LE1 5WW<br>UNITED KINGDOM	Leicestershire, Northamptonshire REC2<br>3rd Floor<br>Laurie House<br>Colyear Street<br>Derby, DE1 1LJ<br>UNITED KINGDOM<br><br>South East Multi Centre Research Ethics Committee<br>Kent & Medway Health Authority<br>Preston Hall<br>Park Row<br>Maidstone, Kent ME20 7NJ<br>UNITED KINGDOM

* Did not randomize subjects

090177e1801b151d\Approved\Approved On: 03-Oct-2007 20:09

United States

Coordinating Investigators:
<None Entered>

Center	Principal Investigator	Co-Investigator(s)	Sub-Investigator(s)	Address(es)	Institutional Review Board or Ethics Committee Address(es)
1130	Dr. Frances Wallach		Dr. Gabriela Rodriguez-Caprio	Mount Sinai Medical Center, Jack Martin Fund Clinic<br>One Gustave L. Levy Place<br>New York, NY 10029<br>UNITED STATES	Mount Sinai School of Medicine Institutional Review Board<br>Box 1075<br>One Gustave L. Levy Place<br>New York, NY 10029<br>UNITED STATES
1132	Dr. Barry Michael Rodwick		Ms. Lynne E. Merriam<br>Dr. Greg Marc Silver	Clinical Research of West Florida, Incorporated<br>2147 Northeast Coachman Road<br>Clearwater, FL 33765<br>UNITED STATES<br><br>Health Positive<br>Suite 1<br>3135 SR 580<br>Safety Harbor, FL 34695<br>UNITED STATES	Schulman Associates Institutional Review Board, Inc<br>4290 Glendale-Milford Road<br>Cincinnati, OH 45242<br>UNITED STATES
1133	Dr. Jeffrey Howard Burack		Dr. Claire Diane Borkert<br>Dr. Stephen Frank O'Brien	Alta Bates Summit Medical Center, East Bay AIDS Center<br>2nd Floor<br>3100 Summit St.<br>Oakland, CA 94609-3480<br>UNITED STATES	Alta Bates Institutional Review Board<br>2450 Ashby Avenue<br>Berkeley, CA 94705<br>UNITED STATES
1134	Dr. John Buscemi Montana			John B. Montana, MD<br>Suite 1G<br>30 5th Avenue<br>New York, NY 10011<br>UNITED STATES	Schulman Associates Institutional Review Board, Inc<br>4290 Glendale-Milford Road<br>Cincinnati, OH 45242<br>UNITED STATES
1137	Dr. Steven Santiago		Dr. Maria Gabriela Ale-Castro	Care Resource, Inc.<br>Suite 300<br>3510 Biscayne Boulevard<br>Miami, FL 33137<br>UNITED STATES	Schulman Associates Institutional Review Board, Inc<br>4290 Glendale-Milford Road<br>Cincinnati, OH 45242<br>UNITED STATES

090177e1801b151d\Approved\Approved On: 03-Oct-2007 20:09

Center	Principal Investigator	Co-Investigator(s)	Sub-Investigator(s)	Address(es)	Institutional Review Board or Ethics Committee Address(es)
1138 *	Dr. Ana Maria Alvarez-Jacinto		Gloria E. Alonso<br>Dr. Josefa L. Binker	MedSearch Professional Group<br>Suite 602<br>1800 Southwest 27th Avenue<br>Miami, FL 33145<br>UNITED STATES	Schulman Associates Institutional Review Board, Inc<br>4290 Glendale-Milford Road<br>Cincinnati, OH 45242<br>UNITED STATES
1140	Dr. Ricky K. Hsu			Ricky K. Hsu, MD - Private Practice<br>4th Floor<br>154 West 14th Street<br>New York, NY 10011<br>UNITED STATES	Schulman Associates Institutional Review Board, Inc<br>4290 Glendale-Milford Road<br>Cincinnati, OH 45242<br>UNITED STATES
1142	Dr. Cynthia A. Mayer		Dr. Margarita R. Cancio<br>Ms. Tish I. Carlton<br>Dr. Michael Edward Dunn<br>Ms. Lois A. Hall<br>Ms. Tara Hamilton<br>Mr. Kenneth C. Kavanagh<br>Dr. Nadeem R. Khan<br>Dr. Dorece G. Norris<br>Ms. Bonnie Peterson<br>Bonnie F. Tiemann<br>Dr. Scott S. Ubillos	Dorece Norris, MD and Michael Dunn, MD<br>Suite A<br>4205 South MacDill<br>Tampa, FL 33611<br>UNITED STATES<br><br>Pinellas Care Clinic<br>3554 1st Avenue, North<br>St. Petersburg, FL 33713<br>UNITED STATES<br><br>St. Joseph's Comprehensive Research Institute<br>Suites 3 & 5<br>4200 North Armenia Avenue<br>Tampa, FL 33607<br>UNITED STATES	St. Joseph's Hospital Institutional Review Board<br>3001 West Martin Luther King, Jr. Boulevard<br>Tampa, FL 33607<br>UNITED STATES

* Did not randomize subjects

090177e1801b151d\Approved\Approved On: 03-Oct-2007 20:09

Center	Principal Investigator	Co-Investigator(s)	Sub-Investigator(s)	Address(es)	Institutional Review Board or Ethics Committee Address(es)
1143	Dr. Rodger David Macarthur		Patricia Arlauskas<br>Dr. Jonathan Allen Cohn<br>Dr. Lawrence R. Crane<br>Karin Dunbar<br>Marti Farrough<br>Varsha Moudgal	Wayne State University<br>Infectious Diseases Clinic<br>UHC-7B & C<br>4201 Saint Antoine<br>Detroit, MI 48201<br>UNITED STATES<br><br>Wayne State University<br>UHC-7D<br>4201 St. Antoine<br>Detroit, MI 48201<br>UNITED STATES	Wayne State University Human Investigation Committee<br>101 East Alexandrine Building<br>Detroit, MI 48201<br>UNITED STATES
1157	Dr. Shubha Kerkar		Mr. Steven Kindel<br>Dr. John Alex Kruba<br>Dr. Natalie Shemonsky<br>Karin Starr	Desert Medical Group, Clinical Research Center<br>B-5<br>275 North El Cielo Road<br>Palm Springs, CA 92262<br>UNITED STATES	Schulman Associates Institutional Review Board, Inc<br>4290 Glendale-Milford Road<br>Cincinnati, OH 45242<br>UNITED STATES
1158	Dr. Getachew Feleke		Dr. Mino Absy-Jaghab<br>Dr. Shadab Ahmed<br>Ms. Christine Cervini<br>Yuwanna Landau<br>Dr. Janice Verley<br>Dr. Tabbasum Yasmin	Nassau University Medical Center<br>E Building, Room 244<br>2201 Hempstead Turnpike<br>East Meadow, NY 11554<br>UNITED STATES	NUMC IRB<br>2201 Hempstead Turnpike<br>East Meadow, NY 11554<br>UNITED STATES
1159	Dr. Jeffrey Eliot Galpin			Shared Medical Research Foundation<br>Suite 320<br>5620 Wilbur Avenue<br>Tarzana, CA 91356<br>UNITED STATES	Schulman Associates Institutional Review Board, Inc<br>4290 Glendale-Milford Road<br>Cincinnati, OH 45242<br>UNITED STATES
1161	Dr. Catherine Maria Creticos		Dr. Roger Trinh	Howard Brown Health Center<br>4025 North Sheridan Road<br>Chicago, IL 60613<br>UNITED STATES	Schulman Associates Institutional Review Board, Inc<br>4290 Glendale-Milford Road<br>Cincinnati, OH 45242<br>UNITED STATES

* Did not randomize subjects

090177e1801b151d\Approved\Approved On: 03-Oct-2007 20:09

Center	Principal Investigator	Co-Investigator(s)	Sub-Investigator(s)	Address(es)	Institutional Review Board or Ethics Committee Address(es)
1164	Dr. Robert S. Jones Jr.		Dr. Wendy Babitt<br>Dr. Ann Kyungwohn Shin<br>Dr. Ambreen Umer	RPS Infectious Diseases<br>Suite 130<br>301 South 7th Avenue<br>West Reading , PA 19611<br>UNITED STATES	The Reading Hospital and Medical Center's Institutional Review Board<br>6th and Spruce Streets<br>N-ground<br>West Reading, PA 19610<br>UNITED STATES
1165	Dr. Daniel Edward Nixon		Dr. Gregory C. Childress<br>Mr. Robert T. Higginson Jr.	Richmond AIDS Consortium<br>20th Floor, Suite 2020<br>Main Street Centre, 600 East Main Street<br>Richmond, VA 23219<br>UNITED STATES<br><br>VCU Health Systems<br>Infectious Disease Clinic<br>3rd Floor, West Hospital<br>1200 East Broad Street<br>Richmond, VA 23298<br>UNITED STATES	Western Institutional Review Board<br>3535 Seventh Avenue, SW<br>Olympia, WA 98508<br>UNITED STATES
1167	Dr. Alexander A. McMeeking			Alexander A. McMeeking, MD<br>Suite 507<br>104 East 40th Street<br>New York, NY 10016<br>UNITED STATES	Schulman Associates IRB, Inc.<br>4290 Glendale-Milford Road<br>Cincinnati, OH 45242<br>UNITED STATES
1169	Dr. Victoria Lee Sharp		Dr. Stephen Arpadi<br>Dr. Vani Gandhi<br>Phyllis Ristau<br>Dr. Lia Tsveniashvili<br>Mr. Robert Warford	St. Luke's Roosevelt Hospital Center<br>Morningside Clinic 3rd Floor<br>1111 Amsterdam Avenue<br>New York, NY 10025<br>UNITED STATES	St. Luke's- Roosevelt Institute of Health Sciences<br>Research Administration- AJA 207<br>432 West 58th Street<br>New York, NY 10019<br>UNITED STATES
1171	Dr. Leon Liang-Yu Lai		Dr. Maria Ruiz<br>Dr. Margo Ann Smith	Washington Hospital Center<br>110 Irving Street Northwest<br>Washington, DC 20010-2976<br>UNITED STATES	Medstar Research Institute<br>Suite 201<br>6495 New Hampshire Avenue<br>Hyattsville, MD 20783<br>UNITED STATES

* Did not randomize subjects

090177e1801b151d\Approved\Approved On: 03-Oct-2007 20:09

090177e1801b151d\Approved\Approved On: 03-Oct-2007 20:09

Center	Principal Investigator	Co-Investigator(s)	Sub-Investigator(s)	Address(es)	Institutional Review Board or Ethics Committee Address(es)
1172 *	Dr. Thomas Mc Donald File Jr.		Dr. Hector Fabio Bonilla<br>Dr. Amy A. Hite<br>Dr. Joseph Paul Myers<br>Dr. Michael J. Tan	C.A.R.E. Center<br>Suite 104<br>75 Arch Street<br>Akron, OH 44304<br>UNITED STATES	Summa Health System Institutional Review Board<br>525 E. Market St<br>Akron, Ohio 44309<br>UNITED STATES
1173	Dr. Frances Fae Haas			University of Oklahoma College of Medicine- Tulsa<br>2815 South Sheridan Road<br>Tulsa, OK 74129<br>UNITED STATES	University of Oklahoma Health Science Center Institutional Review Board<br>Robert M. Bird Library<br>1000 S.L. Young Blvd.<br>Oklahoma City, OK 73190-3046<br>UNITED STATES
1174	Dr. Winkler Weinberg		Gayle Arberg<br>Dr. William T. Blake<br>Dr. Lee David Jacobs	Kaiser Permanente<br>Cumberland Medical Offices<br>Suite K<br>2525 Cumberland Parkway<br>Atlanta, GA 30339-3915<br>UNITED STATES<br><br>Kaiser Permanente<br>Southwood Medical Offices<br>400 Mt. Zion Parkway<br>Jonesboro, GA 30236<br>UNITED STATES	Kaiser Permanente Insitutional Review Board Regional Office<br>suite 205<br>Ten Piedmont Center<br>3495 Piedmont Road NE<br>Atlanta, GA 30305<br>UNITED STATES
1175	Dr. Norman Peter Markowitz		Dr. Indira Brar	Henry Ford Hospital<br>2799 West Grand Boulevard<br>Detroit, MI 48202<br>UNITED STATES	Henry Ford Hospital Institutional Review Board<br>Research Administration<br>2799 West Grand Boulevard<br>Detroit, MI 48202<br>UNITED STATES
1176 *	Dr. Peter Jerome Ruane Jr.		Dr. Peter Rice Wolfe	Peter J. Ruane, M.D. Inc.<br>Suite 401<br>5901 West Olympic Boulevard<br>Los Angeles, CA 90036<br>UNITED STATES	Schulman Associates IRB, Inc.<br>4290 Glendale-Milford Road<br>Cincinnati, OH 45242<br>UNITED STATES

* Did not randomize subjects

Center	Principal Investigator	Co-Investigator(s)	Sub-Investigator(s)	Address(es)	Institutional Review Board or Ethics Committee Address(es)
1180	Dr. Chiu-Bin Hsiao		Ms. Laurie Abbatessa<br>Dr. Naomi S. Boston<br>Deborah Graf<br>Dr. Adel Sulaiman	Erie County Medical Center, HIV Services<br>462 Grider Street/Tunnel<br>Buffalo, NY 14215<br>UNITED STATES	Health Sciences Institutional Review Board<br>150 Parker Hall<br>3435 Main Street<br>Buffalo, NY 14214<br>UNITED STATES
1182	Patrick G. Clay		Dr. Gautam J. Desai<br>Dr. David D. Dyck Jr.<br>Dr. Ann M. Karty<br>Dr. Carol E. Kirila<br>Dr. Patrick M. Nemechek<br>Dr. Richard K. Ogden Sr.<br>Dr. Sandra Kay Willsie	Kansas City University of Medicine and Biosciences<br>1750 Independence Avenue<br>Kansas City, MO 64106<br>UNITED STATES	Kansas City University of Medicine and Biosciences IRB<br>1750 Independence Avenue<br>Kansas City, MO 64106<br>UNITED STATES
1183	Dr. Janak Koirala		Dr. Tin Han Htwe<br>Dr. Nancy M. Khardori<br>Dr. Adnan Mushtaq<br>Dr. Cristian Speil	Southern Illinois University School of Medicine<br>Division of Infectious Diseases<br>701 North First Street<br>Springfield, IL 62702<br>UNITED STATES	Springfield Committee for Research Involving Human Subjects<br>P.O. Box 19616<br>801 North Rutledge Street<br>Springfield, IL 62702<br>UNITED STATES
1184	Dr. Barbara J. Hanna		Dr. Raul R. Magadia	Health Services Center<br>608 Martin Luther King Drive<br>Anniston, AL 36201<br>UNITED STATES	Schulman Associates IRB, Inc.<br>4290 Glendale-Milford Road<br>Cincinnati, OH 45242<br>UNITED STATES
1185	Dr. L. W. Preston Church		Dr. Dannah Wilde Wray	Medical University of South Carolina<br>171 Ashley Avenue<br>Charleston, SC 29425<br>UNITED STATES<br><br>Medical University of South Carolina<br>Infectious Diseases Clinic<br>PO Box 250555<br>135 Rutledge Avenue<br>Charleston, SC 29403<br>UNITED STATES	Office of Research Integrity<br>Suite 501, PO Box 250857<br>165 Cannon Street<br>Charleston, SC 29425<br>UNITED STATES

* Did not randomize subjects

090177e1801b151d\Approved\Approved On: 03-Oct-2007 20:09

Center	Principal Investigator	Co-Investigator(s)	Sub-Investigator(s)	Address(es)	Institutional Review Board or Ethics Committee Address(es)
1186	Dr. Joseph G. Timpone Jr.		Dr. Gayle P. Balba<br>Dr. Princy Nirmal Kumar<br>Dr. Adam I. Sherwat	Georgetown University Hospital<br>Kober-Cogan Building<br>Suite 110<br>3800 Reservoir Road, NW<br>Washington, DC 20007<br>UNITED STATES	Georgetown University Institutional Reveiw Board<br>SW 104 Medical - Dent Building<br>3900 Reservoir Road North West<br>Washington, DC 20007<br>UNITED STATES
1187	Dr. Patrick L. McLeroth<br>Dr. Peter Kadlecik (Previous PI)		Ms. Gaetane Delva-Brunson<br>Dr. Emily Leach Richie<br>William J. Schaefer	Chase Brexton Health Services<br>1001 Cathedral Street<br>Baltimore, MD 21201<br>UNITED STATES	Schulman Associates IRB, Inc.<br>4290 Glendale-Milford Road<br>Cincinnati, OH 45242<br>UNITED STATES
1194	Dr. Diana Antoniskis		Dr. Colleen Finnegan<br>Dr. Joel S. Godbey<br>Dr. Keith Bernard Riley<br>Dr. Molly Stenzel	Kaiser Permanente-Northwest Region, Immune Deficiency Clinic<br>3550 North Interstate Avenue<br>Portland, OR 97227<br>UNITED STATES	Kaiser Permanente Northwest Institutional Review Board<br>3800 North Interstate<br>Portland, OR 97227<br>UNITED STATES
1195	Dr. Stephen Lloyd Green			Hampton Roads Medical Specialists<br>2112 Executive Drive<br>Hampton, VA 23666<br>UNITED STATES	Schulman Associates IRB, Inc.<br>4290 Glendale-Milford Road<br>Cincinnati, OH 45242<br>UNITED STATES
1196	Dr. Christine Zurawski		Dr. Ronald Devine<br>Dr. Richard Hengel<br>Mr. Steven Schmitt	Infectious Disease Solutions, PC<br>Suite M245<br>35 Collier Road<br>Atlanta, GA 30309<br>UNITED STATES	Schulman Associates IRB, Inc.<br>4290 Glendale-Milford Road<br>Cincinnati, OH 45242<br>UNITED STATES

090177e1801b151d\Approved\Approved On: 03-Oct-2007 20:09

* Did not randomize subjects

Center	Principal Investigator	Co-Investigator(s)	Sub-Investigator(s)	Address(es)	Institutional Review Board or Ethics Committee Address(es)
1197	Dr. James Riddell IV		Dr. Carol Elizabeth Chenoweth<br>Dr. Sandro Kurt Cinti<br>Dr. Daniel Kaul<br>Dr. Powel Kazanjian	University of Michigan Health System<br>Infectious Diseases Clinic<br>3330 Taubman Center<br>Ann Arbor, MI 48109-0352<br>UNITED STATES<br><br>University of Michigan Health System<br>Investigational Drug Services<br>UH B2D400<br>1500 East Medical Center Drive<br>Ann Arbor, MI 48109-0008<br>UNITED STATES	Institutional Review Board for Human Subjects Research<br>University of Michigan Medical School<br>Argus 1 Building<br>517 West William Street<br>Ann Arbor, MI 48103-4943<br>UNITED STATES
1198	Dr. Daniel S. Berger		Dr. Gary Gerard Bucher<br>Mr. Curtis P. Hainds<br>Dr. Robert Todd Hargan<br>Dr. Kaleoviokawai Staszkow<br>Dr. Andrew H. Zalski	Northstar Medical Center<br>Suite 500<br>2835 N. Sheffield Avenue<br>Chicago, IL 60657<br>UNITED STATES	Schulman Associates IRB, Inc.<br>4290 Glendale-Milford Road<br>Cincinnati, OH 45242<br>UNITED STATES
1199	Dr. Robert Charles Kalayjian			Metro Health Systems<br>Cancer Care Pavilion Rm 2001<br>2500 Metrohealth Drive<br>Cleveland, OH 44109<br>UNITED STATES	MetroHealth Medical Center<br>Institutional Review Board<br>Room 103<br>2500 MetroHealth Drive<br>Cleveland, OH 44109<br>UNITED STATES
1203 *	Dr. Leonel Perez-Limonte			AppleMed Research, Inc.<br>6850 Coral Way #409<br>Miami , FL 33155<br>UNITED STATES	Schulman Associates IRB, Inc.<br>4290 Glendale-Milford Road<br>Cincinnati, OH 45242<br>UNITED STATES

* Did not randomize subjects

090177e1801b151d\Approved\Approved On: 03-Oct-2007 20:09

090177e1801b151d\Approved\Approved On: 03-Oct-2007 20:09

Center	Principal Investigator	Co-Investigator(s)	Sub-Investigator(s)	Address(es)	Institutional Review Board or Ethics Committee Address(es)
1204	Dr. Gary Blick		Ms Patricia R. Garton<br>Dr. Paula Rosa Greiger-Zanlungo	Circle Medical, LLC<br>Suite 32<br>153 East Avenue<br>Norfolk, CT 06851<br>UNITED STATES<br><br>Private Practice - Gary Blick, MD<br>Suite 32<br>153 East Avenue<br>Norwalk, CT 06851<br>UNITED STATES	Schulman Associates IRB, Inc.<br>4290 Glendale-Milford Road<br>Cincinnati, OH 45242<br>UNITED STATES
1205	Dr. Barbara A. Atkinson		Dr. Stephen E. Weis	Preventive Medicine Clinic<br>Tarrant County Public Health<br>Room 1500<br>1101 South Main Street<br>Fort Worth, TX 76104<br>UNITED STATES<br><br>University of North Texas Health Science Center at Fort Worth<br>Department of Internal Medicine<br>855 Montgomery Street<br>Fort Worth, TX 76107<br>UNITED STATES	University of North Texas Health Science Center at Forth Worth<br>Institutional Review Board<br>999 Montgomery Street<br>Forth Worth, TX 76107<br>UNITED STATES
1207	Dr. David James Prelutsky			Southampton Healthcare, Inc.<br>2340 Hampton Avenue<br>Saint Louis, MO 63139-2909<br>UNITED STATES	Schulman Associates IRB, Inc.<br>4290 Glendale-Milford Road<br>Cincinnati, OH 45242<br>UNITED STATES
1211 *	Amy Seinfeld		Dr. Juan Gonzales	Neworld Health Care<br>Suite 704<br>407 Lincoln Road<br>Miami Beach, FL 33139<br>UNITED STATES	Schulman Associates IRB, Inc.<br>4290 Glendale-Milford Road<br>Cincinnati, OH 45242<br>UNITED STATES

* Did not randomize subjects

Center	Principal Investigator	Co-Investigator(s)	Sub-Investigator(s)	Address(es)	Institutional Review Board or Ethics Committee Address(es)
1212	Dr. Patrick William Daly			Nelson Tebedo Health Resource Center<br>4012 Cedar Springs<br>Dallas, TX 75219<br>UNITED STATES<br><br>Private Practice Patrick W. Daly MD<br>3629 Fairmont Street<br>Dallas, TX 75219<br>UNITED STATES	Schulman Associates IRB, Inc.<br>4290 Glendale-Milford Road<br>Cincinnati, OH 45242<br>UNITED STATES
1216	Dr. Robert H. Keller		Jamie L. Dickerson<br>Dr. James N. Luckett	Biodoron Medical Center<br>5821 Hollywood Boulevard<br>Hollywood, FL 33021-6327<br>UNITED STATES	Schulman Associates IRB, Inc.<br>4290 Glendale-Milford Road<br>Cincinnati, OH 45242<br>UNITED STATES
1217 *	Dr. Jesse Pullen Penico		Dr. Irwin Joseph Trestman	Administrative Office Only - Clinical Trials Management, LLC<br>Suite 405<br>Medical Plaza II<br>3901 Houma Blvd.<br>Metairie, LA 70006<br>UNITED STATES<br><br>Jesse P. Penico, MD<br>Ste. 202<br>Medical Plaza I<br>3901 Houma Blvd.<br>Metairie, LA 70006<br>UNITED STATES	Schulman Associates IRB, Inc.<br>4290 Glendale-Milford Road<br>Cincinnati, OH 45242<br>UNITED STATES
1218	Dr. Barbara H. Wade		Catherine Godwin<br>Belinda Soltz<br>Michael Lawton ARNP	Wade, Barbara H MD<br>Ste 305<br>5153 N 9th Ave.<br>Pensacola, FL 32504-5719<br>UNITED STATES	Schulman Associates IRB, Inc.<br>4290 Glendale-Milford Road<br>Cincinnati, OH 45242<br>UNITED STATES

* Did not randomize subjects

090177e1801b151d\Approved\Approved On: 03-Oct-2007 20:09

Center	Principal Investigator	Co-Investigator(s)	Sub-Investigator(s)	Address(es)	Institutional Review Board or Ethics Committee Address(es)
1221	Dr. Mark Leslie Tanner			Family Health Care of Atlanta<br>1935 Howell Mill Rd NW<br>Atlanta, GA 30318-2513<br>UNITED STATES	Schulman Associates IRB, Inc.<br>4290 Glendale-Milford Road<br>Cincinnati, OH 45242<br>UNITED STATES
1222	Dr. Sujata Lalla-Reddy		Todd A. Forster<br>Dr. Kenneth B. Horwitz	Pacific Coast Specialty Group<br>Suite 129<br>12062 Valley View Street<br>Garden Grove, CA 92845<br>UNITED STATES	Schulman Associates IRB, Inc.<br>4290 Glendale-Milford Road<br>Cincinnati, OH 45242<br>UNITED STATES
1225	Dr. James Sampson			James H. Sampson MD<br>The Research and Education Group<br>Suite 185<br>1650 Northwest Naito Parkway<br>Portland, OR 97209-2535<br>UNITED STATES	Schulman Associates IRB, Inc.<br>4290 Glendale-Milford Road<br>Cincinnati, OH 45242<br>UNITED STATES
1226 *	Dr. Zaher Shebib			Bay Area Infectious Diseases Associates, P.A.<br>Suite 201<br>6319 Fairmont Parkway<br>Pasadena , TX 77505-4245<br>UNITED STATES	Schulman Associates IRB, Inc.<br>4290 Glendale-Milford Road<br>Cincinnati, OH 45242<br>UNITED STATES
1229	Dr. Lynette H. Posorske		Dr. Phuong Duc Trinh	Dr. Alpert, Trinh, and Posorske<br>Suite 230<br>8630 Fentron Street<br>Silver Spring, MD 20910<br>UNITED STATES	Schulman Associates IRB, Inc.<br>4290 Glendale-Milford Road<br>Cincinnati, OH 45242<br>UNITED STATES

* Did not randomize subjects

090177e1801b151d\Approved\Approved On: 03-Oct-2007 20:09

Center	Principal Investigator	Co-Investigator(s)	Sub-Investigator(s)	Address(es)	Institutional Review Board or Ethics Committee Address(es)
1230	Dr. Goran Miljkovic		Silvia Dicovich<br>Dr. Zane K. Saul	Infectious Diseases Associates<br>2600 Post Road<br>Southport, CT 06490<br>UNITED STATES<br><br>Infectious Diseases Associates<br>Second Floor Suite D<br>2890 Main Street<br>Stratford, CT 06614<br>UNITED STATES	Schulman Associates IRB, Inc.<br>4290 Glendale-Milford Road<br>Cincinnati, OH 45242<br>UNITED STATES
1232	Dr. Paul Peniston Cook		Dr. Tahir Farooq<br>Marnie M. Jones<br>Robert E. Lawrence<br>Dr. Ricardo A. Maldonado	Brody School of Medicine at East Carolina University (ECU)<br>Division of Infectious Diseases and International Traveler's Clinic<br>Doctor's Park 6A<br>Greenville, NC 27834<br>UNITED STATES	University and Medical Center Institutional Review Board East Carolina University<br>Edward Warren Life Sciences Building<br>LSB 104<br>600 Moye Boulevard<br>Greenville, NC 27834<br>UNITED STATES
1234 *	Dr. Gregory John Malanoski		Joan M. Cain	AMS Infectious Disease<br>Suite 108<br>600 Ivy Street<br>Elmira, NY 14905<br>UNITED STATES<br><br>Arnot Ogden Medical Center - AMS Clinical Research<br>2nd Floor<br>602 Ivy Street<br>Elmira, NY 14905<br>UNITED STATES	Schulman Associates IRB, Inc.<br>4290 Glendale-Milford Road<br>Cincinnati, OH 45242<br>UNITED STATES

* Did not randomize subjects

090177e1801b151d\Approved\Approved On: 03-Oct-2007 20:09

Center	Principal Investigator	Co-Investigator(s)	Sub-Investigator(s)	Address(es)	Institutional Review Board or Ethics Committee Address(es)
1237	Dr. Robert Owen Brennan		Debra Erickson<br>Dr. James S. Wade	Infectious Diseases Associates of Central Virginia<br>2215 Landover Place<br>Lynchburg, VA 24501<br>UNITED STATES<br><br>Infectious Diseases Associates of Central Virginia<br>Suite 2200<br>201 South Main Street<br>Danville, VA 24541<br>UNITED STATES	Schulman Associates IRB, Inc.<br>4290 Glendale-Milford Road<br>Cincinnati, OH 45242<br>UNITED STATES

090177e1801b151d\Approved\Approved On: 03-Oct-2007 20:09
